# Translation of Deoxyribonucleic Acid into Synthetic Alpha Helical Peptides for Darwinian Evolution

**DOI:** 10.1021/jacsau.4c00738

**Published:** 2024-10-02

**Authors:** Millicent Dockerill, Pramod M. Sabale, Francesco Russo, Sofia Barluenga, Nicolas Winssinger

**Affiliations:** Department of Organic Chemistry, Faculty of Sciences, University of Geneva, Geneva 1211, Switzerland

**Keywords:** DEL, chemical evolution, PNA, MDM2, constrained peptides

## Abstract

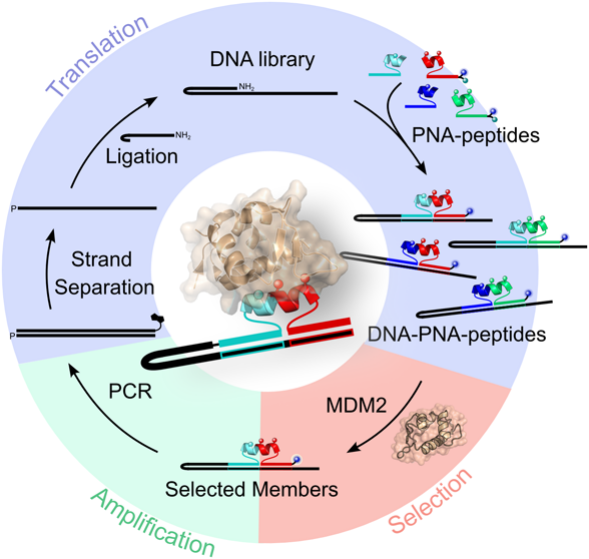

DNA-encoded libraries connect the phenotypes of synthetic molecules to a DNA barcode; however, most libraries do not tap into the potential of Darwinian evolution. Herein, we report a DNA-templated synthesis (DTS) architecture to make peptides that are stabilized into α-helical conformations via head-to-tail supramolecular cyclization. Using a pilot library targeting MDM2, we show that repeated screening can amplify a binder from the lowest abundance in the library to a ranking that correlates to binding affinity. The study also highlights the need to design libraries such that the chemistry avoids biases from the heterogeneous yield in DTS.

## Introduction

DNA-encoded libraries (DELs)^1−4^ represent a transformative technology in the field of drug discovery, providing an unprecedented capacity to screen vast chemical spaces with high throughput and efficiency (for recent examples, see refs (5−12)). DELs, comprising a diverse array of small molecules each tagged with a unique DNA sequence, enable the rapid identification of potential ligands by affinity selection and quantification of the tags. However, in contrast to natural selection, most DELs are fundamentally limited by the absence of a Darwinian selection mechanism.^13^ In natural evolutionary processes, molecules undergo continuous cycles of selection, replication (amplification), and mutation, allowing for the progressive enrichment of entities with superior binding and functional properties. While iterative selection cycles are frequently leveraged in biochemical selection technologies (phage display,^14−18^ SELEX,^19,20^ mRNA display,^21−24^ SICLOPPS^25^), its implementation in DELs requires replication of selected members, i.e., a mechanism to translate DNA sequence into synthetic molecules. The most practiced DEL synthesis format makes use of split and mix combinatorial synthesis^26^ with enzymatic ligation of DNA tags at each step.^27,28^ While very efficient from a synthesis perspective, it does not allow us to “translate” the DNA sequence of selected members into their encoded molecules. Alternative DEL synthetic strategies that are compatible with translation include DTS,^29−32^ sequence-encoded routing of DNA,^33−37^ yoctoliter-scale DNA reactor,^38^ and DNA-templated self-assembly of fragments.^39,40^ However, the benefits of reiterative selection/amplification cycles have not been thoroughly investigated experimentally, despite the potential of these technologies to remove false positives^41^ from screens and amplify low abundance library members.

**Figure 1:**
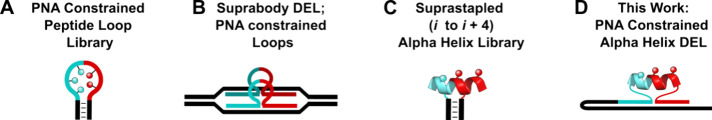
Examples of previous PNA-peptide conjugates that induce folding through hybridization.

PNA has also been utilized as a DNA analogue in DEL. Alongside its function as an encoding tag, PNA has been employed to fold PNA-peptide-PNA conjugates into loops through hybridization^40,42,43^ reminiscent of the proteogenic loops emanating from β-pleaded sheets or into α-helices (Figure 1A–C).^44^ Recently, we demonstrated that the self-assembly of such peptidic loops into dimers could be selected and replicated for Darwinian evolutionary cycles.^40^ Herein we report a hybridization architecture compatible with DNA-templated synthesis (DTS) that stabilizes an α-helical conformation (Figure 1D). We demonstrate a cycle of translation, selection, and amplification and used this workflow to study the Darwinian selection of a pilot library targeting MDM2, a target that binds to α-helical and stapled peptides.

## Results and Discussion

**Figure 2:**
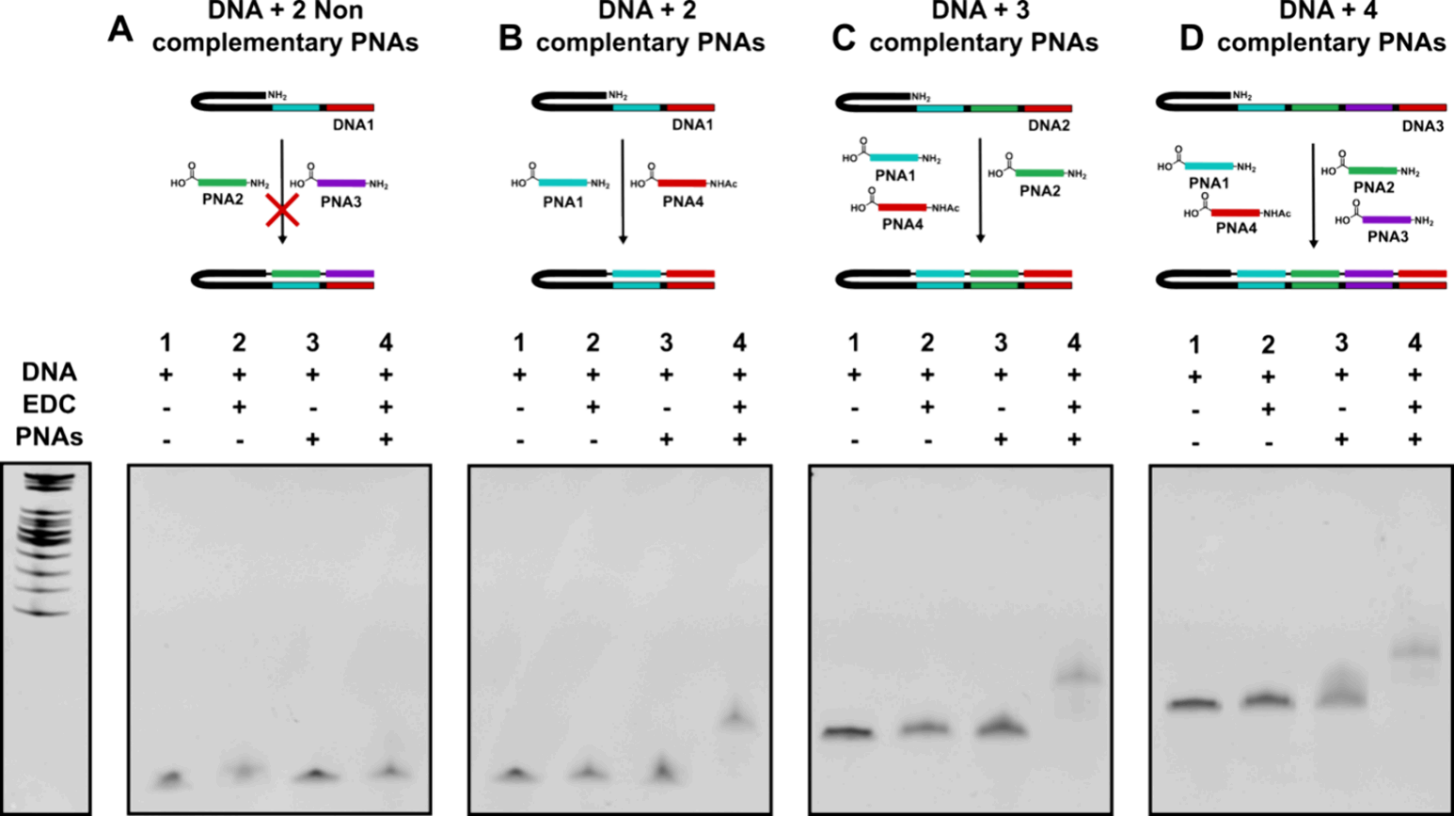
EDC-mediated DNA-PNA reaction. (A) Background reaction between **DNA1** (black harpin −38 mer) and two noncomplementary PNAs (**PNA2** (green) + **PNA3** (purple)). (B) Templated reaction between **DNA1** (black harpin −38 mer) and two complementary PNAs (**PNA1** (cyan) + **PNA4** (red)). (C) Templated reaction between **DNA2** (black harpin −47 mer) and three complementary PNAs (**PNA1** (cyan) + **PNA2** (green) + **PNA4** (red)). (D) Templated reaction between **DNA3** (black harpin −56 mer) and four complementary PNAs (**PNA1** (cyan) + **PNA2** (green) + **PNA3** (purple) + **PNA4** (red)). Lane 1: DNA alone. Lane 2: DNA + EDC. Lane 3: DNA + PNA. Lane 4: DNA + EDC + PNA. Denaturing (8 M UREA) 15% PAGE, 100 bp ladder is shown on the left, ethidium bromide staining.

To prepare a combinatorial DEL of PNA-constrained α-helix conjugates, as illustrated in Figure 1D, we needed to strategically combine a DNA-templated library with two types of PNA-peptide conjugate fragments: left-hand side (LHS) fragments are shown in cyan, and right-hand side (RHS) fragments are shown in red. The goal was to achieve DNA-templated amide bond formations between the DNA and the PNA in the LHS conjugate as well as between the peptides in each fragment (LHS and RHS). Using amide bond formation for ligation, as opposed to other chemistries, was deemed a prerequisite to ensure the correct hydrogen-bond pattern necessary for achieving the helical conformation of the peptides. While several examples of DNA-templated amide bond reactions have been reported,^45,46^ these reactions were performed at the blunt ends of DNA and this work would require these reactions to proceed at sites along continuously hybridized segments. As a model for DNA-templated ligations, we started with a minimal number of PNA conjugates. Thus, we first investigated the efficiency of EDC-mediated amide bond formation between DNA, functionalized at the 5′ end with an amino group, folded as a hairpin, and PNAs bearing free carboxylic acids at the C-terminus (a glycine residue) and an amino group at the N-terminus (Figure 2). We opted for 9-mer PNA (*K*_D_ 100–500 nM at 23 °C) as the minimum length to yield a sufficient duplex equilibrium under the reaction conditions (1 μM), allowing reactions to proceed with a small excess of PNAs (1.2 equiv). As shown in Figure 2, at this concentration, untemplated reactions are too slow and reactions with noncomplementary PNA (PNA2 + PNA3 + DNA1) did not yield hairpin primer extension (Figure 2A). However, with PNAs complementary to the template (PNA1 + PNA4 + DNA 1), high yields were observed (>90% based gel quantification) for ligations of two, three, and four contiguous PNAs (Figure 2B–D, respectively). An EDC concentration of 50 mM was found to be sufficient for the reactions; lower concentrations (5 mM) yielded incomplete reactions suggesting that carboxylic acid activation is rate limiting under these conditions (Figure S2). These results parallel the pioneering work reported by Liu et al. to translate DNA into synthetic polymers (CuAAC ligation),^32^ or PNA aptamers (reductive amination)^31^ but extend it to amide-bond formation.

**Figure 3:**
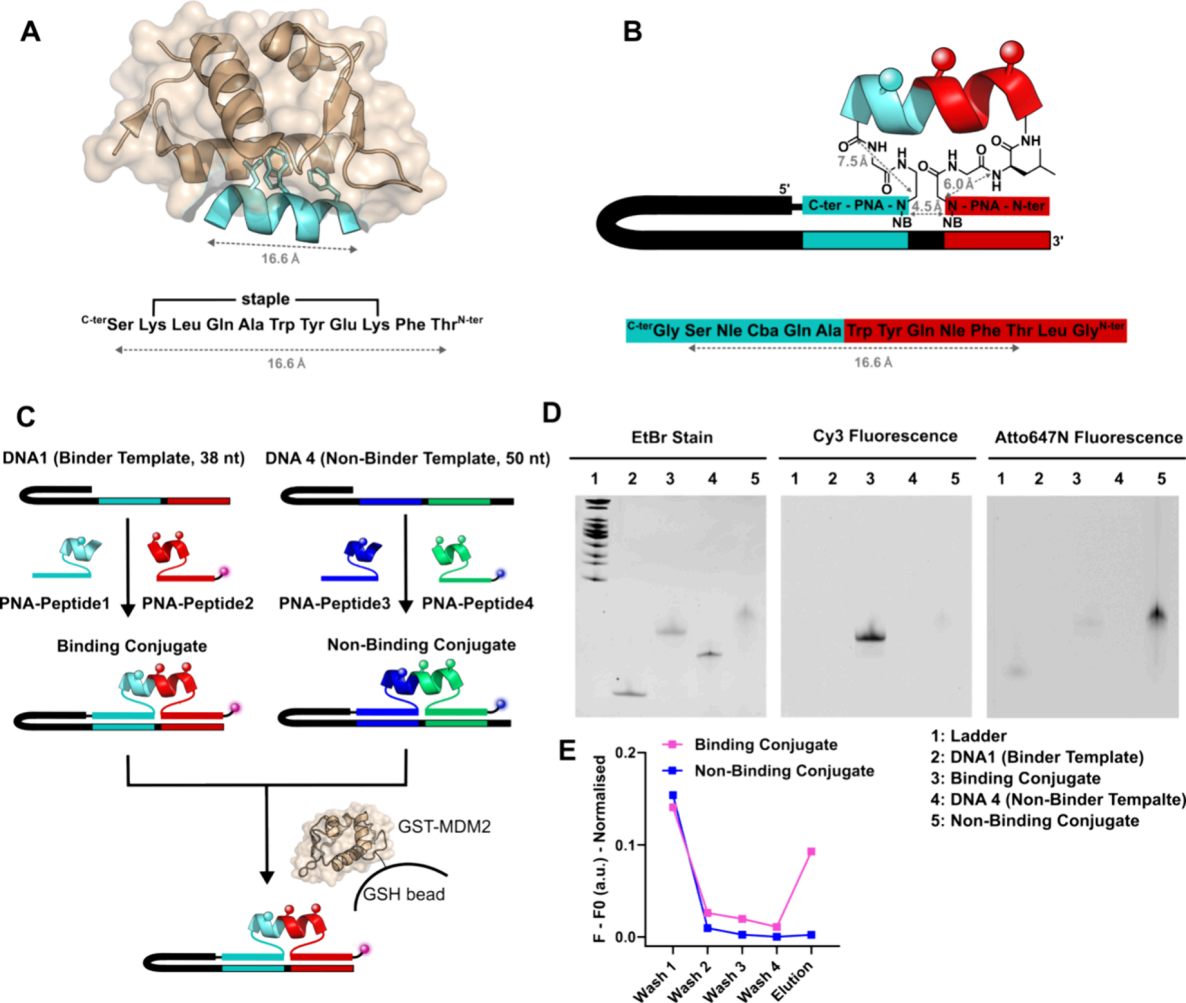
PNA display of alpha helices and their selection against MDM2. (A) Structure of the MDM2-peptide complex highlighting the key interaction of three residues with the target (image generated from PDB ID: 5AFG). The distance of 16.6 Å is measured from the carbonyl of Ser to the α-amino group of Thr. (B) Analysis of the linker length required between the encoding PNA and the peptide to accommodate an α-helical conformation. The distance includes the spacing between two nucleobases (NB). (C) PNA display of alpha helices, which either bind (Cy3-tagged binding conjugate) or do not bind (Atto647N tagged nonbinding conjugate) MDM2. (D) The templated reaction is shown for both constructs by gel (lane 2–5). Cy3 fluorescence, Atto647N fluorescence and EtBr staining are displayed. 8 M UREA 15% PAGE. (E) Plate reader fluorescence of washes and elution of affinity selection are also shown.

We next investigated whether a templated ligation could yield peptides in an α-helical conformation. Since CD-based analyses would be difficult to interpret given the PNA, we opted to assess the helical nature of the peptide using binding to a target that requires this conformation. To this end, we used MDM2, an important regulator of the p53 tumor suppressor, which binds to an α-helix of p53^47^ and has been a testing ground for stapling technologies^48−52^ and screening technologies.^53−56^ Additionally, several X-ray diffraction structures of the MDM2-peptide complex have been reported, supporting the importance of the helicity of the peptide.^51,57,58^ We selected the peptide reported by Spring et al.^51^ as our starting point (Figure 3A), replacing the residues to be stapled (lysine(N_3_)) by norleucine (Nle) and guided by reported SAR,^54,56^ replaced leucine (Leu) by cyclobutyl alanine (Cba) and glutamic acid (Glu) by glutamine (Gln). Given that the helical stretch distance (16.6 Å) is far larger than the distance between junction points in contiguously hybridized PNAs (4.5 Å), we included a glycine residue on both sides of the peptide (see Figure 3B for the graphical representation of distances). We thus used DTS to prepare two DNA tagged PNA-peptide conjugates, a potential binder (PNA- Gly Ser Nle **Cba** Gln *A*la **Trp** Tyr Glu Nle **Phe** Thr Leu Gly -PNA) and nonbinder (PNA- Gly Ser Nle **Ala** Gln *A*la **Ala** Tyr Glu Nle **Ala** Thr Leu Gly -PNA) with mutations in the residues that are critical for MDM2 interaction (shown in bold). To monitor the ligation reaction and distinguish these two synthetic products by gel electrophoresis, a longer DNA template was used for the nonbinder, and both compounds included a distinct fluorophore: Cy3 for the binder and Atto647N for the nonbinder (Figure 3C). A complete conversion to a new band was observed following hybridization and EDC treatment (Figure 3D). Following the EDC reaction, the full-length products were purified by gel extraction and combined in equimolar amounts prior to the selection. Affinity selection of this mixture against GST-MDM2 immobilized on GSH beads was monitored by fluorescence quantification of the supernatant solution. After four washes, heat elution was used to recover the selected mixture. Fluorescence analysis showed a strong recovery of the binder sequence but not of the nonbinder (Figure 3E). Taken together, the data confirm that the linker strategy used can accommodate an α-helical conformation of the peptide and that the DNA can be translated into the synthetic product via DTS for affinity selection.

**Figure 4:**
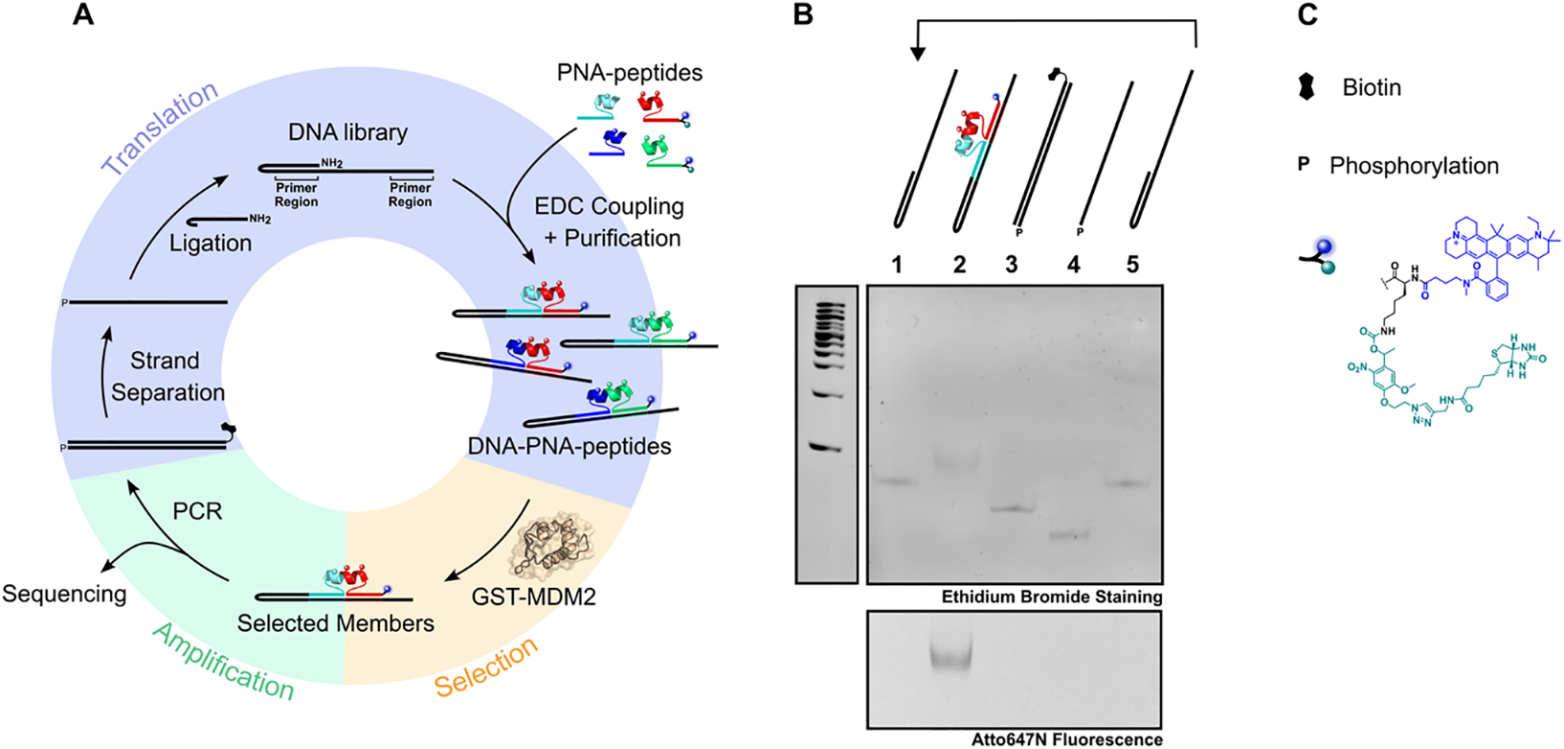
(A) Translation–selection–amplification cycle of PNA-displayed alpha helices. The hairpin amino DNA library is reacted with PNA-peptides. The conjugates are purified by streptavidin bead capture followed by photocleavage. The library is selected against GST-MDM2 and the selected members are PCR amplified (either for sequencing or for a second cycle). The double strand DNA is then retransformed into the hairpin amino DNA library via strand separation and hairpin ligation. (B) Gel analysis of different stages of the cycle described in (A). Native 15% PAGE, EtBr staining, and Atto647N fluorescence are shown. (C) Legend. Symbols present in (A) and (B) are described alongside the chemical structure of the photocleavable affinity tag.

In order to perform multiple cycles of translation-selection-amplification as required for Darwinian evolution, modifications of the system were necessary (Figure 4). Primer regions were added on either side of the coding region to amplify the selected codons. The PCR primers were designed to incorporate a 5′-phosphate and 5′-biotin into the PCR product, which was then converted to a 5′-phosphate single-stranded DNA via biotin capture.^31^ Hairpin ligation using T4 DNA ligase yielded the full-length DNA construct (94 bp) with a 5′ amino group, which was reacted with the synthetic PNA-peptide conjugates to regenerate the PNA-displayed α-helix library. This final step translates the genotype (DNA) into a phenotype (α-helical peptide) and is a core step in evolutionary technologies. Additionally, a fluorophore and photocleavable purification tag (biotin) were incorporated into the terminal PNA-peptides in order to visualize and purify the full-length constructs after the translation step^29,59^ (Figure 4C). The purification tag ensures that DNA that would not have been successfully translated is removed in the streptavidin capture step. Additionally, amino acids with side chains requiring a protecting group could be protected by using the same photolabile chemistry. The full cycle can be performed in just a couple of days and regenerates the same amount of material after each cycle (50 μL of a 7.5 μM solution of hairpin DNA).

**Figure 5:**
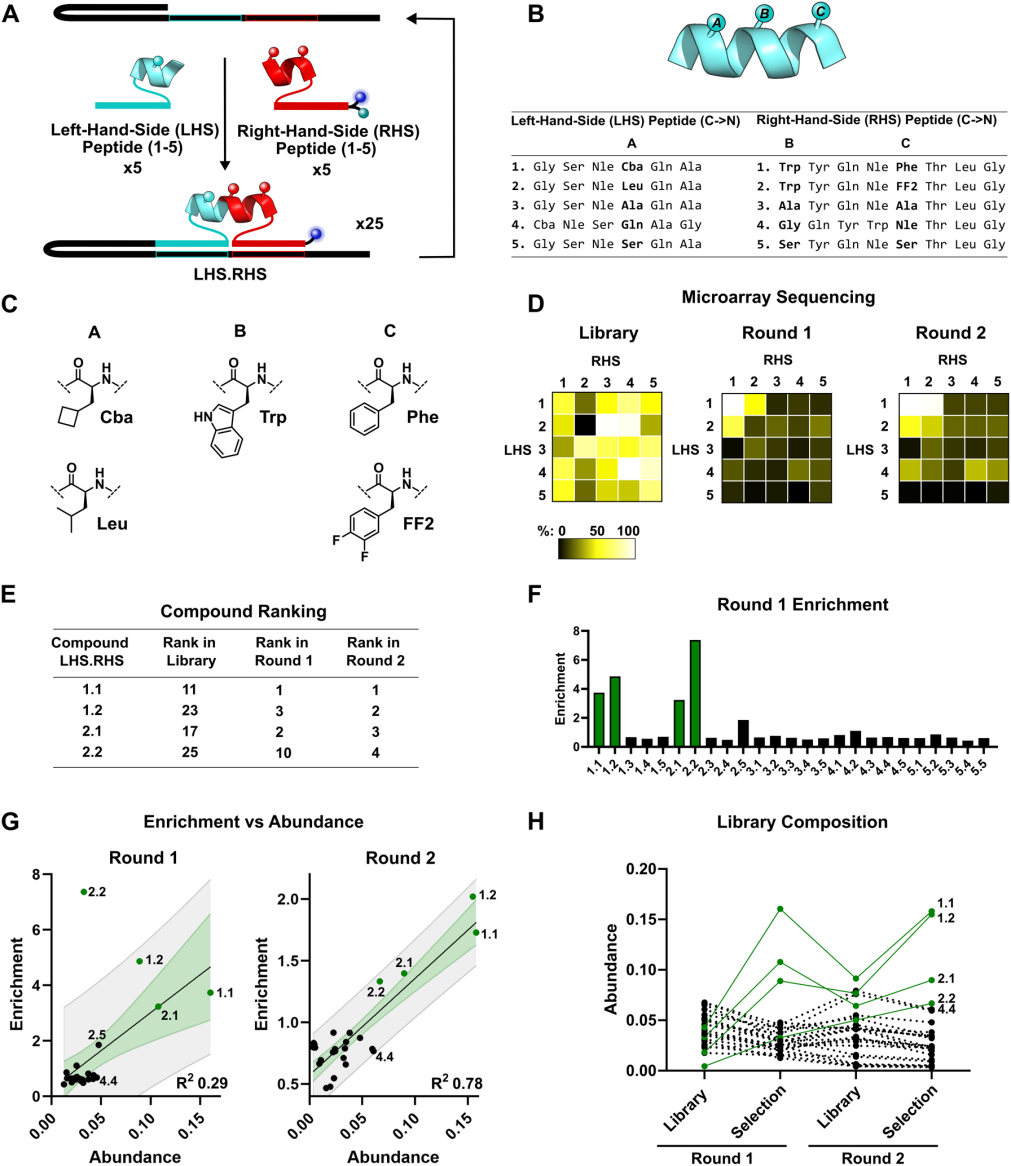
Selection of MDM2 binding sequences. (A) Schematic diagram representing the 5 × 5 library. (B) Sequences of library members. (C) Chemical structures of Cba, Leu, Trp, Phe, Leu, and FF2. (D) Microarray sequencing showing the library, selection round 1 and selection round 2. Each subsquare represents the fluorescence of a DNA sequence on the microarray, colored by intensity. (E) Ranking of hypothetical binders over multiple rounds of selection. (F) Enrichment levels of each member after the first selection round. (G) Enrichment versus abundance during round 1 (left) and round 2 (right). In both cases a linear regression (solid line) was plotted. The 95% confidence bands (green) and 95% prediction bands (gray) are shown. The hypothetical binders (**1.1**, **1.2**, **2.1**, **2.2**) are shown in green. (H) Changes in abundance of each library member across multiple selection rounds, with hypothetical binders (**1.1**, **1.2**, **2.1**, **2.2**) shown in green.

To investigate whether multiple cycles are beneficial in a selection, a 5 × 5 library was designed where five Left-Hand Side (**LHS**) fragments of the α-helix were combinatorially ligated with five Right-Hand Side (**RHS**) fragments encoded by 25 unique DNA strands. This templated reaction yielded 25 unique alpha helices (**LHS.RHS**). The library was designed to incorporate 2 binding peptides in the LHS and RHS fragments resulting in only four theoretical binders. When one binding fragment is combined with a nonbinding fragment, it will not display the triad of large hydrophobic residues and therefore should not interact with MDM2, resulting in 21 nonbinders (Figure 5A-C). The library was prepared via one-pot EDC coupling, with all DNAs and PNAs annealed prior to EDC addition. The reaction mixture was purified by biotin capture and photocleavage to yield a pure library. Optimization of the selection procedure afforded less noise if the protein capture on GST beads was performed after the incubation of MDM2-GST with the library. Using this optimized protocol, the library was subjected to a selection with GST-MDM2 or GST alone (control). The beads were washed to remove nonbinders and selected binders were recovered by heat denaturation. The DNA corresponding to selected members was then PCR amplified for microarray quantification (Figure 5D). Prior to the first selection, the library was relatively homogeneous with 24 out of 25 compounds falling within an abundance range of 0.02–0.07 (homogeneous abundance of a library member is 0.04, see Figure S3 for graphical distribution). The presumed binders, **1.1**, **1.2**, **2.1**, and **2.2**, were ranked 11th, 23rd, 17th, and 25th, respectively. After one round of selection, the four compounds were enriched roughly 4 to 8-fold compared to the starting library and ranked 1st, 3rd, 2nd, and 10th (Figure 5E,F). The selected binders were then subjected to a translation cycle which included PCR, strand separation, hairpin ligation, and coupling with PNA-peptides. The newly generated library was subjected to a second-round selection which followed similar trends to the first, resulting in **1.1**, **1.2**, **2.1**, and **2.2** being ranked first, second, third, and fourth, respectively (Figure 5E). Impressively library member **2.2** improved from the last position in the starting library to fourth after only two rounds of selection, showing that multiple rounds of selection are beneficial and are able to overcome heterogeneity in the starting library. This is specifically important as DEL selections are commonly analyzed by plotting enrichment versus sequence count.^60^ This technique enables the detection of false negatives, as well as the removal of statistical noise caused by low abundance members. Analyzing the 5 × 5 library using this technique after a single round of selection would result in **2.2** potentially being classed as a false positive (high enrichment but low abundance, Figure 5G). A second round of selection clearly removes this problem and yields a selection result with a clear correlation between the sequence count and enrichment. It is also worth noting that during the library regeneration cycle, heterogeneous reaction yields were observed (Figure 5H). The heterogeneity of yield could have multiple origins (e.g., PCR amplification variation, reaction performance variation, PNA-peptide concentration variation, etc.) but was especially observed with compounds featuring fragment **LHS4**. The peptide displayed a glycine at the reaction site, which we hypothesized could be favored in the reaction compared to more bulky residues.^61^ Library member **4.4**, where both the **LHS** and **RHS** fragments contain a glycine at the reaction sites was significantly amplified in the translation step over library members that required amide bond formation between two bulkier residues (ranked fifth after 2 rounds). These results offer a concrete example of a concern with reiterative translation/selection cycles. To exploit the full power of Darwinian evolution in chemical systems, it is imperative to decrease the chemical bias during the translation step. A simple consideration that can be included in the library design is to minimize the diversity of reactivity in the ligation step. It also highlights the importance of analyzing compound distribution prior to selection at each round. The synthetic bias resulting from the translation is clearly visible from the positive slope in the abundance before and after translation for library member **4.4**. This study shows that multiple selection cycles are beneficial only in the case that enrichment during selection is greater than homogenization during retranslation.

**Figure 6:**
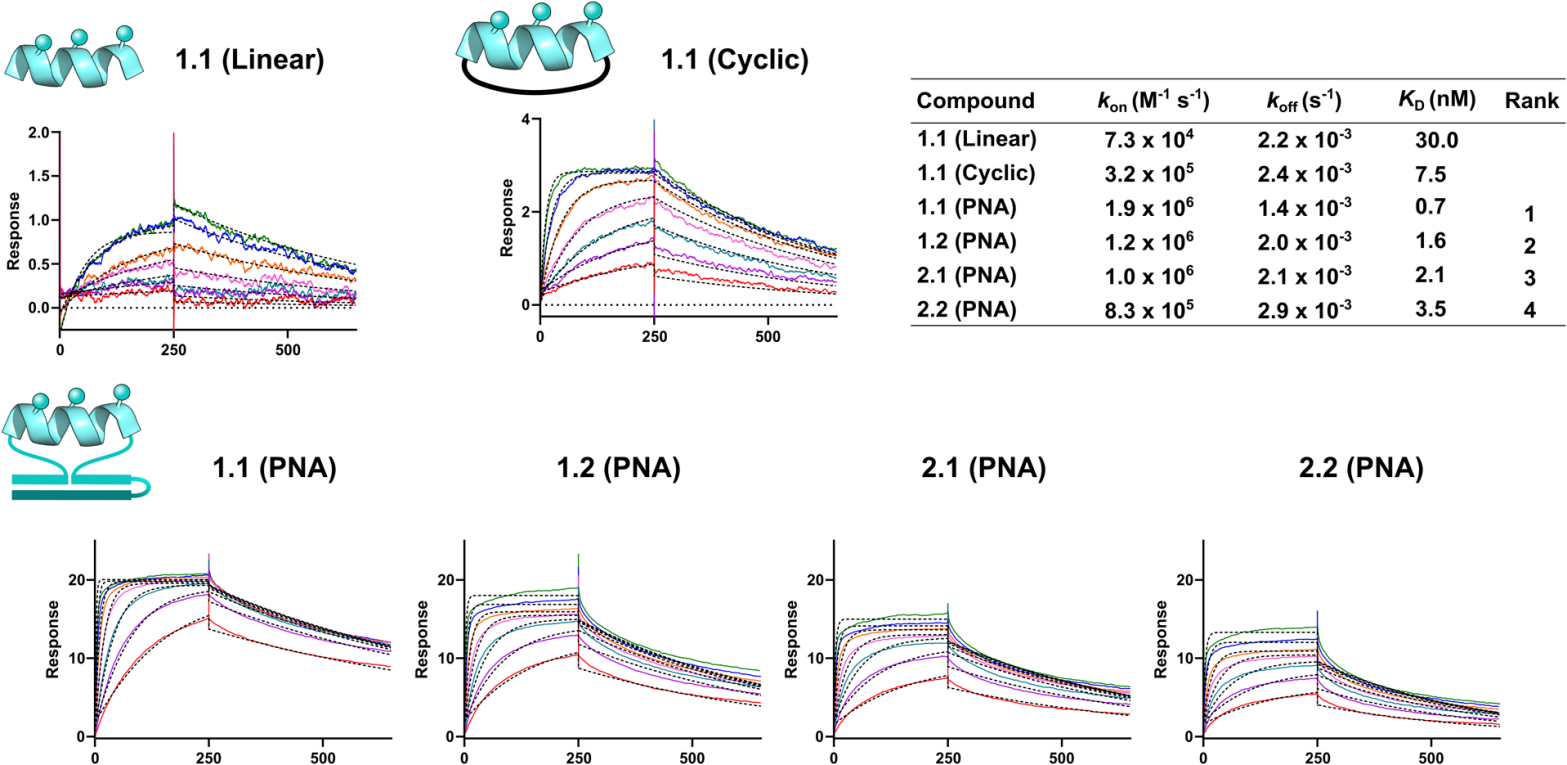
**Output of Library.** SPR curves of binders **1.1**, **1.2**, **2.1**, and **2.2** as PNA constrained peptides as well as **1.1** as a linear and cyclic peptide against MDM2.

To validate the ranking of the selection, binders **1.1**, **1.2**, **2.1**, and **2.2** and nonbinders **4.1** and **4.4** were synthesized as synthetic analogues mimicking the peptide head-to-tail hybridization. The analogues featuring only PNA and peptide were programmed to adopt a conformation similar to that of the DEL members. These analogues can be prepared via automated solid-phase peptide synthesis (SPPS). Surface plasmon resonance (SPR) validated the four binders with the K_D_ ranking confirming the selection ranking: **1.1** > **1.1** > **2.1** > **2.2** (Figure 6). The nonbinders showed no binding to MDM2 (Figures S4 and S5). As a comparison, the linear peptide **1.1** without PNA was synthesized and showed a 40-fold loss of binding; 30 versus 0.7 nM. A cyclic version of **1.1**, replacing the PNA head–tail stapling with a 27.5 Å linker, was also prepared and displayed 7.5 nM affinity, better than that of the linear peptide but not as good as that of the hybridization-constrained macrocycle. Interestingly, the *k*_off_ of the linear, cyclic, and PNA-constrained peptides did not vary significantly (1.4–2.4 × 10^–3^ s^–1^). The *k*_on_ was responsible for the difference in *K*_D,_ indicating that the PNA-constrained peptides had superior preorganization for binding than their linear or cyclic counterparts. Based on the requirement for an α-helical conformation in the binding to MDM2, these results support the hypothesis that the hybridization stabilizes the α-helical conformation of the peptide. The fact that the PNA-constrained peptide had faster association kinetics than the cyclic peptide suggests that further optimization in the head-to-tail cyclization is possible.^62^ Furthermore, the fact that the activity is modulated by a hybridization-driven folding suggests that this architecture lends itself to therapeutics with on-demand reversibility, using a competing oligonucleotide to disrupt the folding.^63^

## Conclusions

We have designed a DNA-encoded library architecture leveraging DTS^30^ for multiple cycles of translation-selection-amplification. This architecture was used to encode and constrain a biologically relevant α-helical conformation that binds to the oncoprotein MDM2. A pilot library was prepared to validate the selection process. Multiple rounds of selection were efficient in selecting and amplifying the lowest abundance binder. The study also highlights the importance of removing bias in translation chemistry to truly embrace the full power of Darwinian evolution. While the library size remains small, it offers a control setting to measure the benefits of reiterative cycles of selection and translation. Further work is currently ongoing to expand the library size, as well as the biological complexity. The chemistry presented herein employs only two coupling steps (“building blocks”), extending this chemistry to three or four coupling steps would facilitate the construction of larger libraries. Additionally, mini-proteins such as affibodies, affimers, and avimers, which exhibit antibody-like binding, are synthetically accessible and could be encoded and constrained via this technology. These scaffolds are currently discovered via biological libraries or *in silico* methods followed by biological expression limiting their chemical diversity to proteogenic amino acids. Finally, while synthetic peptides are typically stabilized into α-helical conformation by stapled side chains,^64^ this work demonstrates that head-to-tail cyclization is also effective and adds to the repertoire of cyclic peptides which enjoy renewed interest.^65^

## Methods

All reagents and solvents for the organic synthesis were purchased from commercial sources and were used without further purification. HPLC purification was performed with an Agilent Technologies 1260 infinity HPLC instrument using a ZORBAX 300SB-C18 column (9.4 × 250 mm). LC-MS spectra were recorded on a DIONEX Ultimate 3000 UHPLC with a Thermo LCQ fleet mass spectrometer system using a PINNACLE DB C18 column (1.9 μm, 50 mm × 2.1 mm) operated in positive mode. All the LC-MS spectra were measured by ESI. Unless specified otherwise, LCMS spectra were acquired over 4 min with an increasing gradient (5–90%) of acetonitrile 0.01% TFA in water 0.01% TFA. MALDI-TOF Mass spectra were measured by using a Bruker Daltonics Autoflex spectrometer operated in positive mode. High-resolution mass spectra (HRMS) were obtained on a Xevo G2 Tof spectrometer (Ionization mode: ESI positive polarity; mobile phase: MeOH 100 μL/min). Automated solid-phase synthesis was carried out on an Intavis AG Multipep RS instrument. Definitions of acronyms and physical characterization of reported compounds can be found in the Supporting Information. Raw data for the experiments is available from Zenodo.org: doi 10.5281/zenodo.13476570

### Solid Phase Synthesis of Compounds

A 5.0 mg amount of resin was swollen in DCM for 10 min and washed twice with DMF. Iterative cycles of amide coupling (procedure 1), capping of the resin (procedure 4), and deprotection of the protecting group (procedure 2 or 3) were done to synthesize the PNA probes. The compounds were deprotected and cleaved from the resin using procedure 5 and finally purified by using HPLC.

#### 2-Chlorotrityl Chloride Resin Loading

2-Chlorotrityl chloride resin (Novabiochem #8.55017) (1.46 mmol/g loading) was swollen in dry DCM for 30 min, followed by washing with DCM+1% DIPEA (1 × 3 mL) and DCM (10 × 3 mL). A solution of Fmoc-Xaa–OH (0.7 mmol/g resin) and DIPEA (4 equiv relative to resin functionalization) in DCM (final concentration 0.125 M of amino acid) was added to the resin, which was shaken at room temperature for 16 h. The resin was then washed with DCM (5 × 3 mL), DMF (5 × 3 mL) and DCM (5 × 3 mL). The resin was then capped via treatment with 17:2:1 v/v/v DCM:MeOH:DIPEA (5 mL) for 40 min at room temperature. The resin was then washed again with DCM (5 × 3 mL), DMF (5 × 3 mL) and DCM (5 × 3 mL) prior to further use. For PNA strands on 2-chlorotrityl resin, glycine is added as the first monomer to avoid cyclization and therefore resin cleavage during Fmoc deprotection.

#### Rink Amide Resin Loading

Fmoc-Rink Amide PEG AM Resin (0.33 mmol/g, Iris Biotech, BR-1360) was swollen in DCM for 10 min and washed twice with DMF. The resin was Fmoc deprotected (procedure 2) and standard amide coupling (procedure 1) was performed, followed by capping of the resin (procedure 4). The resin was then washed again with DCM (5x 3 mL), DMF (5 × 3 mL), and DCM (5x 3 mL) prior to further use.

##### Procedure 1 (P1): Amide Coupling

The corresponding Fmoc-protected PNA monomer or amino acid (4.0 equiv, 0.2 M in NMP) was incubated for 5 min with HATU (3.5 equiv, 0.5 M in NMP) and base solution [DIPEA, 1.2 M (4.0 equiv) and 2,6-lutidine 1.8 M (6.0 equiv) in NMP]. The mixture was then added to the corresponding resin. After 20 min, the mixture was filtered, the resin was washed with DMF, and a new premixed reaction solution was added to the resin and allowed to react for another 20 min. Finally, the resin was washed with 2× DMF, 2× DCM, and 2× DMF.

##### Procedure 2 (P2): Fmoc Deprotection

A solution of 20% (v/v) piperidine in DMF was added to the resin and allowed to react for 5 min. The mixture was then filtered, the resin washed with DMF, and the sequence repeated for another 5 min. Finally, the resin was washed with 2× DMF, 2× DCM, and 2× DMF.

##### Procedure 3 (P3): Mtt Deprotection

A solution (made from 244 mg of HOBt in 10 mL of HFIP and 10 mL of DCE) was added to the prewashed resin to reach a volume of 10 mL/g of resin and allowed to react for 5 min. The solution was flushed, the resin washed with DCM, and the sequence repeated for another 5 min. Finally, the resin was washed with 2× DCM and 2× DMF.

##### Procedure 4 (P4): Capping

The resin was treated with a capping mixture (0.92 mL of acetic anhydride and 1.3 mL of 2,6-lutidine in 18 mL of DMF: 10 mL of solution/g of resin) for 5 min. After the solution was flushed, the resin was washed with 2× DMF, 2× DCM, and 2× DMF.

##### Procedure 5 (P5): Cleavage from the Resin and Final Deprotection

Resin (5.0 mg, 1.0 μmol) was treated with 300 μL of TFA for 2 h. The resin was filtered and washed with TFA (50 μL), and the collected fractions of cleavage product precipitated in cold ether (1.5 mL). After centrifugation, the pellet was vortexed again with cold Et_2_O (1.5 mL) and centrifuged (14k rpm). The pellet was dissolved in H_2_O/CH_3_CN (3:1, 1.5 mL) and lyophilized to obtain a white powder. In the case of compounds containing the photolinker-biotin purification tag, the resin was treated with 300 μL of TFA with scavengers: 96.5% TFA, 2% v/v Me_2_S, and 1.5% w/v NH_4_I.

##### Procedure 6 (P6): Microcleavage for Quality Control

The minimum number of beads was picked up with a pipet plastic tip and transferred to 50 μL of TFA. The solution was left for 1 h and transferred to 1.0 mL of ether. The ether solution was kept for 5 min at −20 °C and then centrifuged for 5 min at 14k rpm. The ether supernatant was removed, and the pellet was dissolved in 20 μL of 1:1 acetonitrile/water, which was then used to analyze by MALDI and/or LC-MS.

##### Procedure 7 (P7): On-Resin Cyclization

The corresponding resin (5 mg) was Fmoc-deprotected (P2) and a mixture of diglycolic anhydride (2 equiv, 100 μL of NMP) and DIPEA (3 equiv) were added. After 30 min, the mixture was filtered, the resin was washed with 2× DMF and 2× DCM, and the reaction was checked by microcleavage (P6). The resin was then Mtt deprotected (P3) and HATU (1.5 equiv) and DIPEA (3 equiv) were added to the resin. After 2 h, the mixture was filtered, and the resin was washed with 2× DMF and 2× DCM. It is worth noting that dicyclic peptides were also observed under these conditions but could easily be removed during purification, and no further optimization was investigated*.*

##### Procedure 8 (P8): Photolinker Coupling

Fmoc-Lys(PL-Biotin)–OH (synthesis described below) (2.0 equiv, in 50 μL of NMP) was incubated for 5 min with HATU (1.5 equiv, in 50 μL of NMP) and DIPEA (6 equiv). The mixture was then added to the corresponding resin. After 2 h, the mixture was filtered, and the resin was washed with 2× DMF, 2× DCM, and 2× DMF.

### Characterization of PNA-Peptide Conjugates

Characterization of the PNA-peptide conjugates was done by MALDI (Bruker Daltonics Autoflex spectrometer with Flex control 3.4 software and analysis with FlexAnalysis 3.4) and/or LC-MS (DIONEX Ultimate 3000 UHPLC with a Thermo LCQ Fleet Mass Spectrometer System using a PINNACLE DB C18 column (1.9 μm, 50 mm × 2.1 mm) with Thermo Xcalibur 2.2.SP1.48 software and analysis with Thermo Xcalibur Qual Browser 2.2.Sp1.48). For MALDI analysis, 1.0 μL of the sample (in either water or water/acetonitrile 1:1) was mixed with 1.0 μL of DHB matrix solution (30 mg of DHB in 1.0 mL of 70:30:0.01 water/acetonitrile/TFA), and the mixture was spotted on a MALDI plate. The measurements were taken in positive linear mode. For LC-MS analysis, 20 μL of sample in water or water/acetonitrile 1:1 was injected into the LC and further analyzed by MS in positive mode.

### MDM2 Selection Procedures

MDM2 protein was prepared according to previously reported methods.^44^

#### Binder/Nonbinder Selection with Plate Reader Readout

Ten μL of MagneGST glutathione particles (Promega #V8611) were blocked with 1 mg/mL BSA in PBS-CHAPS (10 mM phosphate buffer, 2.7 mM KCl, and 137 mM NaCl, pH 7.4, with 0.05% CHAPS) three times at room temperature for 5 min. Subsequently, 50 μL of GST-MDM2 (2.2 μM) in PBS-CHAPS was captured on the above-blocked beads for 30 min at room temperature. Beads were then washed with a solution of 1 mg/mL BSA in PBS-CHAPS three times on ice. A 1:1 mixture of binder and nonbinder (500 nM each, 100 μL) in PBS-CHAPS was incubated with captured protein for 2 h at 4 °C on a revolving shaker. The magnetic beads were washed 4 times with 100 μL of PBS-CHAPS containing 1 mg/mL BSA for 5 min at room temperature. To elute the binders, 100 μL of PBS-CHAPS containing 1 mg/mL BSA was added, and the beads were heated to 95 °C for 5 min. The initial library, post-incubation library, washes, and elution were analyzed by a plate reader (SpectraMax i3x, in black 96-well microtiter plates (Thermo Scientific #267342)). The fluorescence of the binder (Ex 530 nm, Em 565 nm) and nonbinder (Ex 646 nm, Em 664 nm) were monitored, and the washes were normalized to the maximum amount bound (fluorescence of library preincubation–fluorescence of library postincubation).

#### Library Selection

Library members (10 μL, 15 nM) were incubated with either GST or GST-MDM2 (10 μL, 1 μM) for 1 h at room temperature. The mixture was then added to prewashed (3 × 100 μL wash buffer) MagneGST Glutathione Particles (Promega #V8611) and put on a revolving shaker for 1 h at room temperature. The magnetic beads were washed 3 times with 100 μL of wash buffer, followed by 1 time with 100 μL of PBS-CHAPS. To elute the binders, 10 μL of PBS-CHAPS was added, and the beads were heated to 95 °C for 5 min. The supernatant was recovered and PCR amplified for microarray sequencing.

Wash buffer: PBS-CHAPS (10 mM phosphate buffer, 2.7 mM KCl and 137 mM NaCl, pH 7.4, with 0.05% CHAPS) + 0.05% salmon sperm DNA (Sigma-Aldrich #D9156–5 ML) + 1 mg/mL BSA (Sigma-Aldrich #A3294–50G).

### Microarray Quantification

#### PCR of DNA

PCR reactions were performed in 8-tube strips (BRAND #781320) using standard conditions and reagents (see Supplementary Information, section 2). In each well (50 μL final volume), 1 μL of either elution from MDM2 selection, GST selection, or 2% library were added. Three × 50 μL reactions were performed for each DNA template. Twenty-three cycles were performed. The three reactions were combined and purified by a Qiagen QIAquick PCR Purification Kit (Qiagen #28106).

Primers used were primer 3 (FP) and primer 4 (RP); see the Table S11 for sequences.

#### Double-Stranded DNA to Single-Stranded DNA

40 μL Dynabeads MyOne Streptavidin C1 (Thermo Scientific #65001) were washed with binding and washing (B&W) buffer (1×, 1 mL). Double strand DNA (20 μL) and B&W buffer (2×, 20 μL) were added and incubated for 20 min at room temperature. The beads were washed once with B&W buffer (1×, 1 mL). The single-stranded DNA was eluted by the addition of NaOH (0.1 M, 10 μL) for 15 min at room temperature. The solution was removed and HCl (0.2 M, 5 μL) and Tris-HCl (1 M pH 8.0, 0.5 μL) were added. The single-stranded DNA was quantified by a nanodrop (A_260 nm_).

B&W Buffer 10×: 10 mM Tris-HCl (pH 7.5), 1 mM EDTA, 2 M NaCl.

#### Microarray Hybridization

Triton-X 10% (200 μL), 2× hybridization buffer (200 μL), and 10 mg/mL stock salmon sperm DNA (4 μL) were vortexed together (hybridization mixture). For each DNA sample to be sequenced (library, MDM2 selection, GST selection), 1 μL of 10 nM single-stranded DNA (Cy3 labeled, quantified by nanodrop A_260 nm_) was added to 99 μL of the hybridization mixture. The mixture was heated to 95 °C for 5 min and left to cool. 90 μL of each solution was pipetted onto a microarray gasket placed in a hybridization chamber. The complementary custom-made DNA microarray slide from Agilent (Agilent design: 740191) was slowly placed on top, and the metal chamber was tightened and gently tapped on the bench in order to remove bubbles. The samples were incubated in a rotating chamber for 4 h at 60 °C. The slide was removed from the incubator and gently separated from the gaskets using forceps. The slide was washed in 300 mL of 2× SSC buffer containing 0.1% SDS for 5 min, followed by 300 mL of 0.2× SSC buffer containing 0.1% SDS for 5 min, and finally briefly rinsed in 300 mL of Milli-Q water. The slide was then centrifuged at 1000 rpm for 1 min and scanned at 532 nm with the GenepixPro 7.1 software. The fluorescence intensity of each member is the mean value of 23 different spots corresponding to the same DNA sequence in the microarray.

Hybridization buffer 2×, 2.4 M lithium chloride; 0.6 M Li-MES; 0.024 M EDTA; 6% Li-DS.

Saline solution citrate 20×, 3.0 M sodium chloride, 0.3 M sodium citrate, pH 7.0.

Each DNA sequence is present on the Agilent slides 23 times. The mean fluorescence of the pixels of each spot is computed, and the median of the 23 means is calculated and shown in the table below. The results are formatted in Excel with a 3-color scale, minimum (0%): black, midpoint (50%): yellow, maximum (100%): white. The abundance of each member is calculated by its fluorescence intensity/total fluorescence intensity. The enrichment is calculated by an abundance selection/abundance library.

The enrichment versus abundance analysis shown in Figure 5 consists of the abundance of each compound plotted versus the enrichment in rounds 1 and 2 of the selection. The hypothetical binders **1.1**, **1.2**, **2.1**, and **2.2** are shown in green. A standard linear regression was plotted by using GraphPad 10.2.0, and the 95% confidence band and prediction bands were displayed.

### Surface Plasmon Resonance

SPR experiments were performed on a Biacore T200 instrument (GE Healthcare) at 25 °C in PBS-P+ buffer (10× stock; Cytiva Life Sciences #28995084). One mL PP 96-well plates (Greiner #780201) were used for dilution directly in the plates. Anti-GST antibody (Cytiva #27457701) was chemically immobilized on a CM5 series S sensor chip (Cytiva Life Sciences #29149604) by EDC/NHS-mediated coupling. For this, an amine coupling kit (Cytiva Life Sciences #BR100633) and the recommended protocol were followed with the following specifications: 20 μg/mL of antibody diluted in 10 mM NaAc pH 5.0 buffer flowed over both flow channels for 600 s (flow rate 10 μL min^–1^).

Kinetic measurements consisted of ligand capture of GST-MDM2 (200 nM, 350 s injection, flow rate 10 μL min^–1^, stabilization 60 s), followed by injections (association, 250 s; dissociation, 400 s; flow rate, 30 μL min^–1^) of decreasing concentrations of compounds (2-fold cascade dilutions from the starting concentration). The chip was regenerated between cycles by one injection of regeneration solution (10 mM glycine-HCl at pH 1.5) for 10 s at a flow rate of 30 μL min^–1^, followed by a 120 s stabilization period. Binding was measured as resonance units over time after blank subtraction, and the data were interpreted using Biacore T200 software (version 3.2). The *K*_D_ values were calculated based on steady-state affinity (1:1 binding).

## Supporting Information Available

Physical characterization of compounds used in the studies with explicated structures; Figures S1–S6 (PDF)
