# Supplementary material for: Translation of Deoxyribonucleic Acid into Synthetic Alpha Helical Peptides for Darwinian Evolution
Source: JACS Au. 2024 Oct 2;4(10):4013–22. doi: 10.1021/jacsau.4c00738 (PMC11522901; doi:10.1021/jacsau.4c00738)
Supplement: Supplementary file 1 — au4c00738_si_001.pdf [file au4c00738_si_001.pdf]

Supplementary Information

Translation of Deoxyribonucleic Acid into Synthetic Alpha Helical Peptides for Darwinian Evolution

Millicent Dockerill[a], Pramod M. Sabale[a], Francesco Russo[a], Sofia Barluenga[a], Nicolas Winssinger[a*]

[a]Department of Organic Chemistry, Faculty of Sciences, University of Geneva, 1211 Geneva, Switzerland

*nicolas.winssinger@unige.ch

Table of Contents

1. Acronyms ... 3
2. DNA methods ... 4
3. DNA-PNA methods ... 6
4. MDM2 Selection Results ... 9
5. Surface Plasmon Resonance Results ... 9
6. DNA Sequences ... 10
7. MDM2 Crystal Structure Analysis ... 12
8. Synthesis of Purification Tag ... 12
9. Characterisation of PNA, PNA-peptide and peptide compounds ... 22
10. Full gels from figures ... 47
11. References ... 50

1. Acronyms

CuAAC	Copper(I)-catalyzed azide-alkyne cycloaddition
DAP	Diaminopropionic acid
DCE	1,2-dichloroethane
DCM	Dichloromethane
DHB	2,5-Dihydroxybenzoic acid
DIPEA	N,N-Diisopropylethylamine
DMF	Dimethylformamide
DMSO	Dimethyl sulfoxide
EDC	1-Ethyl-3-(3-dimethylaminopropyl)carbodiimide
ESI	Electrospray ionization
Fmoc	Fluorenylmethoxycarbonyl
HATU	Hexafluorophosphate Azabenzotriazole Tetramethyl Uronium
HIFP	Hexafluoroisopropanol
HOBt	Hydroxybenzotriazole
HPLC	High performance liquid chromatography
HRMS	High-resolution mass spectra
LCMS	Liquid chromatography-mass spectrometry
MALDI	Matrix-assisted laser desorption/ionization
MS	Mass spectrometry
NaAsc	Sodium ascorbate
NMP	N-Methyl-2-pyrrolidone
PEG	Polyethylene glycol
PNA	Peptide Nucleic Acid
TBTA	Tris((1-benzyl-4-triazolyl)methyl)amine
TFA	Trifluoroacetic acid
UHPLC	Ultra High performance liquid chromatography

2. DNA methods

PCR Amplification Reaction

Supplementary Table 1. Standard PCR Amplification Reaction Conditions

Standard PCR Amplification Reaction Conditions			
**Step**	**Temperature (°C)**	**Duration (s)**	**Cycles**
Step 1: Initial Denaturation	95	60	
Step 2: Denaturation	95	30	Repeat
Step 3: Annealing	62	30	
Step 4: Extension	72	60	
Step 5: Hold	4	Hold	Hold

Supplementary Table 2. Standard PCR Amplification Reaction Reagents

Standard PCR Amplification Reaction (50 µL) Reagents			
**Reagent**	**Stock Concentration**	**Volume (µL)**	**Final Concentration**
HF Buffer (Thermo Scientific #F-530XL)	10X	10	1X
dNTPs (R0192)	10 mM	1	200 µM
Forward Primer	100 µM	0.5	1 µM
Reverse Primer	100 µM	0.5	1 µM
Phusion (Thermo Scientific #F-530XL)	2 U/µL	0.5	0.02 U/µL
Water	NA	36.5	NA
DNA Template	NA	1	NA

- PCR for first round library generation

25 individual non hairpin DNA strands were purchased - full coding sequences shown in table of DNA sequences in section 6): GTCTATTGCAGGCAAGCTC XXX XXXX XXX YYY YYYY YYY CGAGTCATAGCGCATCGTT

PCR reactions were performed in a 96-well plate (Thermo Scientific #AB-0600). In each well (50 µL final volume), 0.5 µL of individual commercial single stranded DNA (100 nM) was added (final concentration 1 nM). 20 cycles were performed following standard conditions. For each DNA (25 different sequences), 4 x 50 µL reactions were performed and combined. NaAc (20 µL, 3 M) and EtOH (1 mL) was added and left at -80 °C overnight. The DNA was centrifuged (30 minutes, - 10 °C, 10 000 rpm) and the supernatant was removed. The dried precipitated DNA was then dissolved in water (125 µL) and purified using a Qiagen QIAquick PCR Purification Kit (Qiagen #28106). The individual purified DNA strands were quantified by nanodrop (A_{260nm}) and combined to obtain 10 pmol of each strand (total DNA 250 pmol, 1.1 µM). The combined library then underwent standard procedures (double strand to single strand conversion and hairpin ligation) to obtain a library of 25 hairpin DNA (5' amino).

Primers used: Primer 1 (FP), Primer 2 (RP) – see table for sequences.

- PCR for cycle regeneration

PCR reactions were performed in a 96-well plate (Thermo Scientific #AB-0600). In each well (50 µL final volume), 1 µL of single stranded DNA used for microarray sequencing (5 nM) was added (final

concentration 0.1 nM). 14 cycles were performed following standard conditions. The reactions were combined, NaAc (500 µL, 3 M) and EtOH (20 mL) was added and left at -80 °C overnight. The DNA was centrifuged (30 minutes, - 10 °C, 10 000 rpm) and the supernatant was removed. The dried precipitated DNA was then dissolved in water (600 µL) and purified using a Qiagen QIAquick PCR Purification Kit (Qiagen #28106).

Primers used: Primer 1 (FP), Primer 2 (RP) – see table 11 for sequences.

- PCR post selection

See methods section (MDM2 selection procedure) for more details.

Double strand to single strand conversion (via biotin capture)

500 µL Dynabeads MyOne Streptavidin C1 (Thermo Scientific #65001) were washed with binding and washing (B&W) buffer (1X, 1 mL). Double strand DNA (185 µL, 1.1 µM) and B&W buffer (2X, 185 µL) were added and incubated for 15 minutes at room temperature. The beads were washed twice with B&W buffer (1X, 1 mL). The single stranded DNA was eluted by addition of NaOH (0.1 M, 100 µL) for 15 minutes at room temperature. The solution was removed and HCl (0.2 M, 50 µL) was added. The single stranded DNA was quantified by nanodrop (A_{260nm}) prior to further use.

B&W Buffer 10X: 10 mM Tris-HCl (pH 7.5), 1 mM EDTA, 2 M NaCl

Hairpin Ligation

T4 DNA ligase was added to a mixture of single stranded DNA, DNA hairpin in T4 DNA Ligase Buffer (see table below for conditions). The mixture was shaken for 2 hours at room temperature. The enzyme was inactivated by heat denaturation at 60 °C for 20 minutes. 220 µL of phenol/chloroform/isoamyl alcohol (Roti® #A156.2) was added, the mixture was vortexed and centrifuged. The upper aqueous layer was transferred to a new Eppendorph. 220 µL of chloroform was added, the mixture was vortexed and centrifuged. The upper aqueous layer was transferred to a new Eppendorph. The reactions were combined, NaAc (20 µL, 3 M) and EtOH (1 mL) was added and left at -80 °C overnight. The DNA was centrifuged (30 minutes, - 10 °C, 10 000 rpm) and the supernatant was removed. The dried precipitated DNA was then dissolved in water (400 µL) and subjected to size exclusion spin filter (Amicon® Ultra Centrifugal Filter, 3 kDa MWCO, UFC5003). The centrifugal filter was spun for 15 minutes at 13 000 rpm at room temperature. The retained solution was collected and quantified by qPCR (details below). The hairpin DNA was used without further purification.

Supplementary Table 3. Standard Hairpin Ligation Reagents

**Standard Hairpin Ligation Reagents (220 µL)**			
**Reagent**	**Stock Concentration**	**Volume (µL)**	**Final Concentration**
Single stranded DNA (5' Phos)	Variable	Variable	1 µM
DNA Hairpin (5' Amino)	100 µM	2.65	1.2 µM
T4 DNA Buffer (NEB #M0202S)	10X	22	1X
T4 DNA Ligase (NEB #M0202S)	400 000 U/mL	5	2000 U/mL
Water	NA	Up to 220	NA

qPCR

Supplementary Table 4. Standard qPCR Amplification Reaction Conditions

Standard qPCR Amplification Reaction Conditions			
**Step**	**Temperature (°C)**	**Duration (s)**	**Cycles**
Step 1: Initial Denaturation	95	60	
Step 2: Denaturation	95	30	Repeat
Step 3: Annealing		30	
Step 4: Extension		60	
Step 5: Hold	4	Hold	Hold

Supplementary Table 5. Standard qPCR Amplification Reaction Reagents

Standard PCR Amplification Reaction (10 µL) Reagents			
**Reagent**	**Stock Concentration**	**Volume (µL)**	**Final Concentration**
SYBR® Green Supermix (Bio Rad #1725271)	2X	5	1X
Forward Primer	10 µM	1	1 µM
Reverse Primer	10 µM	1	1 µM
Water	NA	2	NA
DNA Template	NA	1	NA

qPCR reactions were performed using a CFX Connect Real-Time PCR Detection System in a 96-well plate (Bio-Rad #HSP9665) and sealed with clear adhesive film (Azenta #4TI-0500). 30 cycles were performed, and fluorescence was measured at the end of each extension step.

Primers used: Primer 1 (FP), Primer 2 (RP) – see table for sequences.

A standard curve was made using a commercial stock of DNA 1.1 (see figure below)

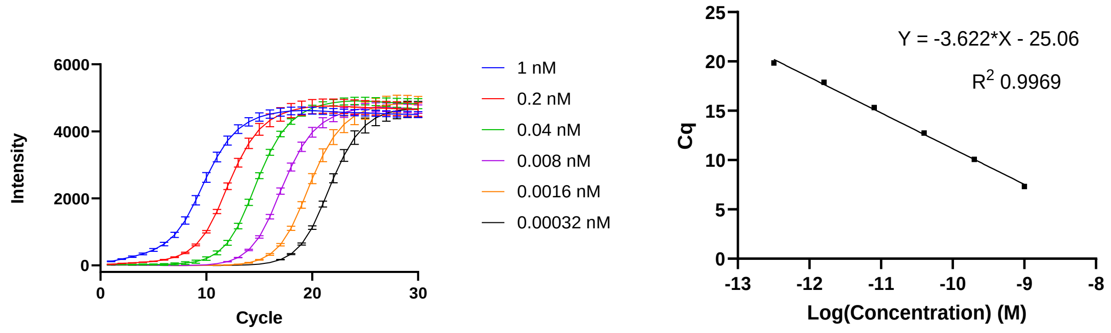

Supplementary Figure 1. qPCR Standard Curve of Hairpin DNA (Commercial)

3. DNA-PNA methods

DNA-PNA EDC reaction (Initial Validation)

All reagents were added (except EDC), mixed and heated to 95 °C for 5 minutes. The mixture was cooled to room temperature and deposited on ice for 15 minutes. EDC was added and the reaction was shaken at room temperature overnight. The reactions were analysed by denaturing (8M UREA) 15 % PAGE – see section 10.

Supplementary Table 6. DNA-PNA EDC reaction conditions Initial Validation

DNA-PNA EDC reaction conditions (100 µL) Initial Validation			
**Reagent**	**Stock Concentration**	**Volume (µL)**	**Final Concentration**
Hairpin DNA (5' Amino)	100 µM	1	1 µM
Each PNA (1-4)	100 µM	1.2	1.2 µM
DMF	NA	10	10 %
MES Buffer (500 mM MES, 100 mM pyridine, pH 4.5)	5X	20	1X
NaCl	1 M	10	0.1 M
Water	NA	Up to 100	NA
EDC	0.5 M	10	0.05 M

DNA-PNA EDC reaction (varying EDC concentration)

All reagents were added (except EDC), mixed and heated to 95 °C for 5 minutes. The mixture was cooled to room temperature and deposited on ice for 15 minutes. Different amounts of EDC were added and the reaction was shaken at room temperature overnight. The reactions were analysed by denaturing (8M UREA) 15 % PAGE.

Supplementary Table 7. DNA-PNA EDC reaction conditions Initial Validation

DNA-PNA EDC reaction conditions (100 µL) Initial Validation			
**Reagent**	**Stock Concentration**	**Volume (µL)**	**Final Concentration**
Hairpin DNA (5' Amino)	100 µM	1	1 µM
Each PNA (1-4)	100 µM	1.2	1.2 µM
DMF	NA	10	10 %
MES Buffer (500 mM MES, 100 mM pyridine, pH 4.5)	5X	20	1X
NaCl	1 M	10	0.1 M
Water	NA	Up to 100	NA
EDC	0.5 M	X	X

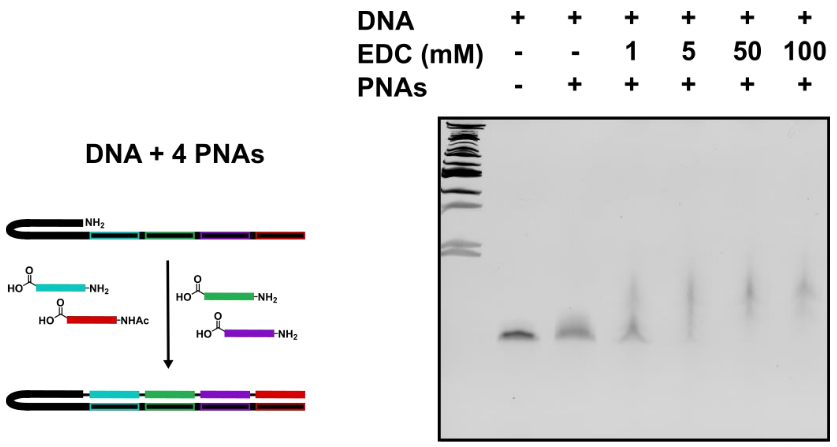

Supplementary Figure 2. DNA-PNA reaction with varying concentrations of EDC (1mM, 5 mM, 50 mM, 100 mM)

DNA-PNA EDC reaction (scale-up of binder and non-binder)

All reagents were added (except EDC), mixed and heated to 95 °C for 5 minutes. The mixture was cooled to room temperature and deposited on ice for 15 minutes. EDC was added and the reaction was shaken at room temperature overnight. The reactions were desalted using Sep-Pak C18 Classic Cartridge (Waters #WAT051910). After desalting, the product was eluted with 60% acetonitrile and lyophilised. The lyophilizate was dissolved in loading buffer (8M UREA, TBE), purified by denaturing (8M UREA) 15% Page: The band (observed by UV) corresponding to the DNA-PNA adduct was cut, crushed, suspended in water (2 mL) and rotated overnight. The suspension was filtered, lyophilised, redissolved in water (50 µL) and quantified by nanodrop (A_{550nm} for binder and A_{646nm} for non-binder).

Supplementary Table 8. DNA-PNA EDC reaction conditions Scale-up

**DNA-PNA EDC reaction conditions (2000 µL) Scale-up**			
**Reagent**	**Stock Concentration**	**Volume (µL)**	**Final Concentration**
Hairpin DNA (5’ Amino)	100 µM	200	10 µM
LHS PNA	100 µM	240	12 µM
RHS PNA	100 µM	240	12 µM
MES Buffer (500 mM MES, 100 mM pyridine, pH 5.9)	5X	400	1X
NaCl	1 M	200	0.1 M
Water	NA	520	NA
EDC	0.5 M	200	0.05 M

DNA-PNA EDC reaction (library) + bead purification

All reagents were added (except EDC), mixed and heated to 95 °C for 5 minutes. The mixture was cooled to room temperature and deposited on ice for 15 minutes. EDC was added and the reaction was shaken at room temperature overnight.

250 µL Pierce™ High Capacity Streptavidin Agarose Resin (Thermo Scientific #20359) were washed with PBS (1X, 1 mL). Crude reaction (110 µL) and PBS buffer (2X, 110 µL) were added and incubated for 1 hour at room temperature. The beads were washed with PBS (1X, 250 µL), NaOH (0.1 M, 250 µL), and twice with PBS (1X, 500 µL). PBS (1X, 40 µL) was adding and the suspension was irradiated with 365 nm light for 15 minutes. The photocleavage was repeated with PBS (1X, 40 µL) a second time. The two cleavage solutions were combined and quantified by qPCR (using DNA standard curve above).

Supplementary Table 9. DNA-PNA EDC reaction conditions Library

**DNA-PNA EDC reaction conditions (120 µL) Library**			
**Reagent**	**Stock Concentration**	**Volume (µL)**	**Final Concentration**
Hairpin DNA library (5’ Amino)	7.0 µM	43	2.5 µM
LHS PNA library	100 µM	7.2	6 µM
RHS PNA library	100 µM	7.2	6 µM
DMF	NA	12	10 %
MES Buffer (500 mM MES, 100 mM pyridine, pH 4.5)	5X	24	1X
NaCl	1 M	12	0.1 M
Water	NA	2.6	NA
EDC	0.5 M	12	0.05 M

4. MDM2 Selection Results

Full screening results (Full microarray values + GST counter screen)

Supplementary Table 10. Microarray Raw Values

Initial Library – Round1

	RHS1	RHS2	RHS3	RHS4	RHS5
LHS1	2507	1068	2167	3037	2139
LHS2	1945	260	3928	3823	1504
LHS3	1413	3237	2699	2359	2926
LHS4	2662	1335	2047	3955	3555
LHS5	2345	1029	1821	1698	3004

Initial Library – Round 2

	RHS1	RHS2	RHS3	RHS4	RHS5
LHS1	4777	4006	1612	1652	1295
LHS2	3352	2614	2717	2371	2148
LHS3	730	2219	2153	1761	2212
LHS4	3966	2167	1530	4135	2864
LHS5	342	252	298	226	814

MDM2 Selection – Round1

	RHS1	RHS2	RHS3	RHS4	RHS5
LHS1	7648	4240	1181	1400	1227
LHS2	5139	1564	2006	1528	2276
LHS3	757	2024	1378	975	1404
LHS4	1775	1208	1082	2181	1779
LHS5	1162	726	954	600	1483

MDM2 Selection – Round 2

	RHS1	RHS2	RHS3	RHS4	RHS5
LHS1	6863	6723	1049	1053	986
LHS2	3893	2894	1489	1400	1501
LHS3	405	1455	855	684	1005
LHS4	2583	1650	964	2635	2082
LHS5	226	175	203	150	469

GST Selection – Round1

	RHS1	RHS2	RHS3	RHS4	RHS5
LHS1	813	464	858	1144	937
LHS2	749	112	1271	1211	1341
LHS3	410	1185	951	770	912
LHS4	1094	647	698	1613	1235
LHS5	727	409	739	513	1044

GST Selection – Round 2

	RHS1	RHS2	RHS3	RHS4	RHS5
LHS1	3091	3162	853	1097	971
LHS2	2182	1799	1555	1736	1801
LHS3	350	1382	867	802.5	1078
LHS4	3378	2627	1324	4352	2701
LHS5	195	166	184	153	491

Library distribution pre- and post- selection

The abundance of the library members pre- and post- selection were plotted using the ‘frequency distribution’ analysis in GraphPad 10.2.0. The gaussian distribution was also plotted and the amplitude, mean and sigma displayed on the graph.

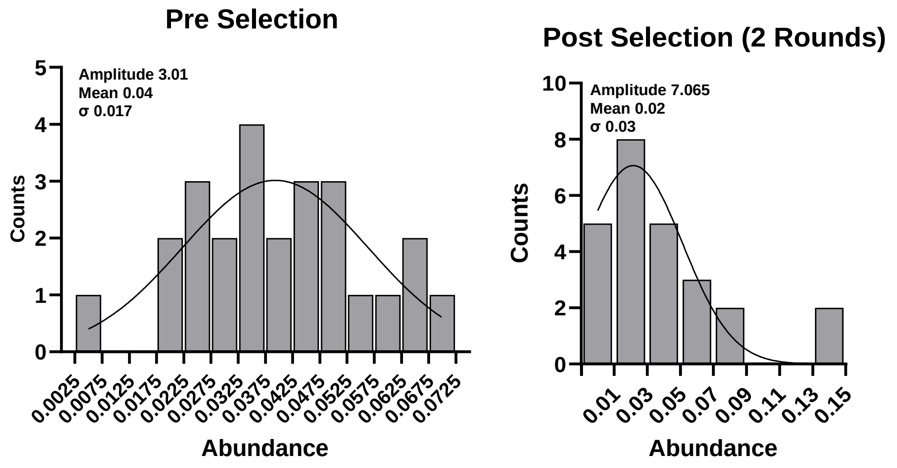

Supplementary Figure 3. Distribution of library pre- (left) and post-selection (right). The distribution is showed by counts versus abundance as a bar chart.

5. Surface Plasmon Resonance Results

All SPR curves are displayed with coloured lines representing different concentrations and the black dotted lines representing the fittings from which the kinetic values are calculated. The compounds

shown in Figure 6 are tested at 250 nM (highest concentration) with a 2-fold dilution series until 3.91 nM (lowest concentration).

SPR Curves of compounds 4.1 and 4.4

4.1: Concentrations tested: 125 nM to 3.91 nM (2-fold dilutions) – K_D: ND

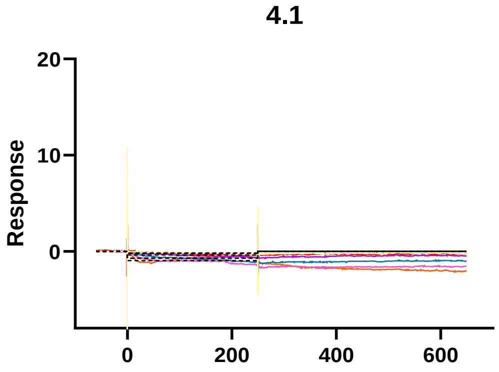

*Supplementary Figure 4. SPR curve of compound **4.1** against MDM2*

4.1: Concentrations tested: 250 nM to 3.91 nM (2-fold dilutions) – K_D: ND

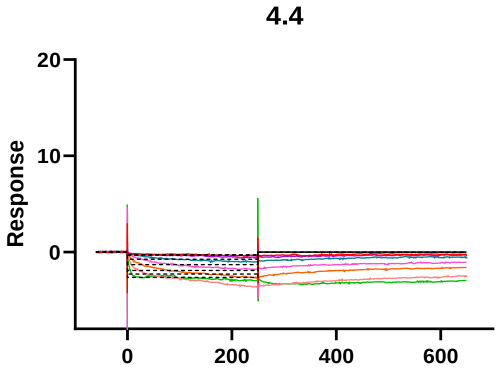

*Supplementary Figure 5. SPR curve of compound **4.4** against MDM2*

6. DNA Sequences

All DNA sequences were purchased from Eurogentec. For the 5x5 library, 25 individual non-hairpin DNA strands were purchased (GTCTATTGCAGGCAAGCTC XXX XXXX XXX YYY YYYY YYY CGAGTCATAGCGCATCGTT) and subjected to individual PCR reactions to incorporate biotin/phosphate on the 5' ends. The individual DNA strands were quantified by nanodrop, combined and subjected to double strand to single strand conversion and ligation prior to use (see section 2).

Supplementary Table 11. DNA Sequences - Primers

**Primers (5' to 3')**		
**Name**	**Sequence**	**Modifications**
Primer1	GT CTA TTG CAG GCA AGC TC	5': Phosphate

(Regeneration FP)		
Primer2 (Regeneration RP)	A ACG ATG CGC TAT GAC TCG	5’: Biotin
Primer3 (Microarray FP)	GTC TAT TGC AGG CAA GCT CCG T	5’: Biotin
Primer 4 (Microarray RP)	A ACG ATG CGC TAT GAC TCG	5’: Cy3

Supplementary Table 12. DNA Sequences

**Sequences (5’ to 3’) – PNA coding region underlined**		
**Name**	**Sequence**	**Modifications**
DNA1	AGCGCAGGTTCCCCTGCGCT GGCTGTCGT GCTAGTGCG	5': NH_2 (C_6 linker)
DNA2	AGCGCAGGTTCCCCTGCGCT GGCTGTCGT GAGCAGTCG GCTAGTGCG	5': NH_2 (C_6 linker)
DNA3	AGC GCA GGT TCC CCT GCG CT GGCTGTCGT GAGCAGTCG CTCGTGCTC GCTAGTGCG	5': NH_2 (C_6 linker)
DNA4	AGC GCA GGT TCC CCT GCG CT GAGCAGTCG CTCGTGCTC TAT CAA AGG CAA	5’: NH_2 (C_6 linker)
DNA1.1 (commercial)	NH2-GAGCTTGCCTGCAATAGACGAATTCGAAAGGAATTC GT CTA TTG CAG GCA AGC TC CGT CGGC ACC CAC GCTT GTC CGA GTC ATA GCG CAT CGT T	5’: NH_2 (C_6 linker)
DNA Hairpin	GAGCTTGCCTGCAATAGACGAATTCGAAAGGAATTC	5’: NH_2 (C_6 linker)

Supplementary Table 13. DNA Sequences - Library

**Library DNA Sequences (5’ to 3’)**		
**NH2-GAGCTTGCCTGCAATAGACGAATTCGAAAGGAATTCGTCTATTGCAGGCAAGCTC XXX XXXX XXX YYY YYYY YYY CGAGTCATAGCGCATCGTT**		
**Name**	**LHS Codon (X region)**	**RHS Codon (Y region)**
1.1	CGT CGGC ACC	CAC GCTT GTC
1.2	CGT CGGC ACC	CGT CTCT GTC
1.3	CGT CGGC ACC	TGC GTCC GTC
1.4	CGT CGGC ACC	GCT CTGC GTC
1.5	CGT CGGC ACC	TCG TCCG GTC
2.1	CGT CCTT GGC	CAC GCTT GTC
2.2	CGT CCTT GGC	CGT CTCT GTC
2.3	CGT CCTT GGC	TGC GTCC GTC
2.4	CGT CCTT GGC	GCT CTGC GTC
2.5	CGT CCTT GGC	TCG TCCG GTC
3.1	CGT GCCG CGT	CAC GCTT GTC
3.2	CGT GCCG CGT	CGT CTCT GTC
3.3	CGT GCCG CGT	TGC GTCC GTC
3.4	CGT GCCG CGT	GCT CTGC GTC
3.5	CGT GCCG CGT	TCG TCCG GTC

4.1	CGT TTCC GCT	CAC GCTT GTC
4.2	CGT TTCC GCT	CGT CTCT GTC
4.3	CGT TTCC GCT	TGC GTCC GTC
4.4	CGT TTCC GCT	GCT CTGC GTC
4.5	CGT TTCC GCT	TCG TCCG GTC
5.1	CGT CTTG CCG	CAC GCTT GTC
5.2	CGT CTTG CCG	CGT CTCT GTC
5.3	CGT CTTG CCG	TGC GTCC GTC
5.4	CGT CTTG CCG	GCT CTGC GTC
5.5	CGT CTTG CCG	TCG TCCG GTC

7. MDM2 Crystal Structure Analysis

Crystal Structure of stapled MDM2 peptide (pdb 5AFG)[1].
Sequence: TFAEYWAQLAS. Measured distance is between T (amine) and S (carbonyl): 16.6 Å

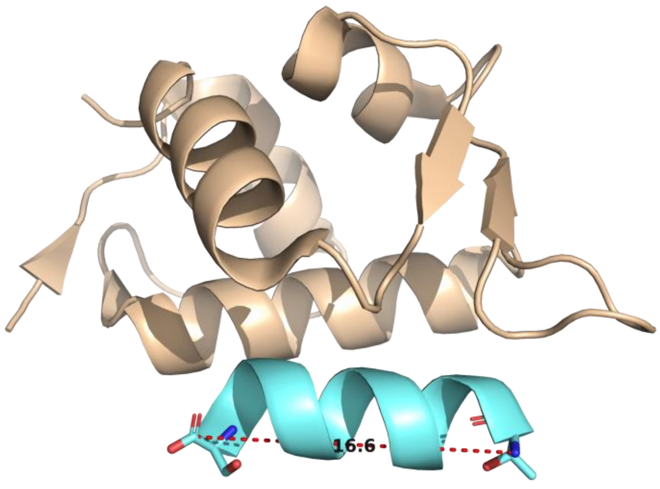

Supplementary Figure 6. X-ray crystal structure of MDM2 inhibitor with MDM2 (pdb: 5AFG).

8. Synthesis of Purification Tag

2-chloroethyl 4-methylbenzenesulfonate

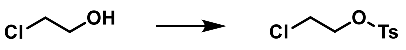

Chemical Formula: $C_9H_{11}ClO_3S$, **Exact Mass**: 234.01, **Molecular Weight**: 234.69.

To a stirred solution of 2-chloroethan-1-ol (1.0 g, 12.5 mmol) and triethylamine (1.5 eq., 18.6 mmol, 2.6 mL) in DCM (50 mL, 0.25 M), TsCl (1.5 eq., 18.6 mmol, 3.55 g) was added. The reaction was stirred for 24 hours. Water (50 mL) was added and the product was extracted with DCM (2x), the organic layers were washed with Brine (1x), dried over Na_2SO_4, and concentrated. The crude material was purified by flash chromatography (10% EtOAc in pentane) to yield the product (1.92g, 8.18 mmol, 65%) as a colourless oil.

NMR; ^{1}H NMR (400 MHz, $CDCl_3$) δ 7.86 – 7.78 (m, 2H), 7.41 – 7.33 (m, 2H), 4.16 (t, 2H), 3.49 (t, *J* = 5.1 Hz, 2H), 2.46 (s, 3H).

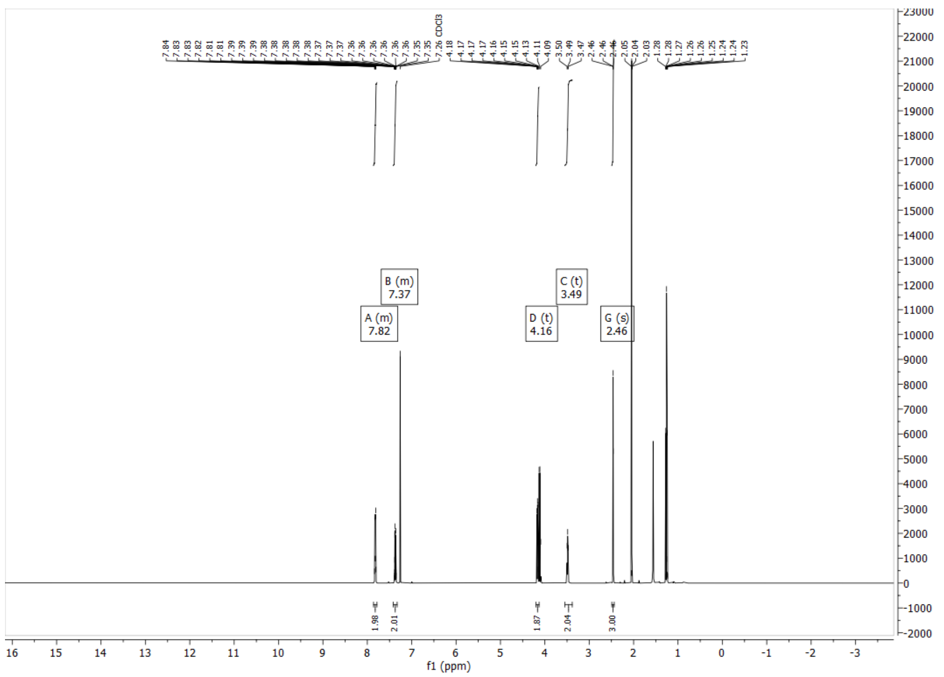

1-(4-(2-chloroethoxy)-3-methoxyphenyl)ethan-1-one

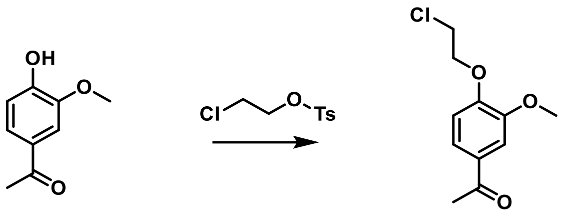

Chemical Formula: $C_{11}H_{13}ClO_3$, **Exact Mass**: 228.055, **Molecular Weight**: 228.672

2-chloroethyl 4-methylbenzenesulfonate (1.1 eq., 1.82 g, 7.78 mmol) was added to a mixture of acetonvanillone (1.16 g, 6.98) and potassium carbonate (1.5 eq, 1.44 g, 10.47 mmol) in dry DMF (21.7 mL, 0.3 M). The reaction was heated to 90 °C overnight. The reaction was cooled to room temperature, concentrated and azeotroped with toluene. The crude material was purified by flash chromatography (30% EtOAc in pentane) to yield the product (1.58 g, 6.90 mmol, Quant.).

NMR; ^{1}H NMR (400 MHz, $CDCl_3$) δ 7.57 – 7.54 (m, 2H), 6.93 – 6.87 (m, 1H), 4.24 (t, 2H), 3.92 (s, 3H), 3.69 (t, *J* = 5.1 Hz, 2H), 2.57 (s, 3H).

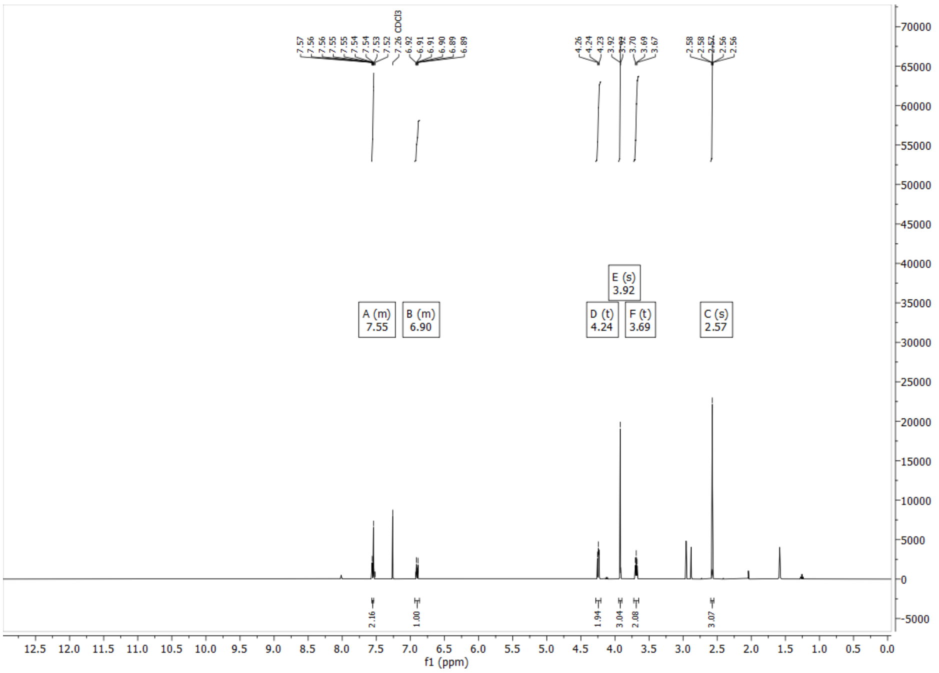

1-(4-(2-chloroethoxy)-5-methoxy-2-nitrophenyl)ethan-1-one

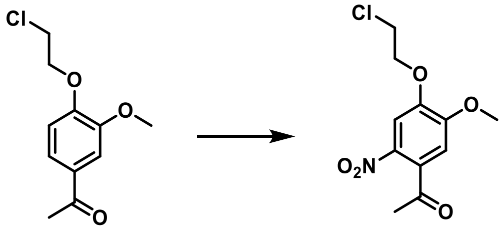

Chemical Formula: $C_{11}H_{12}ClNO_5$, **Exact Mass**: 273.04, **Molecular Weight**: 273.67.

Fuming nitric acid (4.05 mL) was added to 1-(4-(2-chloroethoxy)-3-methoxyphenyl)ethan-1-one (1.6 g, 7.02 mmol) in DCM (20.5 mL, 0.3 M) at 0 °C. The reaction was warmed to room temperature. After 20 minutes, water (40 mL) was added and the product was extracted with DCM (3x), the organic layers were washed with brine (1x), dried over Na_2SO_4, and concentrated. The crude material was purified by flash chromatography (30% EtOAc in pentane) to yield the product (0.77 g, 2.82 mmol, 40%) as a colourless oil.

NMR; ^{1}H NMR (400 MHz, $CDCl_3$) δ 7.63 (s, 1H), 6.77 (s, 1H), 4.92 – 4.85 (m, 2H), 4.42 – 4.33 (m, 2H), 3.97 (s, 3H), 2.50 (s, 3H).

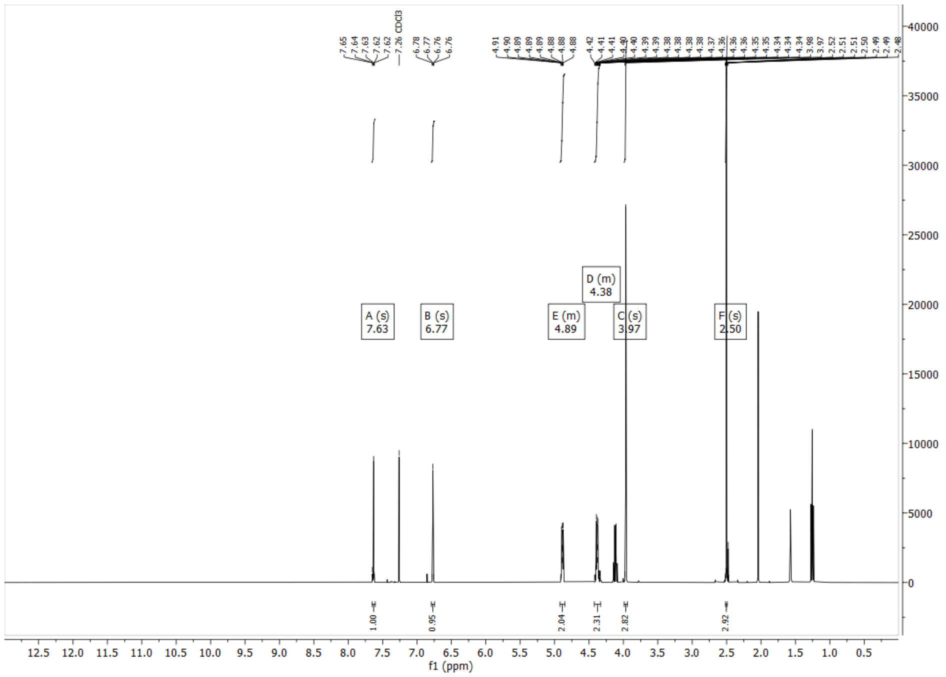

1-(4-(2-azidoethoxy)-5-methoxy-2-nitrophenyl)ethan-1-one

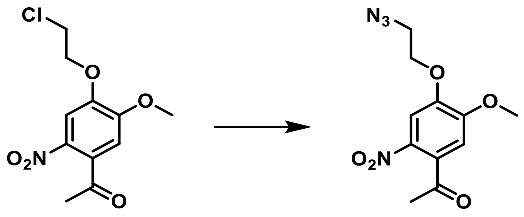

Chemical Formula: $C_{11}H_{12}N_4O_5$, **Exact Mass**: 280.08, **Molecular Weight**: 280.24.

Sodium Azide (4 eq., 11.13 mmol, 0.74 g) was added to 1-(4-(2-chloroethoxy)-5-methoxy-2-nitrophenyl)ethan-1-one (0.772g, 2.82 mmol) in DMF (25 mL, 0.1 M) and heated to 60 °C overnight. Water (50 mL) was added and the product was extracted with ether (3x), the organic layers were washed with brine (1x), dried over Na_2SO_4, and concentrated to yield a yellow solid (0.56 g, 2.0 mmol, 71%). The product was used without further purification.

NMR; ^{1}H NMR (400 MHz, $CDCl_3$) δ 7.63 (s, 1H), 6.77 (s, 1H), 4.30 – 4.23 (m, 2H), 3.97 (s, 3H), 3.70 (dd, *J* = 5.4, 4.5 Hz, 2H), 2.50 (s, 3H).

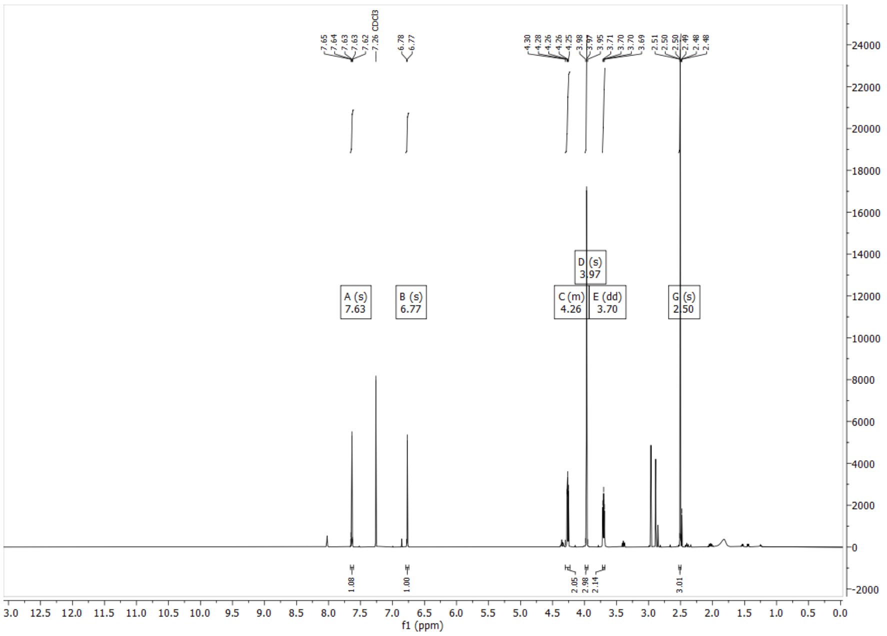

1-(4-(2-azidoethoxy)-5-methoxy-2-nitrophenyl)ethan-1-ol

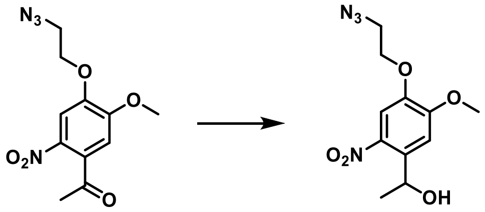

Chemical Formula: $C_{11}H_{14}N_4O_5$, **Exact Mass**: 282.10, **Molecular Weight**: 282.26.

Sodium borohydride (1.5 eq., 2.7 mmol, 102.6 mg) was added to 1-(4-(2-azidoethoxy)-5-methoxy-2-nitrophenyl)ethan-1-one (0.5 g, 1.8 mmol) in methanol (4.5 mL, 0.4 M) at 0 °C. The reaction was warmed to room temperature and stirred for 30 minutes. Saturated NH_4Cl (10 mL) was added and the product was extracted with DCM (3x), the organic layers were washed with brine (3x), dried over Na_2SO_4, and concentrated. The crude material was purified by flash chromatography (30% EtOAc in pentane) to yield the product (0.350 g, 1.24 mmol, 69%).

NMR; ^{1}H NMR (400 MHz, $CDCl_3$) δ 7.60 (s, 1H), 7.33 (s, 1H), 5.58 (q, *J* = 6.3 Hz, 1H), 4.27 – 4.19 (m, 2H), 3.99 (s, 3H), 3.72 – 3.64 (m, 2H), 1.56 (d, *J* = 6.3 Hz, 3H).

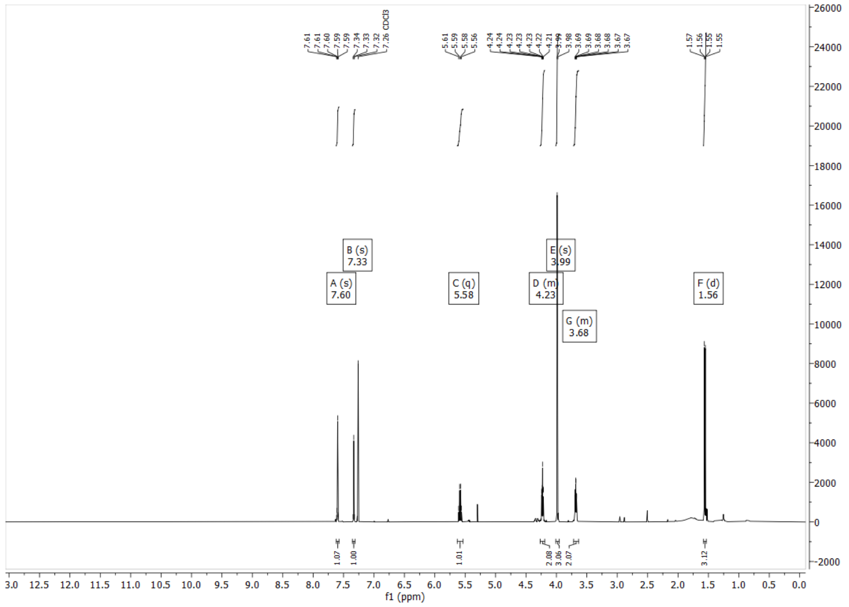

1-(4-(2-azidoethoxy)-5-methoxy-2-nitrophenyl)ethyl (4-nitrophenyl) carbonate

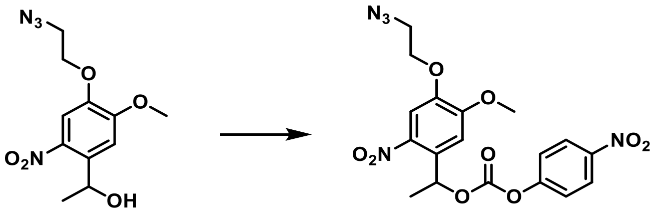

Chemical Formula: $C_{18}H_{17}N_5O_9$, **Exact Mass**: 447.10, **Molecular Weight**: 447.36

Triethylamine (2.3 eq. 13.2 mmol, 1.44 mL) was added to a solution of 1-(4-(2-azidoethoxy)-5-methoxy-2-nitrophenyl)ethan-1-ol (1.86 g, 6.60 mmol), 4-nitrophenol chloroformate (1.65 eq., 10.9 mmol, 2.2 g), 4-Dimethylaminopyridine (0.5 eq., 3.30 mmol, 0.402 g) in dry DCM (33 mL, 0.2 M). The reaction was stirred overnight under nitrogen at room temperature. Water (50 mL) was added and the product was extracted with DCM (3x), the organic layers were washed with brine (3x), dried over Na_2SO_4, and concentrated. The crude material was purified by flash chromatography (20% EtOAc in pentane) to yield the product (2.3g) as an impure mixture with 4-nitrophenol which co-eluted (1:0.43 product:nitrophenol). The mixture was used in the next step without further purification.

NMR; ^{1}H NMR (400 MHz, $CDCl_3$) δ 8.19 – 8.10 (m, 2H), 7.53 (s, 1H), 7.29 – 7.20 (m, 2H), 7.05 (s, 1H), 6.45 (q, *J* = 6.4 Hz, 1H), 4.15 (t, *J* = 5.4, 4.5 Hz, 2H), 3.91 (s, 3H), 3.59 (t, *J* = 5.4, 4.5 Hz, 2H), 1.68 (d, *J* = 6.4 Hz, 3H).

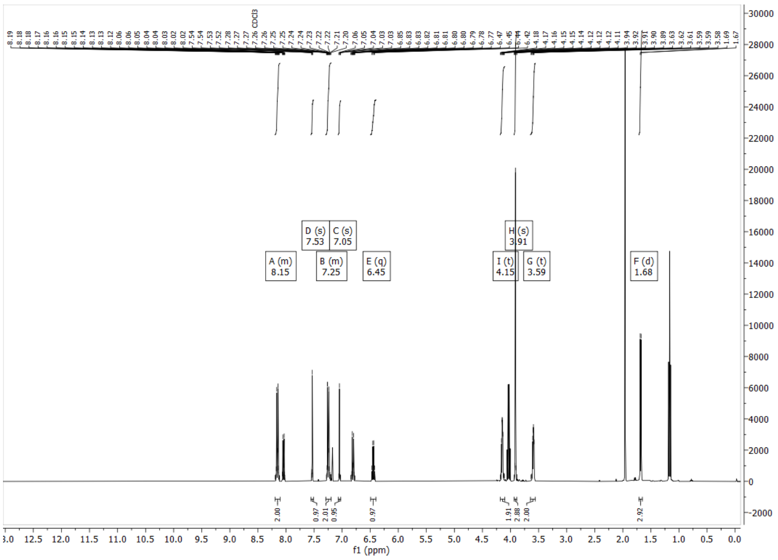

N^{2}-(((9H-fluoren-9-yl)methoxy)carbonyl)-N^{6}-((1-(4-(2-azidoethoxy)-5-methoxy-2-nitrophenyl)ethoxy)carbonyl)-L-lysine (Fmoc-Lys(PL-N$_3$)-OH)

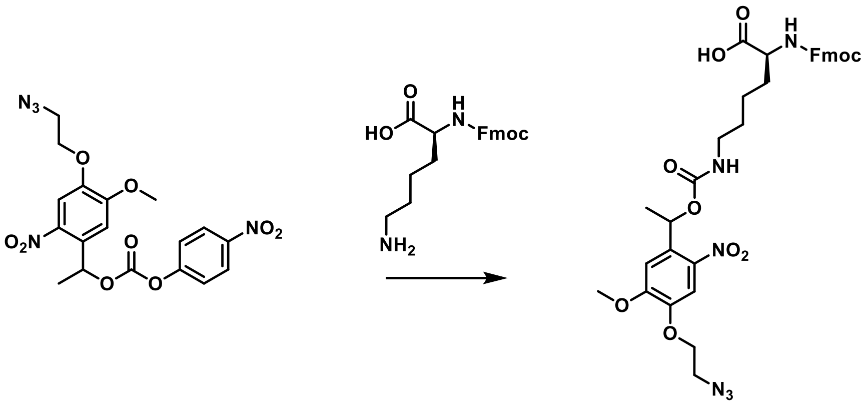

Chemical Formula: $C_{33}H_{36}N_6O_{10}$, **Exact Mass**: 676.25, **Molecular Weight**: 676.68

DIPEA (15.45 mmol, 2.7 mL) was added to a mixture of 1-(4-(2-azidoethoxy)-5-methoxy-2-nitrophenyl)ethyl (4-nitrophenyl) carbonate (2.3 g crude), Fmoc-Lysine-OH (2.27 g, 6.18 mmol) in THF (26 mL) and H_2O (26 mL) and the reaction was stirred for 2 hours. The THF was removed *under vacuo*. The remaining aqueous solution was acidified and extracted with DCM (2x). The organic layer was concentrated, redissolved in DMF and purified reverse phase chromatography (Biotage, 60g C18 column, 30-100% ACN in H_2O) to yield the product (1.64 g, 2.42 mmol, 37 % over two steps).

LCMS (ESI); RT= 3.12, $[M+1H]^{1+}$: 676.50. Main Adduct is -NO_2, $[M\text{-}NO_2+1H]^{1+}$: 633.00. $[M+Na]^{1+}$: 699.08 is also observed.

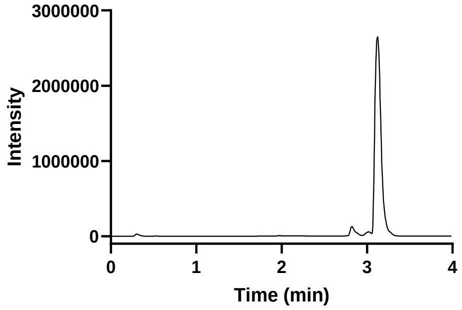

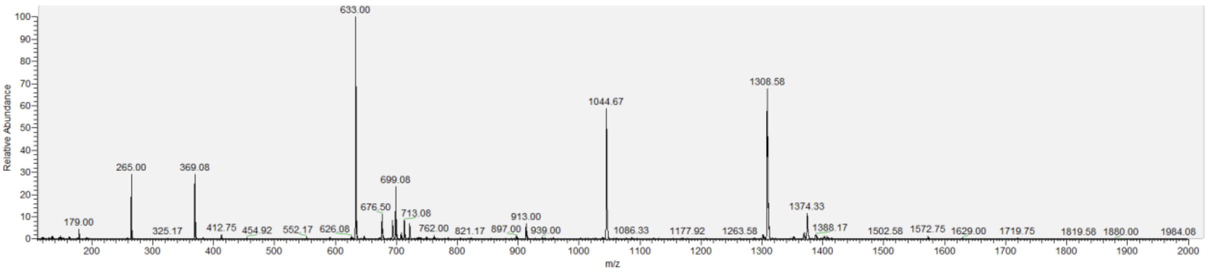

NMR; ^{1}H NMR (400 MHz, DMSO) δ 7.90 – 7.83 (m, 2H), 7.73 – 7.66 (m, 2H), 7.61 (s, 1H), 7.59 – 7.54 (m, 1H), 7.43 – 7.36 (m, 3H), 7.30 (td, *J* = 7.4, 1.3 Hz, 2H), 7.11 (s, 1H), 6.14 – 6.04 (m, 1H), 4.28 – 4.15 (m, 5H), 3.91 – 3.81 (m, 4H), 3.65 (t, *J* = 4.8 Hz, 2H), 2.94 – 2.85 (m, 2H), 1.71 – 1.52 (m, 2H), 1.50 (d, *J* = 6.4 Hz, 3H), 1.38 – 1.21 (m, 4H).

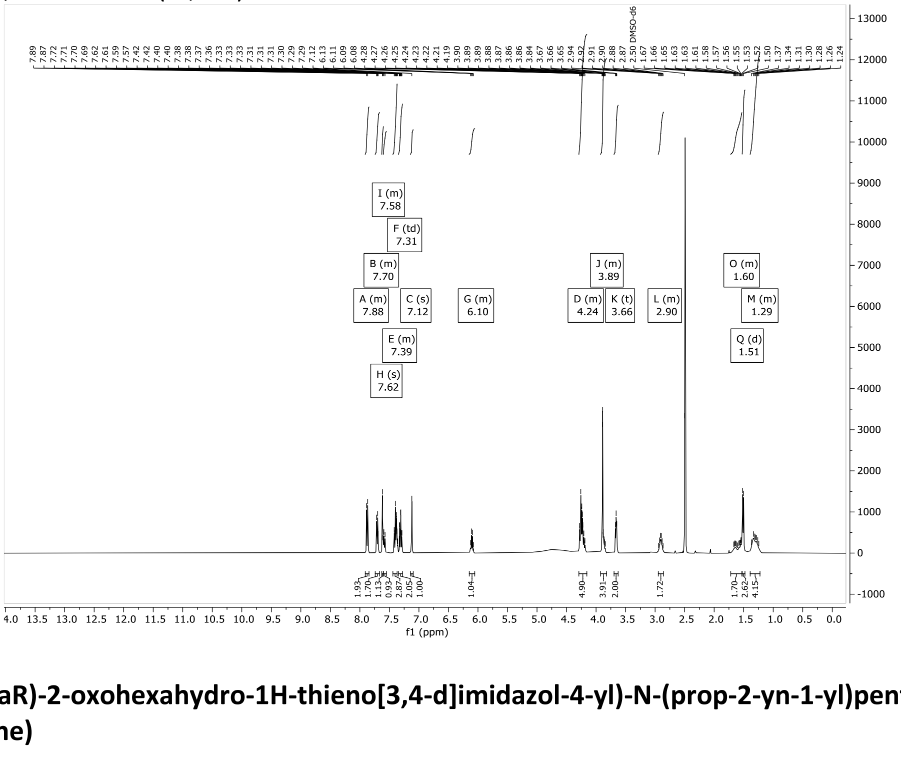

5-((3aS,4S,6aR)-2-oxohexahydro-1H-thieno[3,4-d]imidazol-4-yl)-N-(prop-2-yn-1-yl)pentanamide (Biotin-alkyne)

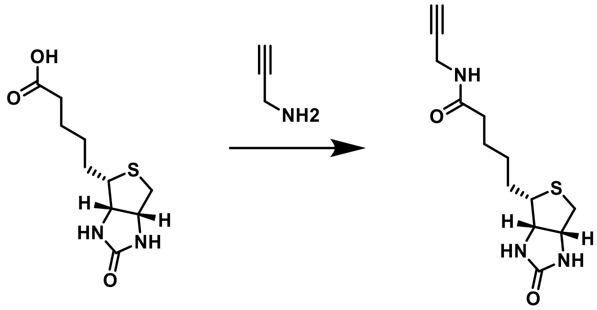

Chemical Formula: $C_{13}H_{19}N_3O_2S$, **Exact Mass**: 281.12, **Molecular Weight**: 281.37

EDC (1.6 eq, 0.64 mmol, 123 mg) was added to a mixture of biotin (100 mg, 0.41 mmol), propargylamine (1.1 eq., 0.44 mmol, 24 mg) and HOBT (1.5 eq., 0.62 mmol, 84 mg) in water (9 mL) and acetonitrile (9 mL) at 0 °C. The reaction was stirred at room temperature overnight. The mixture was concentrated, redissolved in DMSO (2 mL) and purified reverse phase chromatography (Biotage, 48g C18 column, 5-50% ACN in H_2O) to yield the product (97.8 mg, 0.35 mmol, 85%).

LCMS (ESI); RT= 1.29, $[M+1H]^{1+}$: 282.09.

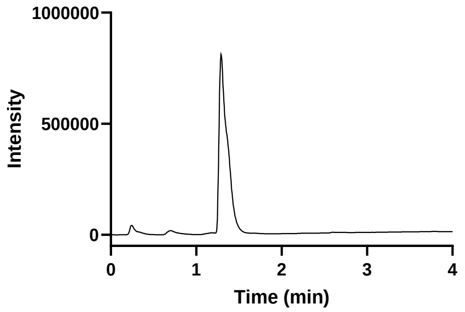

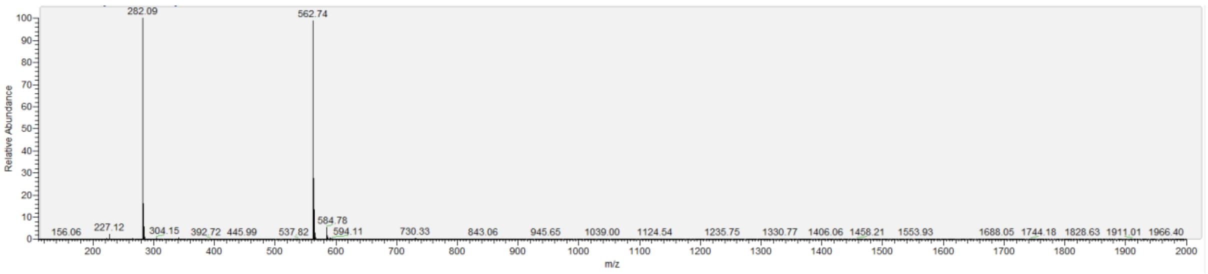

NMR; ^{1}H NMR (400 MHz, DMSO) δ 8.20 (t, *J* = 5.5 Hz, 1H), 6.36 (s, 2H), 4.32 – 4.24 (m, 1H), 4.17 – 4.07 (m, 1H), 3.84 – 3.78 (m, 2H), 3.12 – 3.02 (m, 2H), 2.84 – 2.75 (m, 1H), 2.59 – 2.51 (m, 1H), 2.06 (t, *J* = 7.4 Hz, 2H), 1.65 – 1.37 (m, 4H), 1.28 (pq, *J* = 11.3, 5.5, 4.7 Hz, 2H).

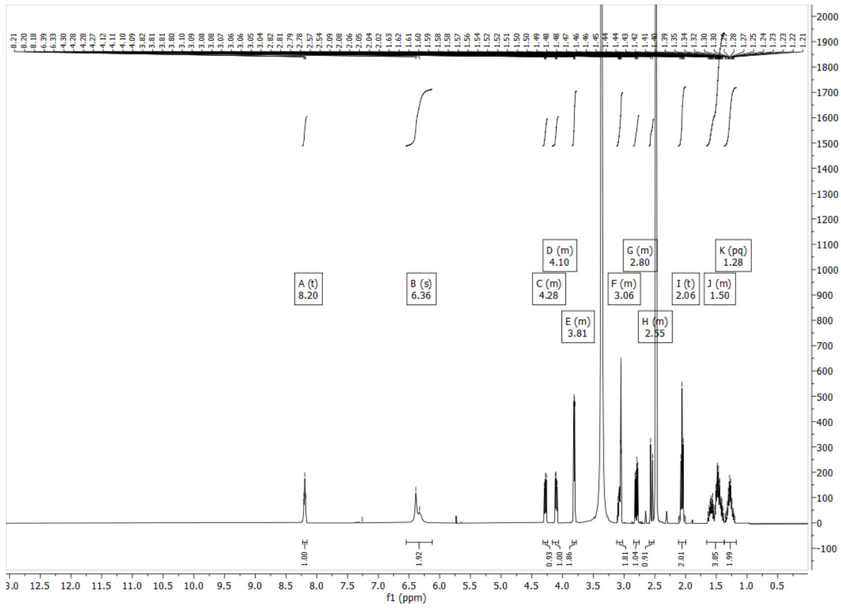

N₂-(((9H-fluoren-9-yl)methoxy)carbonyl)-N₆-((1-(5-methoxy-2-nitro-4-(2-(4-((5-((3aS,4S,6aR)-2-oxohexahydro-1H-thieno[3,4-d]imidazol-4-yl)pentanamido)methyl)-1H-1,2,3-triazol-1-yl)ethoxy)phenyl)ethoxy)carbonyl)-L-lysine (Fmoc-Lys(PL-Biotin)-OH)

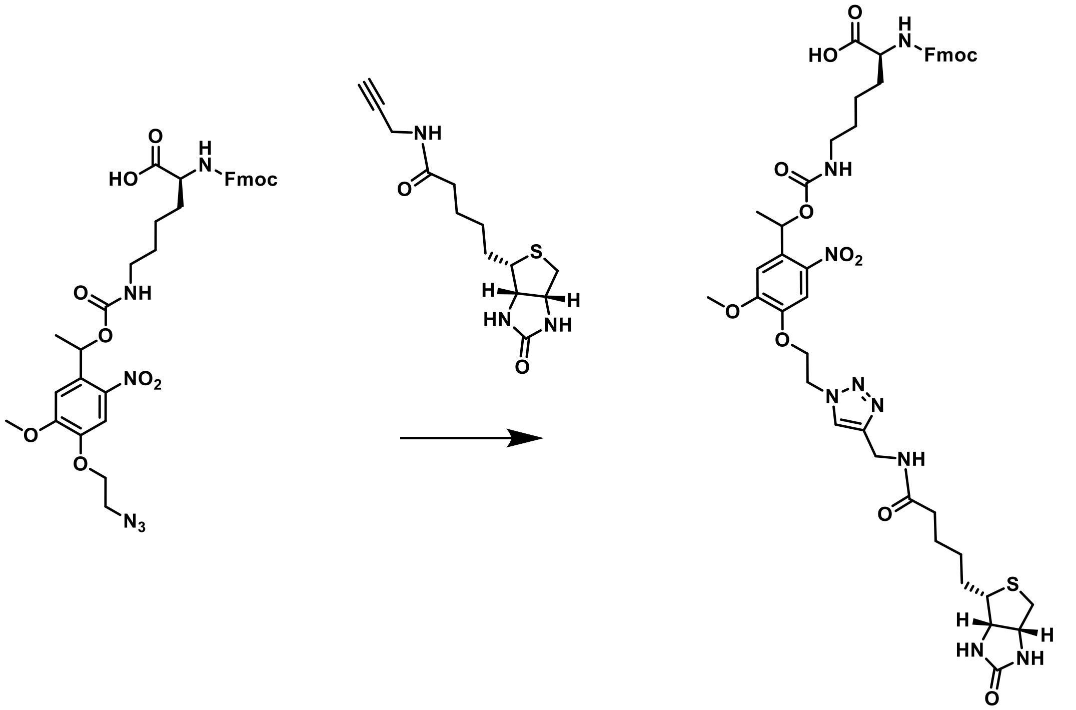

Chemical Formula: $C_{46}H_{55}N_9O_{12}S$, **Exact Mass**: 957.37, **Molecular Weight**: 958.06.

(Fmoc-Lys(PL-N_3)-OH (400 mg, 0.6 mmol) and biotin alkyne (4.5 eq., 2.7 mmol, 750 mg) were dissolved in 9:1 DMF:H_2O (3 mL). Cu(I) (0.8 eq., 0.48 mmol, 92 mg), DIPEA (0.8 eq., 0.48 mmol, 84 µL) and NaASC (1.6 eq., 0.96 mmol, 190 mg) in 3 mL DMSO were added. The reaction was heated to 60 °C for two hours. The reaction was filtered and purified by purified reverse phase chromatography (2x3mL injections, Biotage, 60g C18 column, 10-100% ACN in H_2O) to yield the product (469 mg, 0.49 mmol, 82%).

LCMS (ESI); RT= 2.53, $[M+1H]^{1+}$: 958.22.

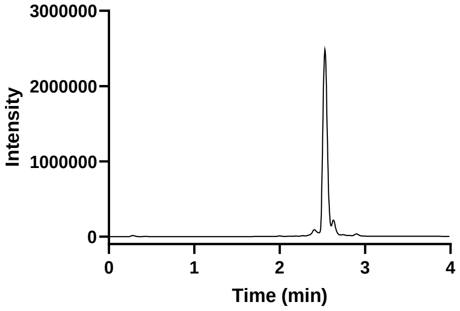

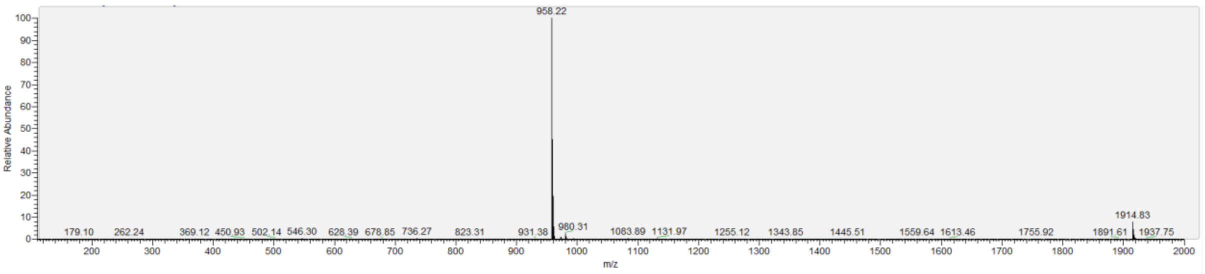

NMR; ^{1}H NMR (400 MHz, DMSO) δ 8.33 – 8.17 (m, 1H), 7.94 (s, 1H), 7.87 (d, *J* = 7.5 Hz, 2H), 7.72 – 7.66 (m, 2H), 7.62 – 7.53 (m, 2H), 7.45 – 7.26 (m, 5H), 7.09 (s, 1H), 6.38 (s, 1H), 6.08 (q, 1H), 4.72 (t, *J* = 5.1 Hz, 5H), 4.47 (t, *J* = 5.1 Hz, 3H), 4.32 – 4.15 (m, 6H), 4.12 – 4.01 (m, 1H), 3.86 (s, 4H), 3.10 – 2.98 (m, 1H), 2.96 – 2.84 (m, 2H), 2.82 – 2.74 (m, 1H), 2.10 – 2.00 (m, 2H), 1.59 – 1.44 (m, 7H), 1.38 – 1.21 (m, 6H).

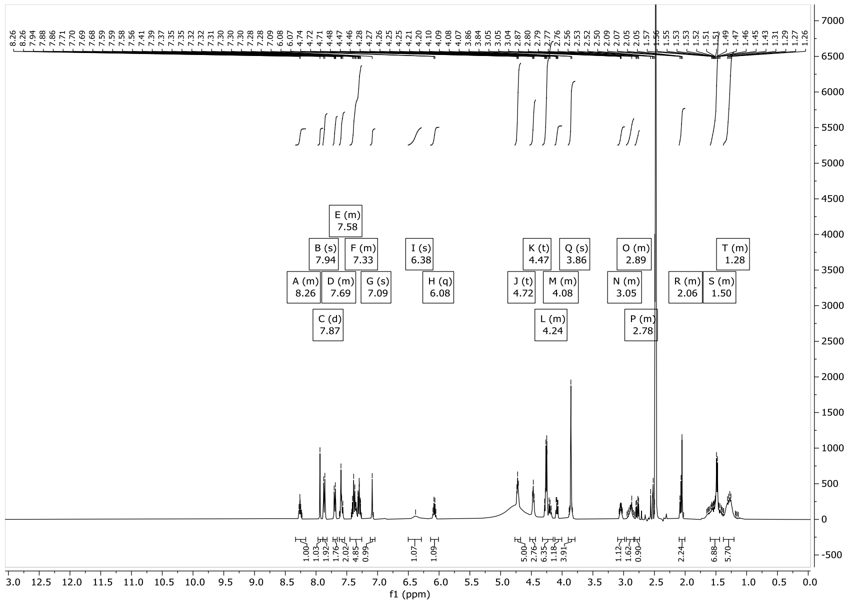

9. Characterisation of PNA, PNA-peptide and peptide compounds

i) PNA Strands

PNA 1 (Figure 2)

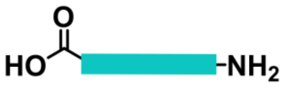

HOOC-Gly-CCGACAGCA-NH_2

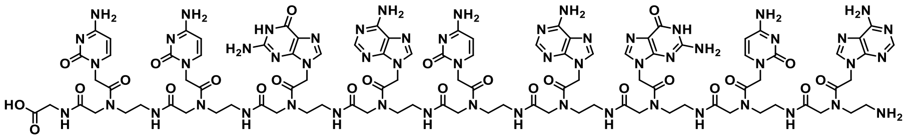

Chemical Formula: $C_{97}H_{122}N_{56}O_{26}$, **Exact Mass**: 2487.00, **Molecular Weight**: 2488.41.

MALDI-TOF; m/z found: 2488.66.

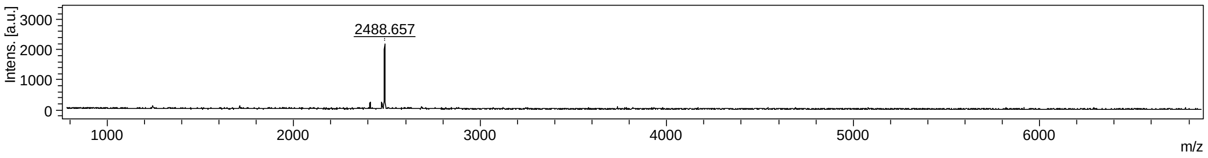

LCMS (ESI); RT= 1.06 min, $[M+2H]^{2+}$: 1245.08, $[M+3H]^{3+}$: 830.50, $[M+4H]^{4+}$: 623.08.

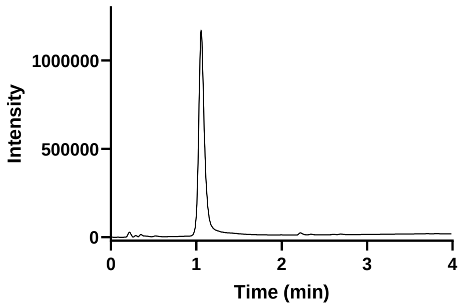

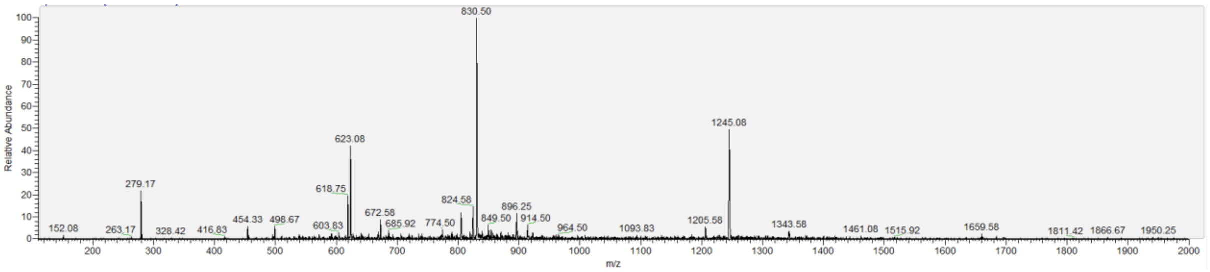

PNA 2 (Figure 2)

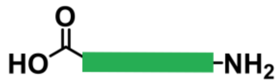

HOOC-Gly-CTCGTCAGC-NH_2

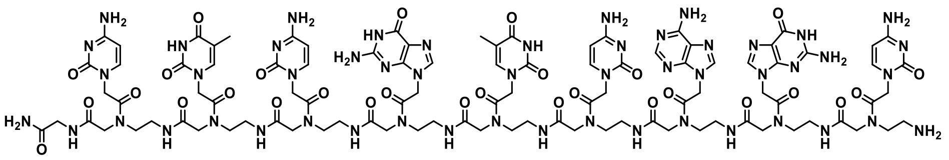

Chemical Formula: $C_{97}H_{125}N_{51}O_{29}$, **Exact Mass**: 2467.99, **Molecular Weight**: 2469.40.

MALDI-TOF; m/z found: 2469.56.

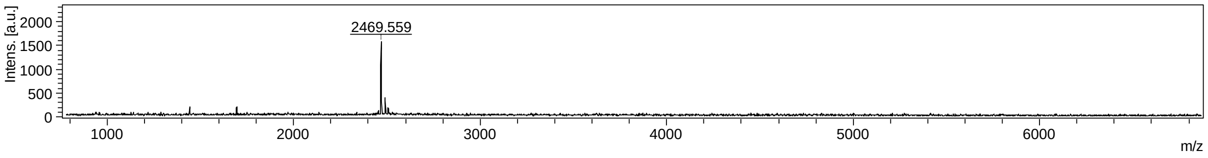

LCMS (ESI); RT= 1.07 min, $[M+2H]^{2+}$: 1236.08, $[M+3H]^{3+}$: 824.50, $[M+4H]^{4+}$: 618.75.

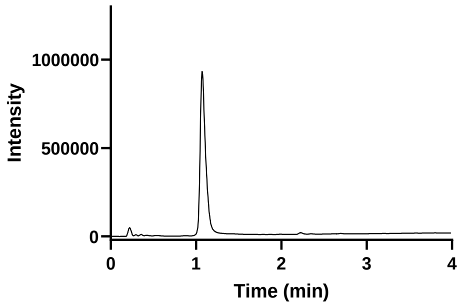

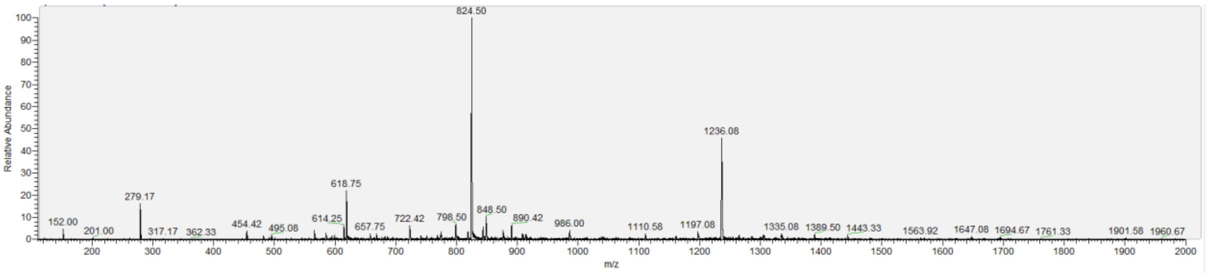

PNA 3 (Figure 2)

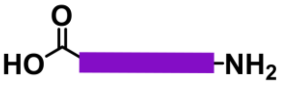

HOOC-Gly-GAGCACGAG-NH_2

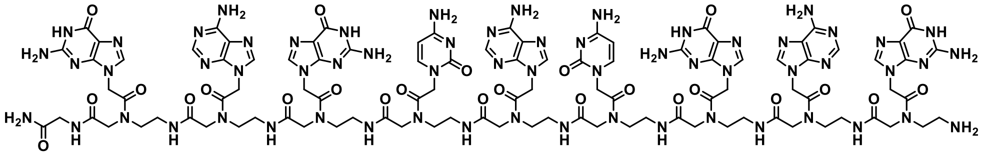

Chemical Formula: $C_{99}H_{123}N_{61}O_{25}$, **Exact Mass**: 2566.02, **Molecular Weight**: 2567.48.

MALDI-TOF; m/z found: 2568.03.

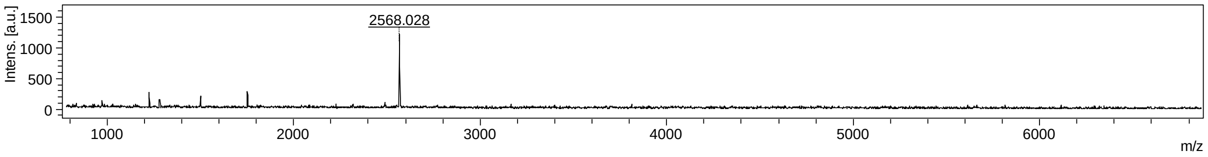

LCMS (ESI); RT= 1.08 min, $[M+2H]^{2+}$: 1285.17, $[M+3H]^{3+}$: 857.25, $[M+4H]^{4+}$: 643.17.

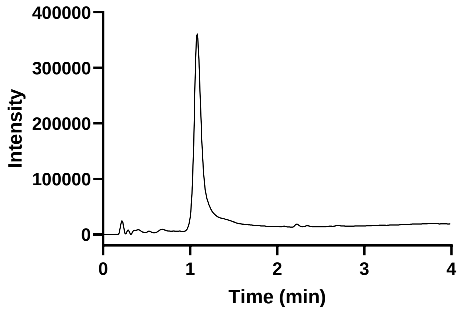

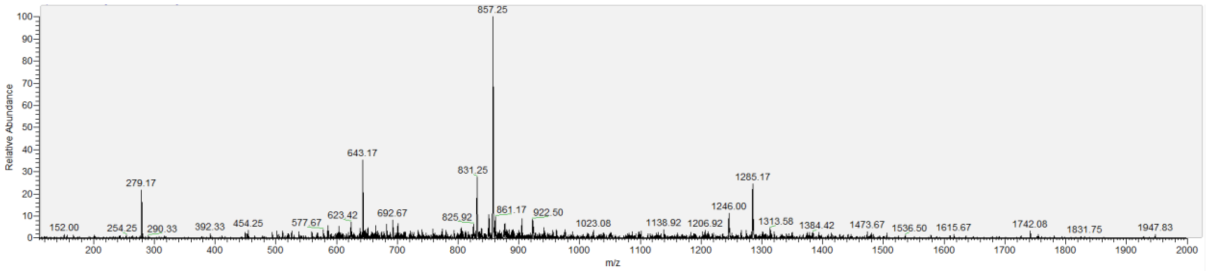

PNA 4 (Figure 2)

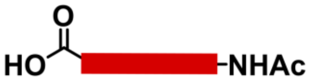

HOOC-Gly-CGATCACGC-NHAc

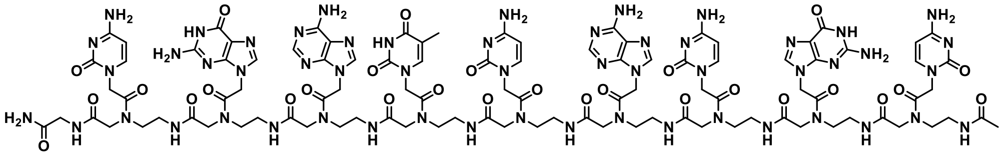

Chemical Formula: $C_{99}H_{126}N_{54}O_{28}$, **Exact Mass**: 2519.01, **Molecular Weight**: 2520.45.

MALDI-TOF; m/z found: 2521.26.

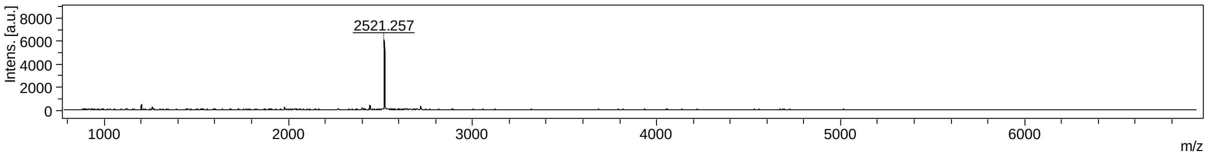

LCMS (ESI); RT= 1.11 min, $[M+2H]^{2+}$: 1285.17, $[M+3H]^{3+}$: 857.25, $[M+4H]^{4+}$: 643.17.

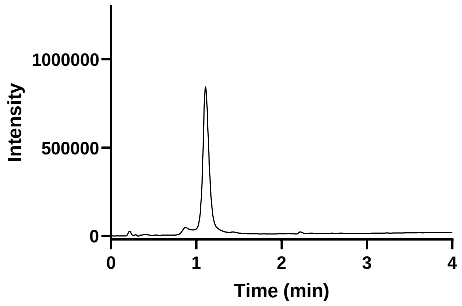

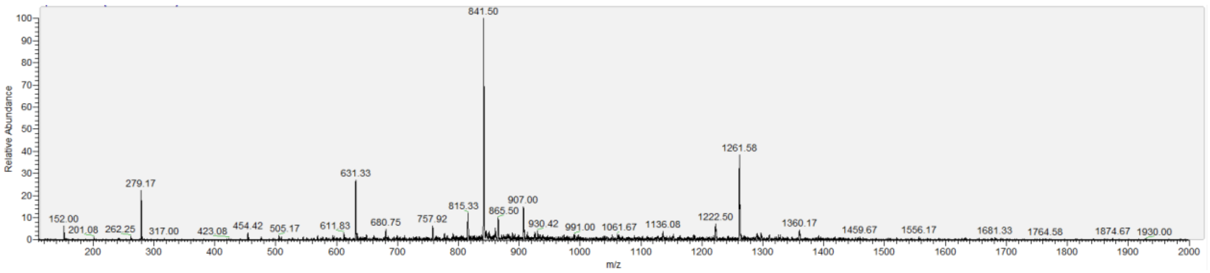

ii) PNA-peptides

PNA-Peptide1 (Figure 3)

HOOC-Gly-CCGACAGCA-Gly-Ser-nLe-Cba-Gln-Ala-NH_2

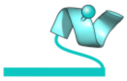

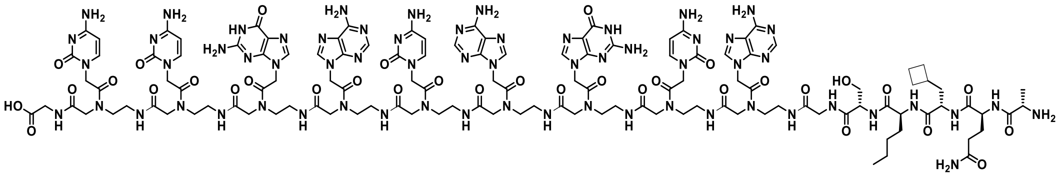

Chemical Formula: $C_{123}H_{165}N_{63}O_{34}$, **Exact Mass**: 3068.31, **Molecular Weight**: 3070.08.

MALDI-TOF; m/z found: 3069.67.

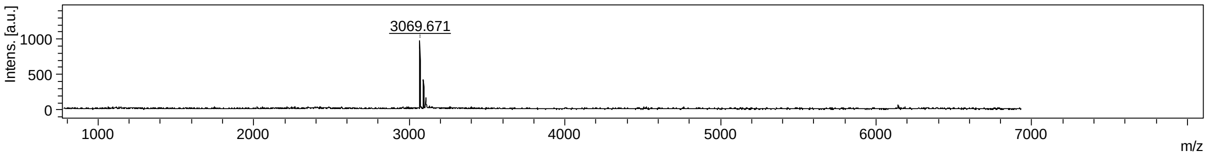

LCMS (ESI); RT= 1.28 min, $[M+2H]^{2+}$: 1535.92, $[M+3H]^{3+}$: 1024.33, $[M+4H]^{4+}$: 768.67, $[M+5H]^{5+}$: 615.00.

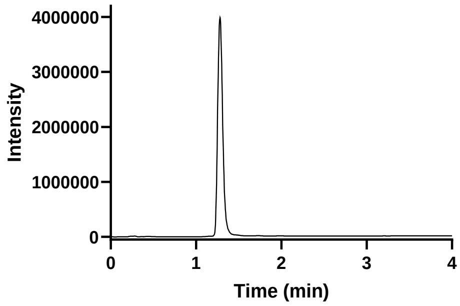

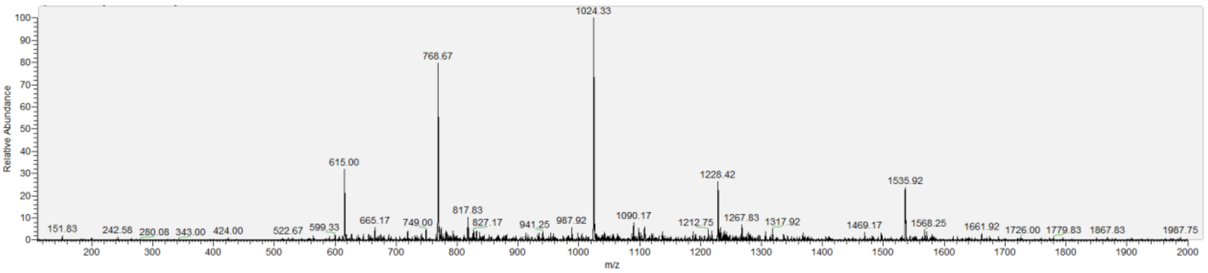

PNA-Peptide2 (Figure 3)

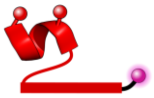

HOOC-Trp-Tyr-Gln-nLe-Phe-Thr-Leu-Gly-CGATCACGC-Cy3

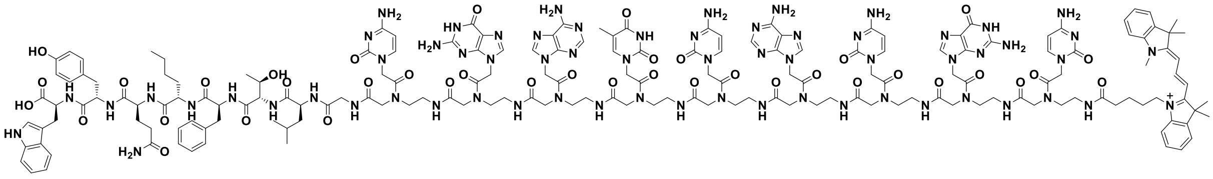

Chemical Formula: $C_{176}H_{221}N_{64}O_{39}^{+}$, **Exact Mass**: 3854.73, **Molecular Weight**: 3857.11.

MALDI-TOF; m/z found: 3856.41.

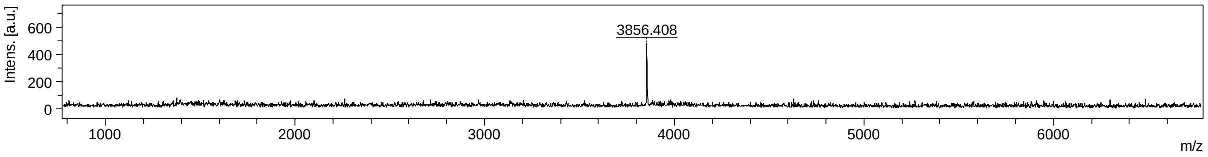

LCMS (ESI); RT= 2.10 min, $[M+3H]^{3+}$: 1286.17, $[M+4H]^{4+}$: 965.08, $[M+5H]^{5+}$: 772.17.

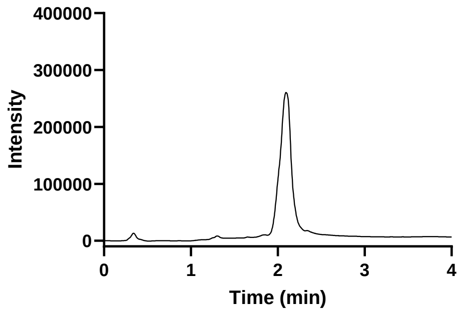

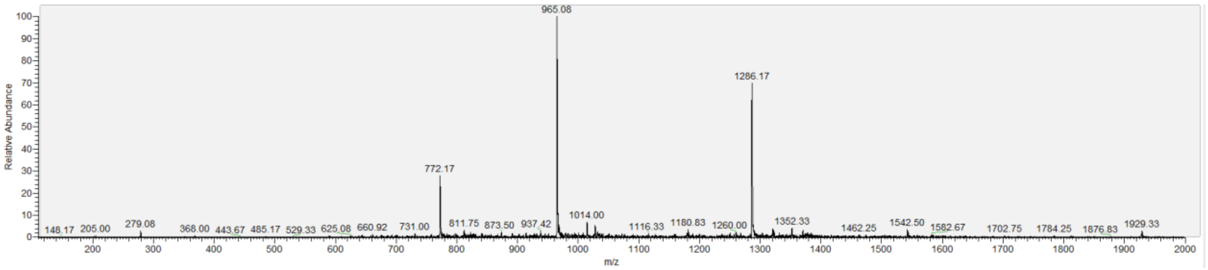

<u>**PNA-Peptide3 (Figure 3)**</u>

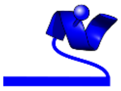

HOOC-Gly-CTCGTCAGC-Gly-Ser-nLe-Ala-Gln-Ala-NH_2

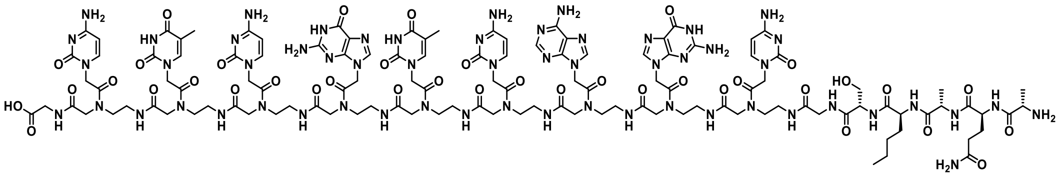

Chemical Formula: $C_{119}H_{161}N_{57}O_{38}$, **Exact Mass**: 2996.24, **Molecular Weight**: 2997.96.

MALDI-TOF; m/z found: 2997.97.

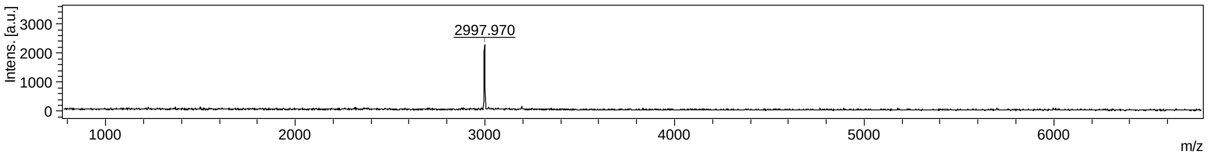

LCMS (ESI); RT= 1.22 min, $[M+2H]^{2+}$: 1499.67, $[M+3H]^{3+}$: 1000.25, $[M+4H]^{4+}$: 750.58, $[M+5H]^{5+}$: 600.67.

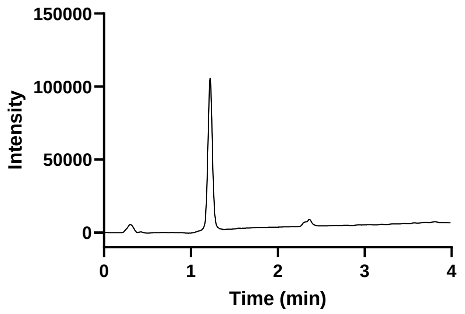

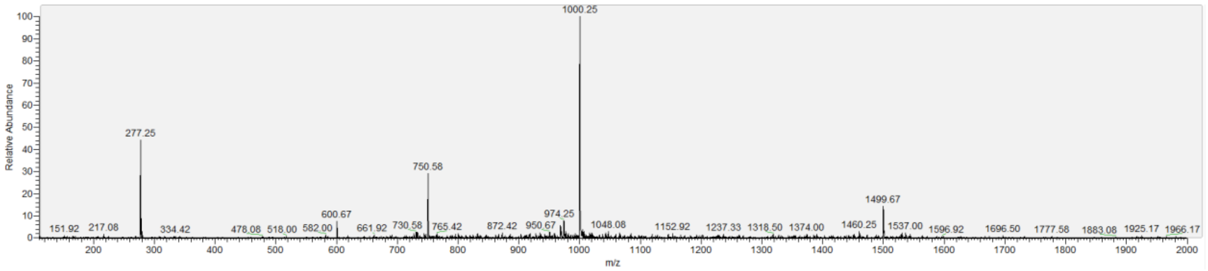

PNA-Peptide4 (Figure 3)

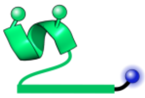

HOOC-Ala-Tyr-Gln-nLe-Ala-Thr-Leu-Gly- GAGCACGAG-Atto647N

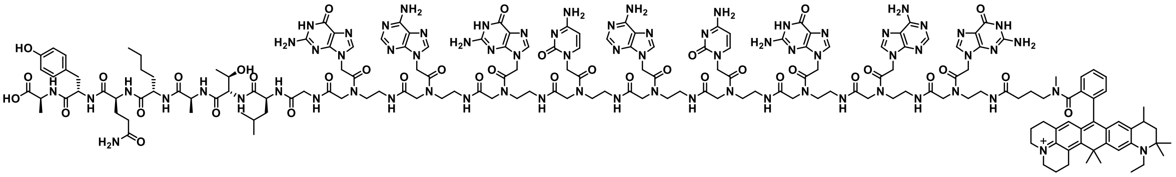

Chemical Formula: $C_{177}H_{228}N_{71}O_{38}^{+}$, **Exact Mass**: 3955.81, **Molecular Weight**: 3958.23.

MALDI-TOF; m/z found: 3958.38.

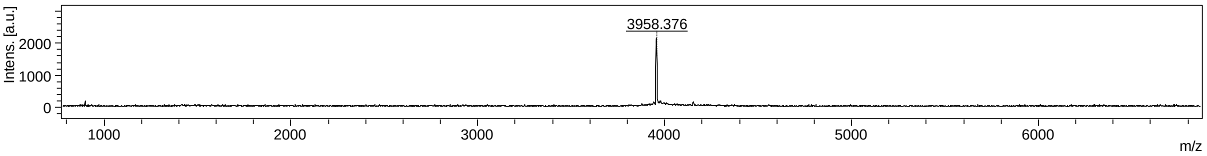

LCMS (ESI); RT= 2.15 min, $[M+3H]^{3+}$: 1320.08, $[M+4H]^{4+}$: 990.25, $[M+5H]^{5+}$: 792.50.

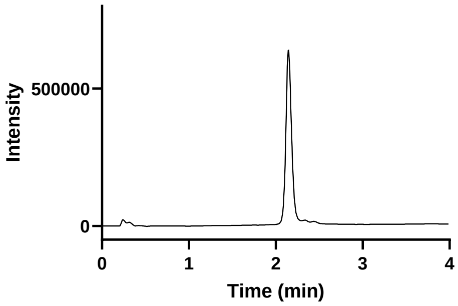

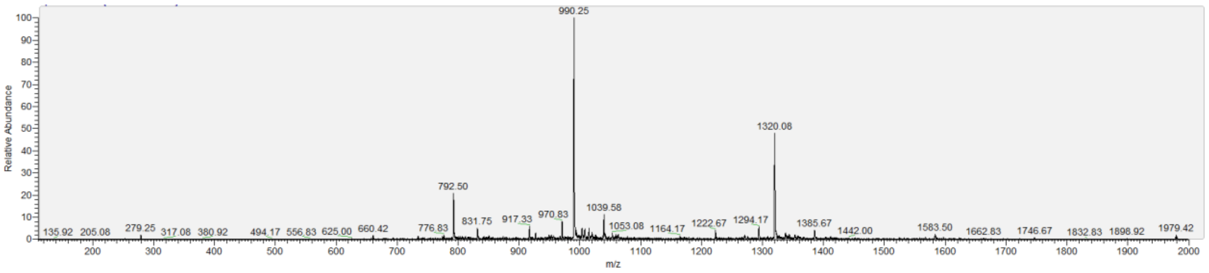

LHS1 (Figure 5)

HOOC-Gly-GCAGCCGTGG-Gly-Ser-Nle-Cba-Gln-Ala-NH_2

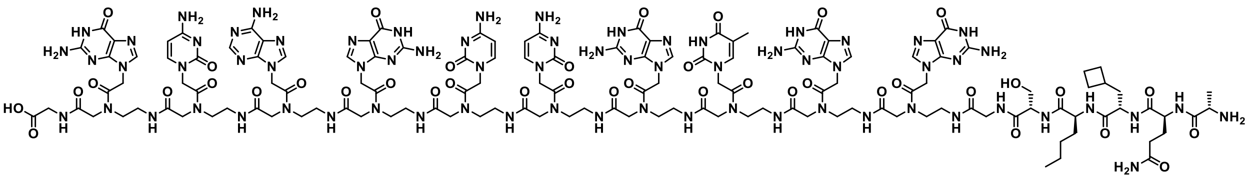

Chemical Formula: $C_{135}H_{179}N_{69}O_{40}$, **Exact Mass**: 3406.41, **Molecular Weight**: 3408.36.

MALDI-TOF; m/z found: 3408.17.

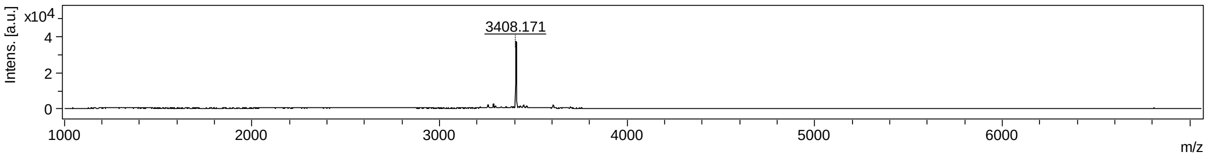

LCMS (ESI); RT= 1.26 min, $[M+3H]^{3+}$: 1037.00, $[M+4H]^{4+}$: 853.17.

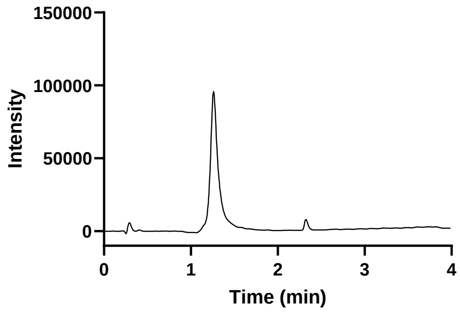

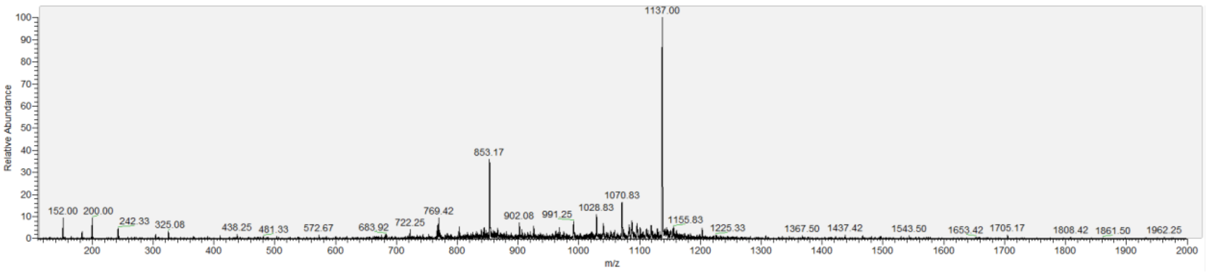

LHS2 (Figure 5)

HOOC-Gly-GCAGGAACCG-Gly-Ser-Nle-Leu-Gln-Ala-NH_2

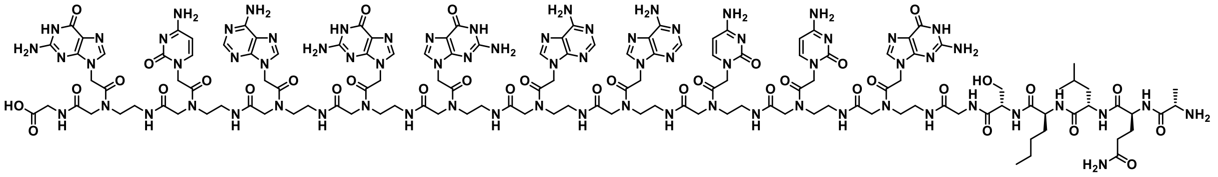

Chemical Formula: $C_{134}H_{178}N_{72}O_{37}$, **Exact Mass**: 3387.43, **Molecular Weight**: 3389.37

MALDI-TOF; m/z found: 3390.07.

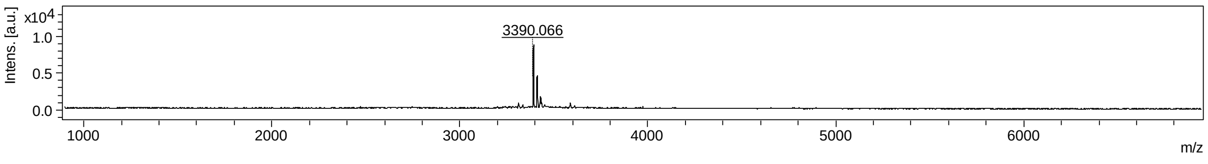

LCMS (ESI); RT= 1.25 min, $[M+3H]^{3+}$: 1130.83, $[M+4H]^{4+}$: 848.33.

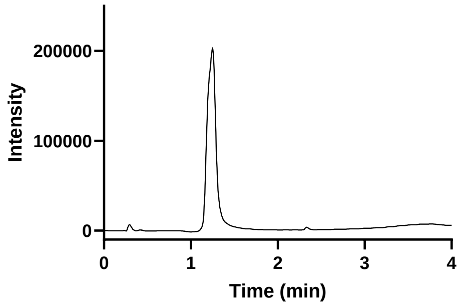

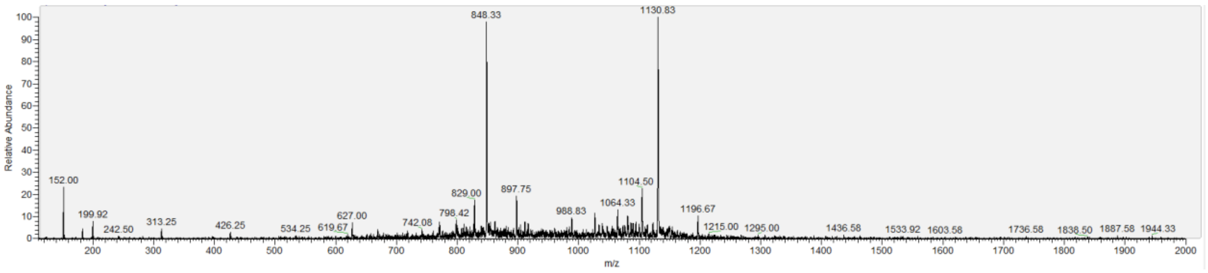

LHS3 (Figure 5)

HOOC-Gly-GCACGGCGCA-Gly-Ser-Nle-Ala-Gln-Ala-NH_2

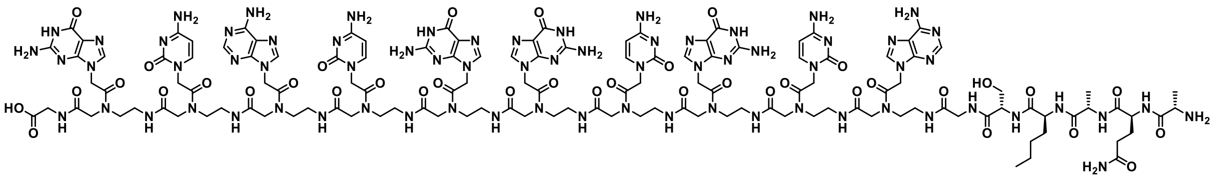

Chemical Formula: $C_{130}H_{172}N_{70}O_{38}$, **Exact Mass**: 3321.37, **Molecular Weight**: 3323.26.

MALDI-TOF; m/z found: 3324.12.

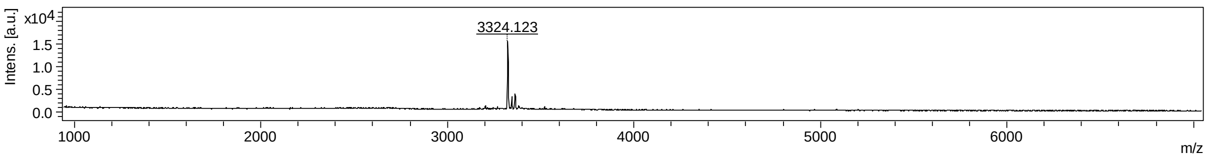

LCMS (ESI); RT= 1.13 min, $[M+3H]^{3+}$: 1108.58, $[M+4H]^{4+}$: 831.92.

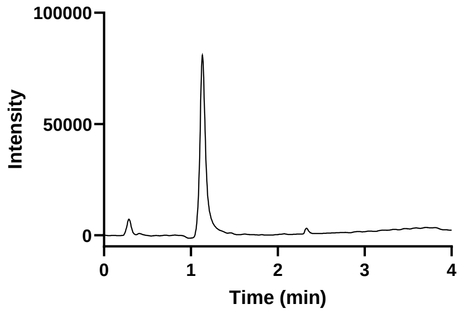

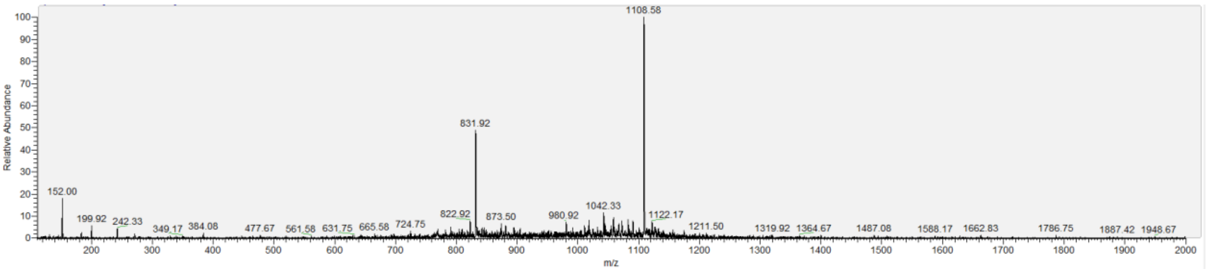

LHS4 (Figure 5)

HOOC-Gly-GCAAAGGCGA-Cba-Nle-Ser-Gln-Ala-Gly-NH_2

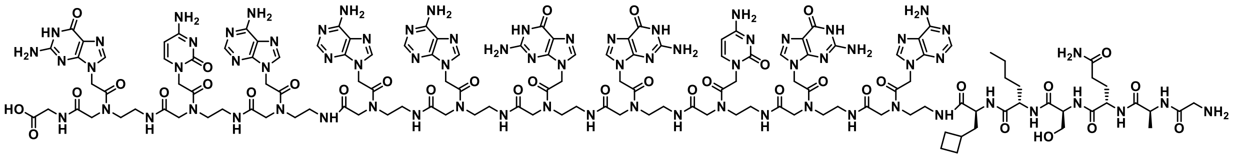

Chemical Formula: $C_{136}H_{178}N_{74}O_{36}$, **Exact Mass**: 2423.44, **Molecular Weight**: 3425.40.

MALDI-TOF; m/z found: 3425.26.

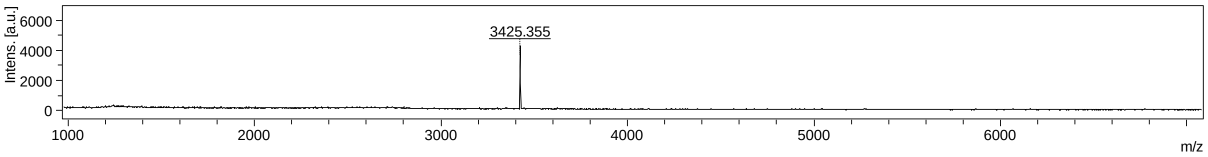

LCMS (ESI); RT= 1.27 min, $[M+3H]^{3+}$: 1142.75, $[M+4H]^{4+}$: 857.33.

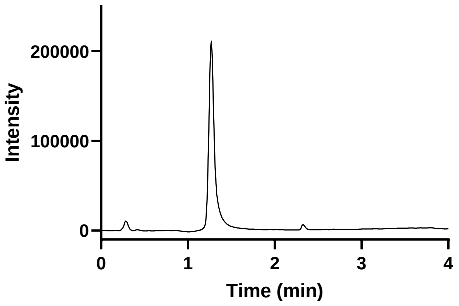

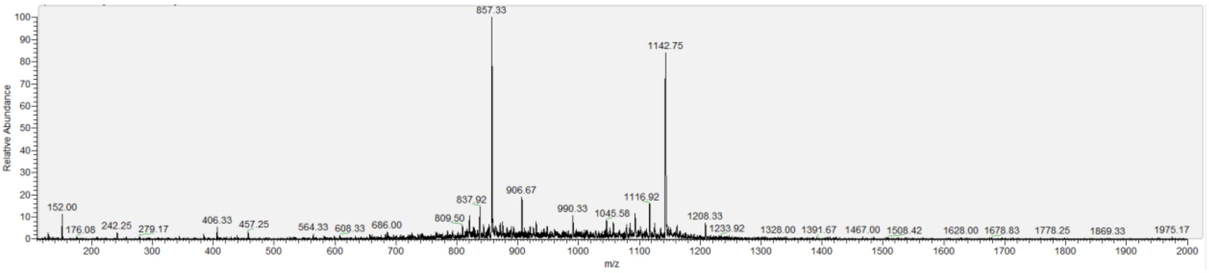

LHS5 (Figure 5)

HOOC-Gly-GCAGAACGGC- Gly-Ser-Nle-Ser-Gln-Ala-NH_2

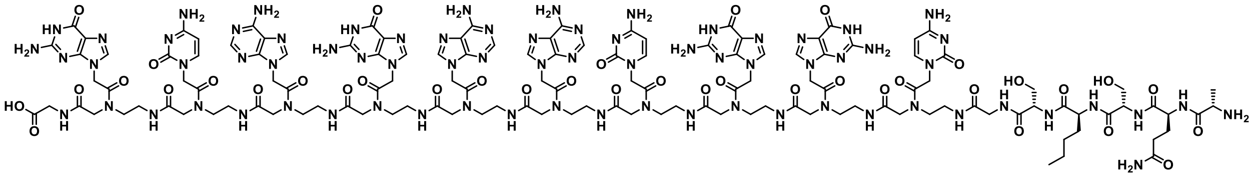

Chemical Formula: $C_{131}H_{172}N_{72}O_{38}$, **Exact Mass**: 3361.37, **Molecular Weight**: 3363.28.

MALDI-TOF; m/z found: 3363.21.

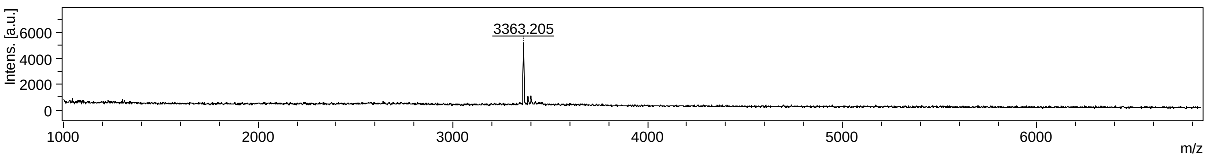

LCMS (ESI); RT= 1.15 min, $[M+3H]^{3+}$: 1121.92, $[M+4H]^{4+}$: 841.83.

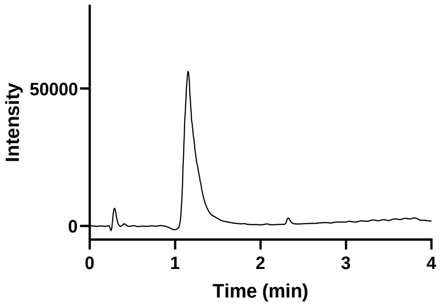

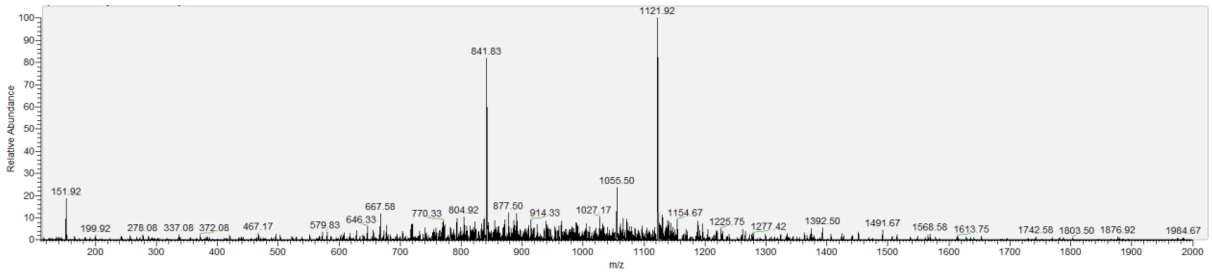

<u>**RHS1 (Figure 5)**</u>

HOOC-Trp-Tyr-Gln-Nle-Phe-Thr-Leu-Gly-GTGCGAACAG-Lys(PL-Biotin)-Atto647N

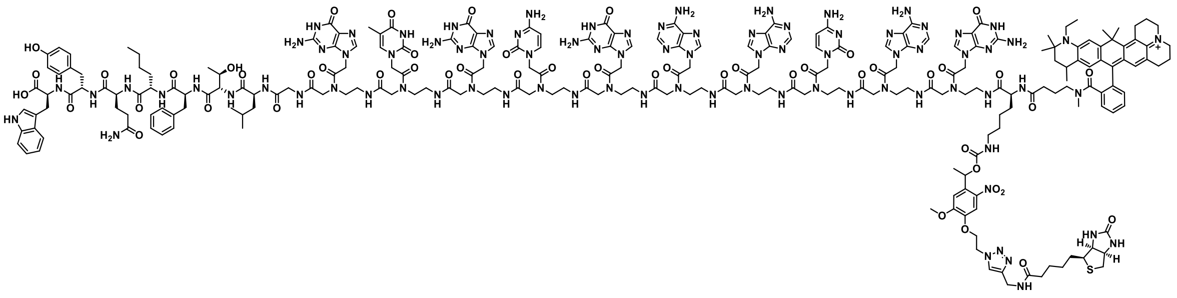

Chemical Formula: $C_{233}H_{294}N_{85}O_{51}S^{+}$, **Exact Mass**: 5130.27, **Molecular Weight**: 5133.52.

MALDI-TOF; m/z found: 4543.05. The photocleavable linker is cleaved by the MALDI laser. The observed m/z is therefore 590 g mol^{-1} less than the expected molecular weight. As a result, HRMS was also run to confirm the correct molecular weight (see the isotropic distribution below).

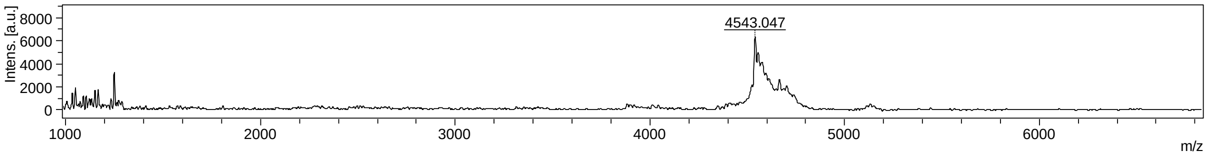

LCMS (ESI); RT= 2.21 min, $[M+4H]^{4+}$: 1027.58.

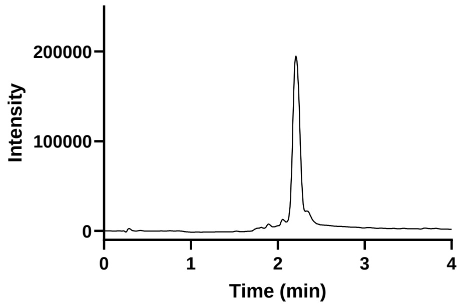

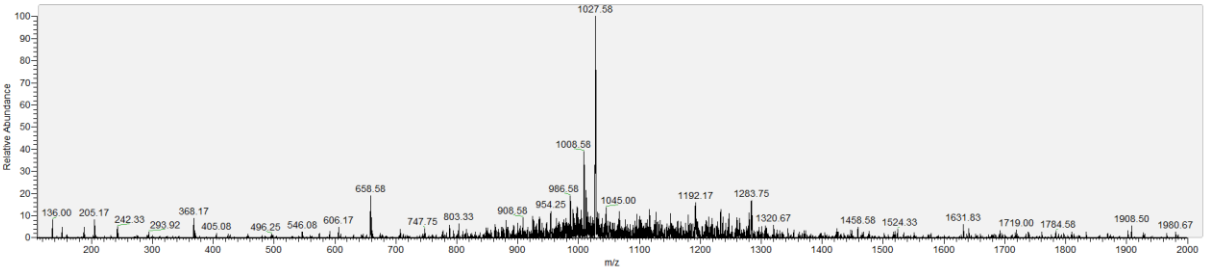

HRMS; $[M+4H]^{4+}$: 1284.07, $[M+5H]^{5+}$: 1027..

Predicted isotopic distribution for $[M+4H]^{4+}$:

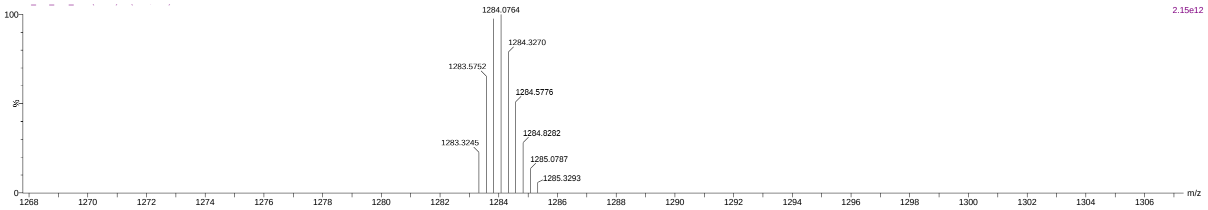

Observed isotopic distribution for $[M+4H]^{4+}$:

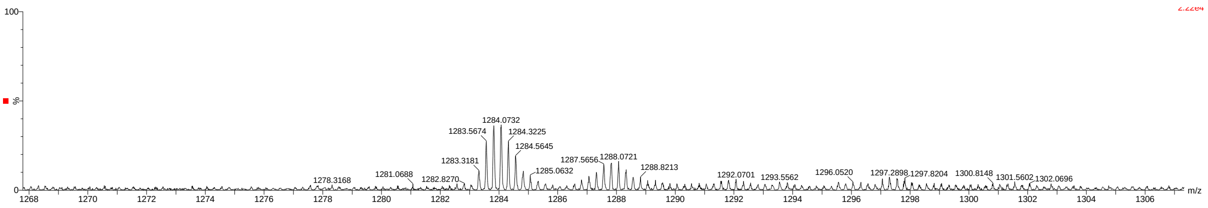

<u>RHS2 (Figure 5)</u>

HOOC-Trp-Tyr-Gln-Nle-PheF$_2$-Thr-Leu-Gly-GCAGAGACAG-Lys(PL-Biotin)-Atto647N

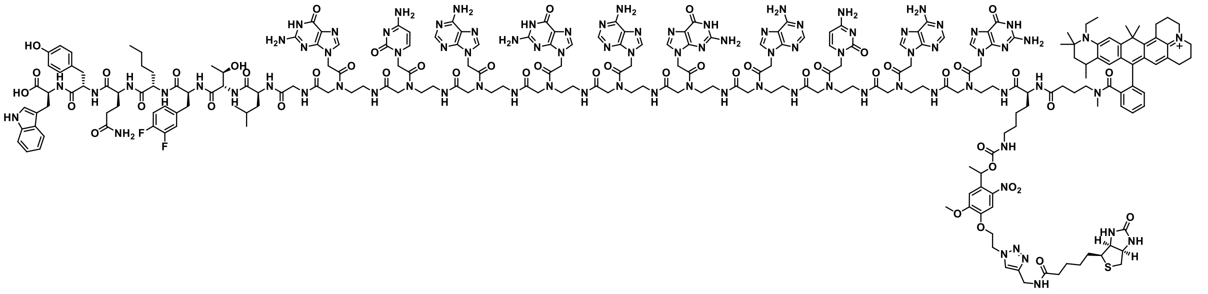

Chemical Formula: $C_{233}H_{291}F_2N_{88}O_{49}S^+$, **Exact Mass**: 5175.27, **Molecular Weight**: 5178.51.

MALDI-TOF; m/z found: 4588.82. The photocleavable linker is cleaved by the MALDI laser. The observed m/z is therefore 590 g mol^{-1} less than the expected molecular weight. As a result, HRMS was also run to confirm the correct molecular weight (see the isotropic distribution below).

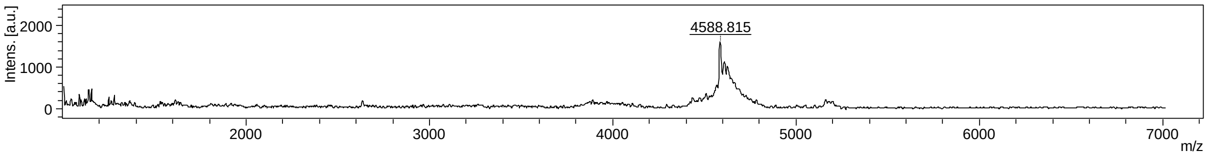

LCMS (ESI); RT= 2.19 min, [M+5H]$^{5+}$: 1036.50.

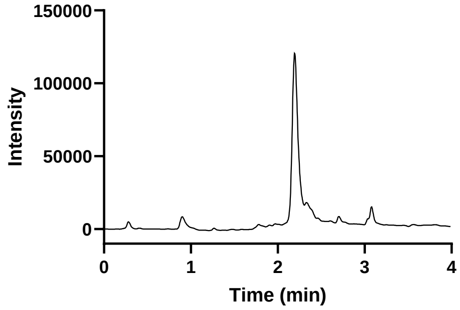

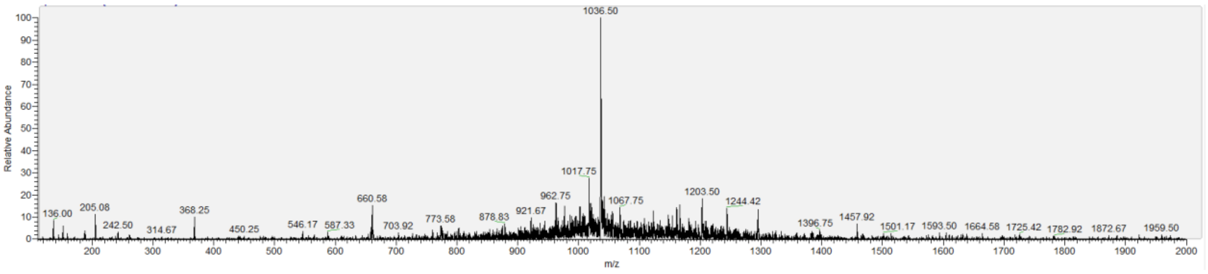

HRMS; [M+4H]$^{4+}$: 1295.07, [M+5H]$^{5+}$: 1036.26.

Predicted isotopic distribution for [M+4H]$^{4+}$:

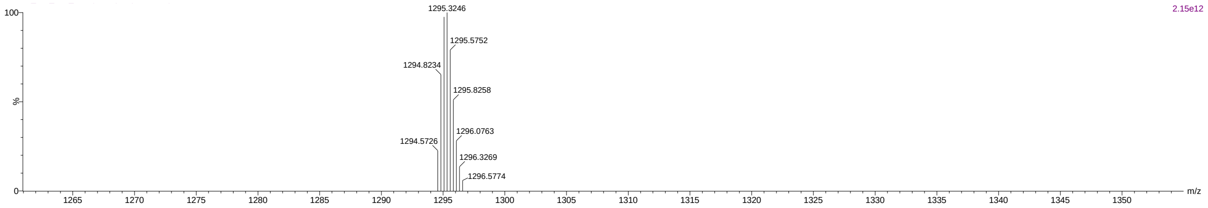

Observed isotopic distribution for [M+4H]$^{4+}$:

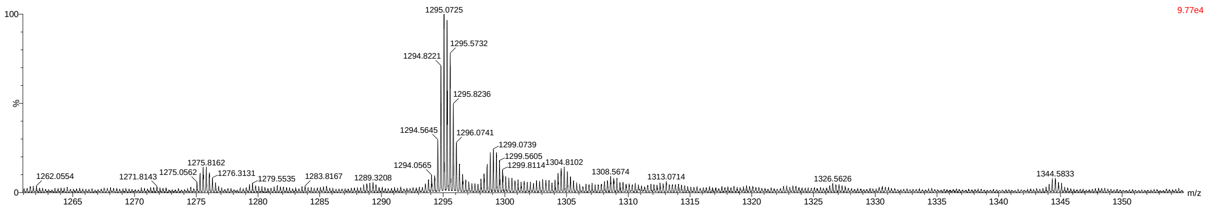

RHS3 (Figure 5)

HOOC-Ala-Tyr-Gln-Nle-Ala-Thr-Leu-Gly-ACGCAGGCAG-Lys(PL-Biotin)-Atto647N

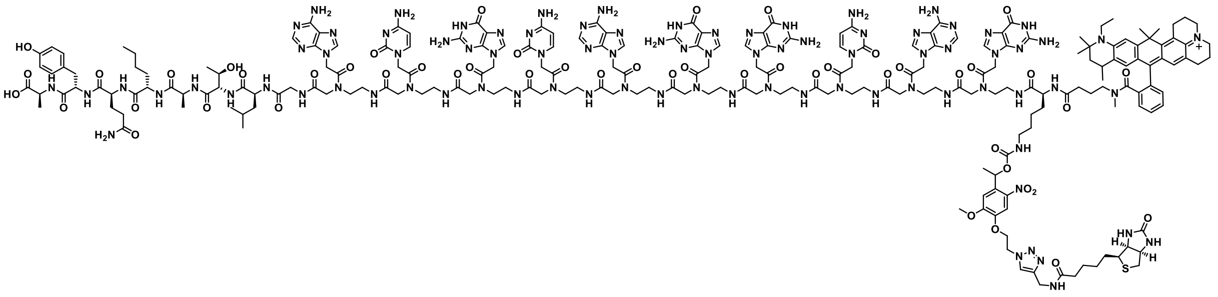

Chemical Formula: $C_{218}H_{284}N_{85}O_{50}S^{+}$**, Exact Mass**: 4924.20, **Molecular Weight**: 4927.27.

MALDI-TOF; m/z found: 4337.85. The photocleavable linker is cleaved by the MALDI laser. The observed m/z is therefore 590 g mol^{-1} less than the expected molecular weight. As a result, HRMS was also run to confirm the correct molecular weight (see the isotropic distribution below).

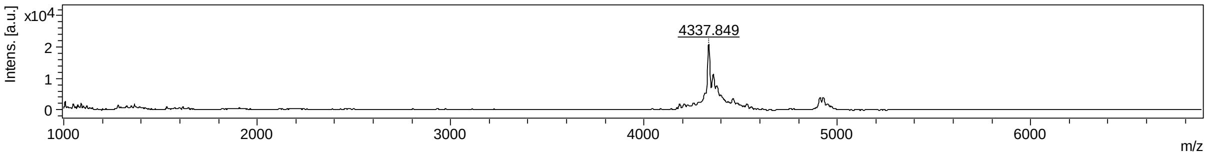

LCMS (ESI); RT= 2.03 min, $[M+4H]^{4+}$: 1232.33, $[M+5H]^{5+}$: 986.25.

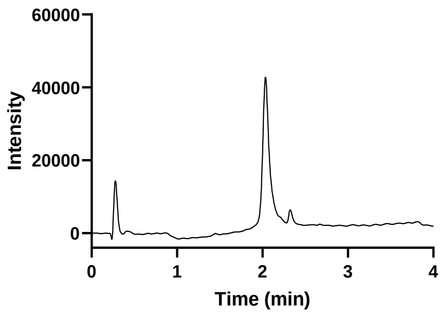

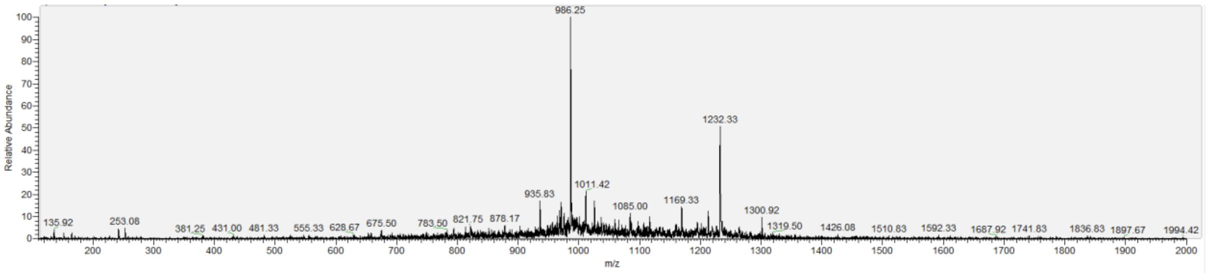

HRMS; $[M+4H]^{4+}$: 1232.30, $[M+5H]^{5+}$: 986.05.

Predicted isotopic distribution for $[M+4H]^{4+}$:

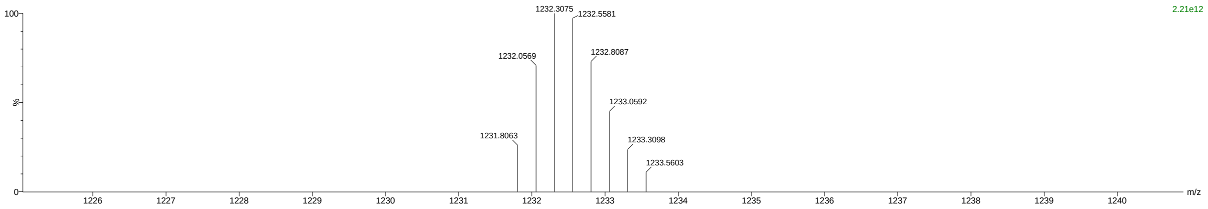

Observed isotopic distribution for $[M+4H]^{4+}$:

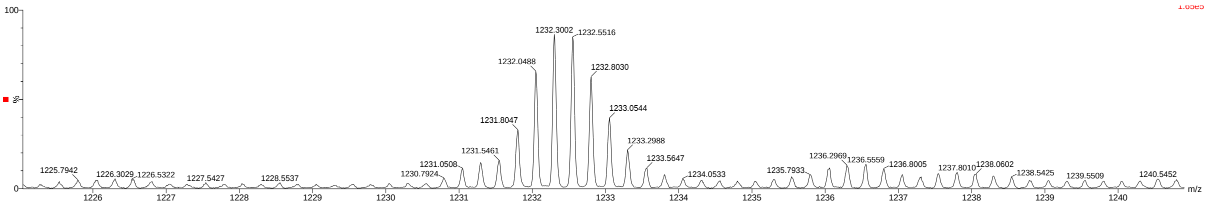

RHS4 (Figure 5)

HOOC-Gly-Gln-Tyr-Trp-Nle-Thr-Leu-Gly-CGAGACGCAG-Lys(PL-Biotin)-Atto647N

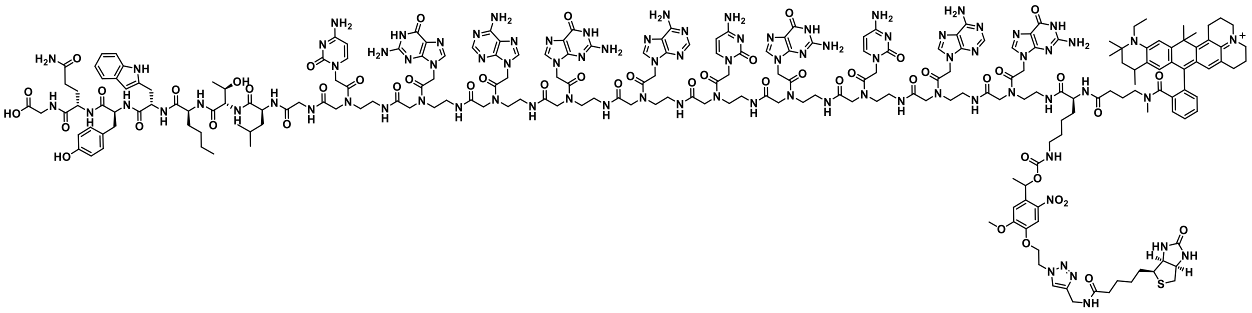

Chemical Formula: $C_{225}H_{287}N_{86}O_{50}S^{+}$, **Exact Mass**: 5025.23, **Molecular Weight**: 5028.38.

MALDI-TOF; m/z found: 4439.04. The photocleavable linker is cleaved by the MALDI laser. The observed m/z is therefore 590 g mol^{-1} less than the expected molecular weight. As a result, HRMS was also run to confirm the correct molecular weight (see the isotropic distribution below).

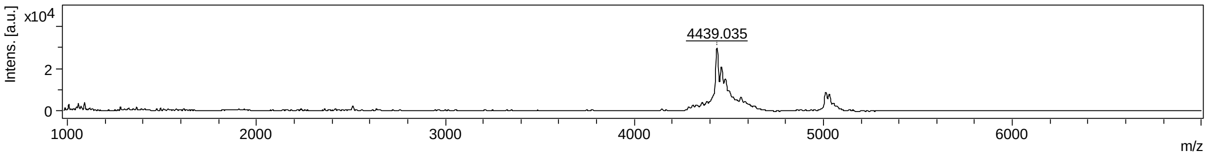

LCMS (ESI); RT= 2.06 min, $[M+4H]^{4+}$: 1257.92, $[M+5H]^{5+}$: 1006.33.

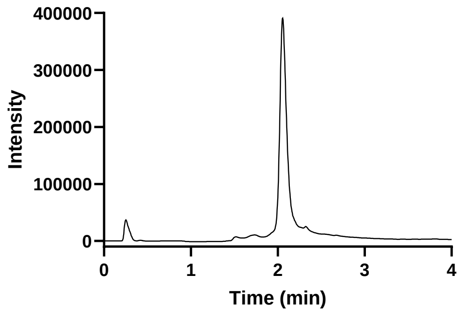

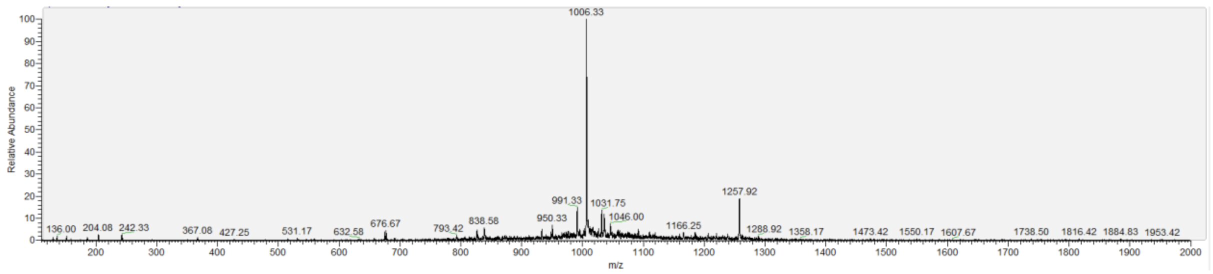

HRMS; $[M+4H]^{4+}$: 1257.56, $[M+5H]^{5+}$: 1006.24.

Predicted isotopic distribution for $[M+4H]^{4+}$:

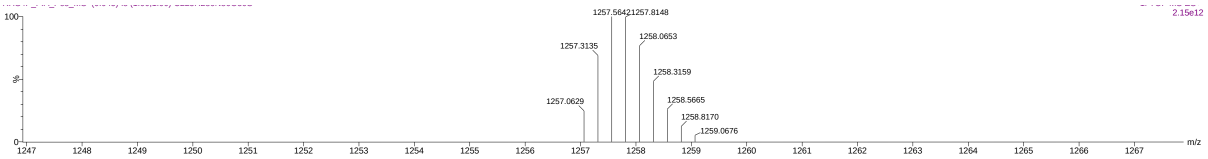

Observed isotopic distribution for $[M+4H]^{4+}$:

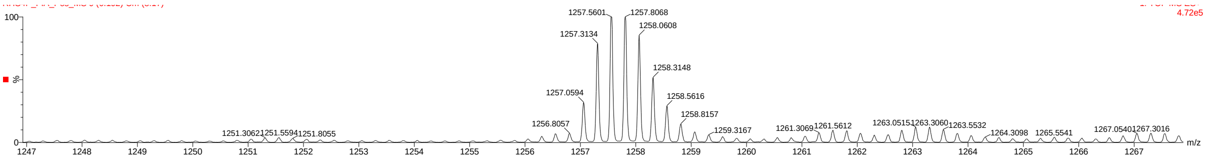

RHS5 (Figure 5)

HOOC-Ser-Tyr-Gln-Nle-Ser-Thr-Leu-Gly-AGCAGGCCAG-Lys(PL-Biotin)-Atto647N

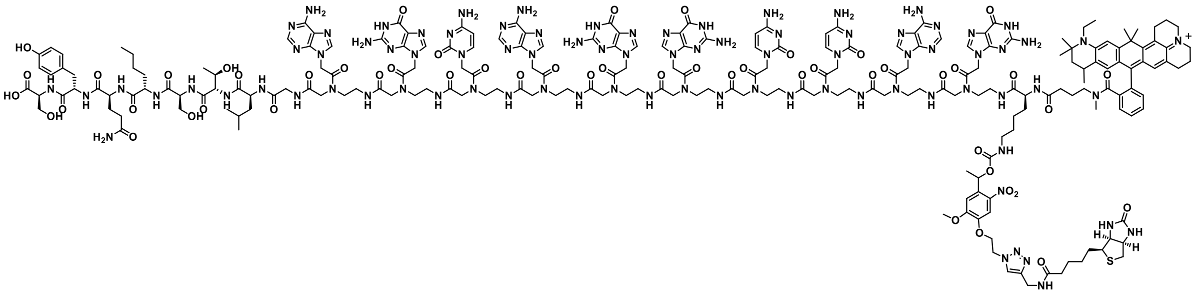

Chemical Formula: $C_{218}H_{284}N_{85}O_{52}S^+$, **Exact Mass**: 4956.19, **Molecular Weight**: 4959.27.

MALDI-TOF; m/z found: 4370.09. The photocleavable linker is cleaved by the MALDI laser. The observed m/z is therefore 590 g mol^{-1} less than the expected molecular weight. As a result, HRMS was also run to confirm the correct molecular weight (see the isotropic distribution below).

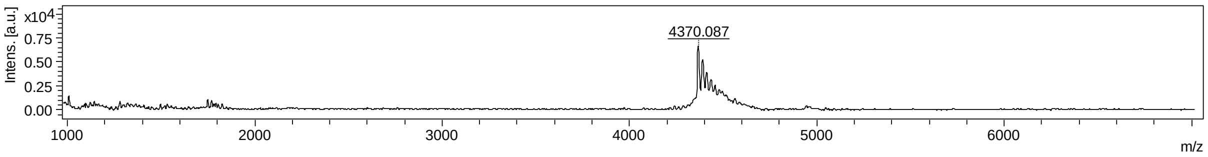

LCMS (ESI); RT= 2.01 min, $[M+4H]^{4+}$: 1240.58, $[M+5H]^{5+}$: 992.58.

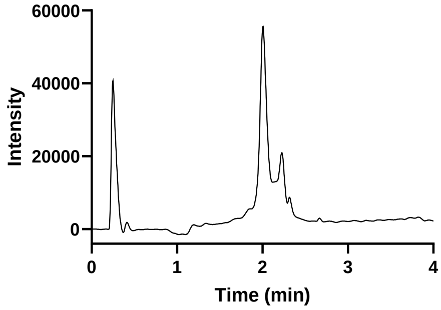

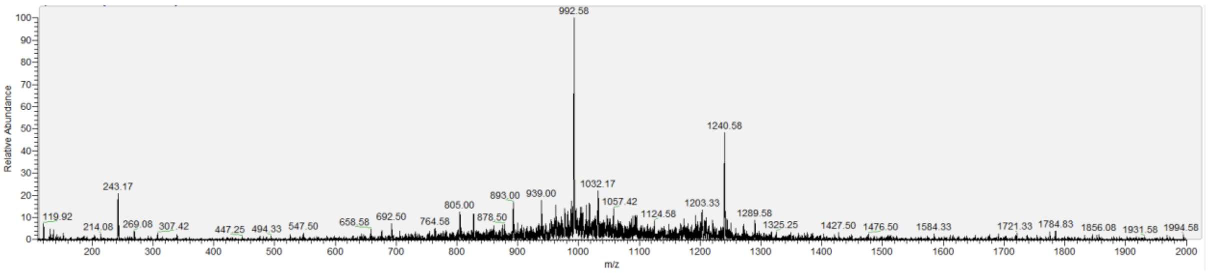

HRMS; $[M+4H]^{4+}$: 1240.29, $[M+5H]^{5+}$: 992.44.

Predicted isotopic distribution for $[M+4H]^{4+}$:

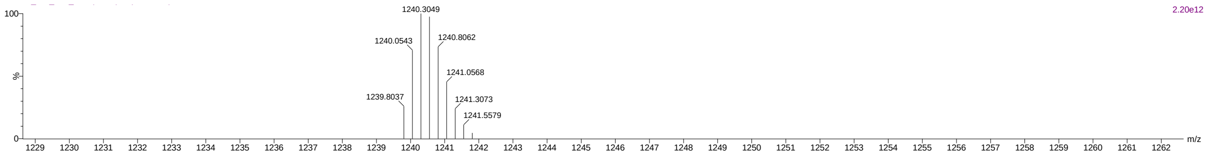

Observed isotopic distribution for $[M+4H]^{4+}$:

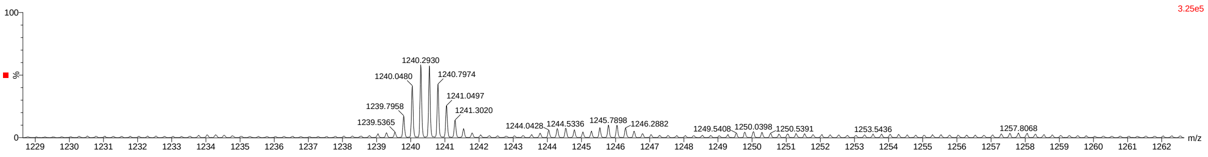

PNA-constrained Peptide 1.1 (Figure 6)

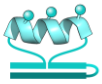 NH2-Arg-CGGC-Gly-Ser-Nle-Cba-Gln-Ala-Trp-Tyr-Gln-Nle-Phe-Thr-Leu-Gly-ATCC-Arg-PEG-Arg-GGATGCCG-Arg-Ac

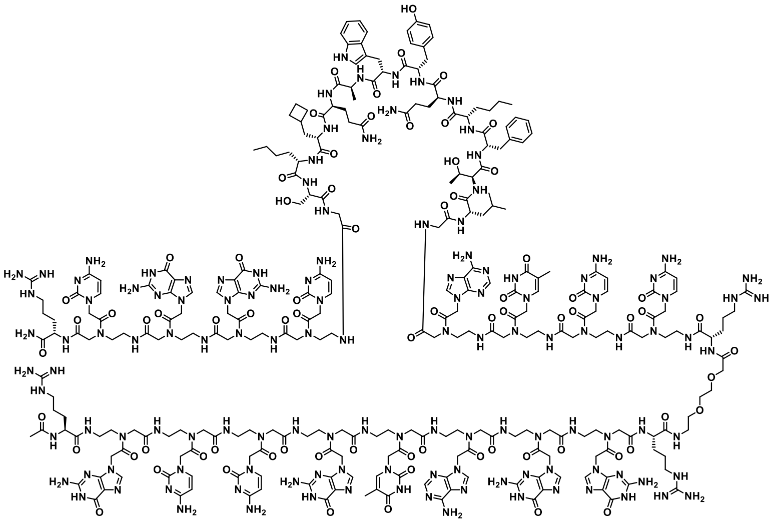

Chemical Formula: $C_{280}H_{385}N_{129}O_{75}$, **Exact Mass**: 6754.03, **Molecular Weight**: 6757.988.

MALDI-TOF; m/z found: 6758.98.

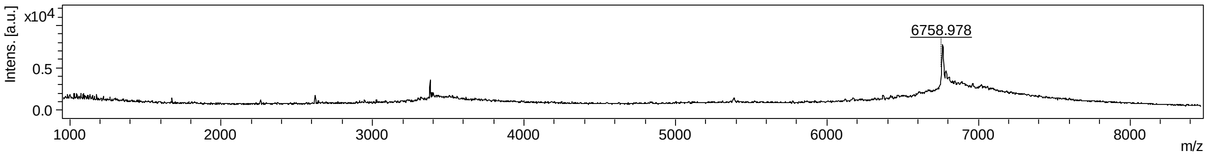

LCMS (ESI); RT= 1.89 min, $[M+5H]^{5+}$: 1352.75, $[M+6H]^{6+}$: 1127.33, $[M+7H]^{7+}$: 966.42, $[M+8H]^{8+}$: 845.92.

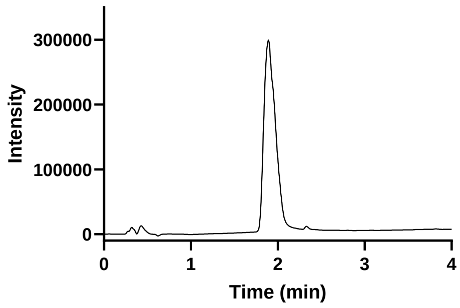

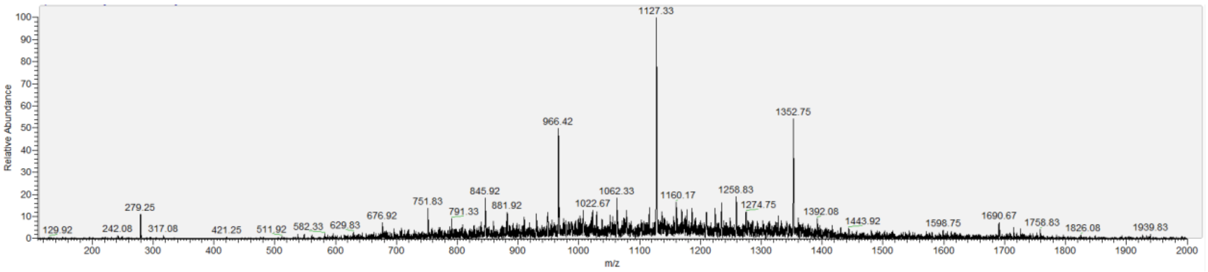

PNA-constrained Peptide 1.2 (Figure 6)

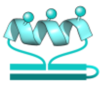 NH2-Arg-CGGC-Gly-Ser-Nle-Cba-Gln-Ala-Trp-Tyr-Gln-Nle-PheF$_2$-Thr-Leu-Gly-ATCC-Arg-PEG-Arg-GGATGCCG-Arg- NHAc

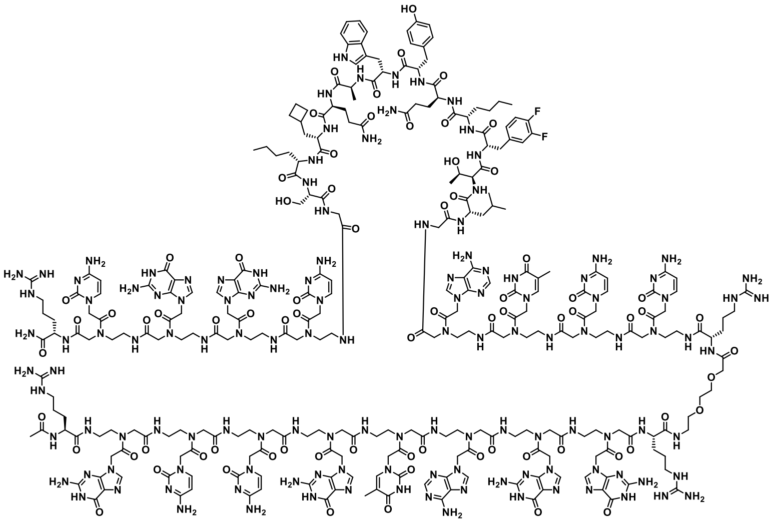

Chemical Formula: $C_{280}H_{383}F_2N_{129}O_{75}$, **Exact Mass**: 6790.01, **Molecular Weight**: 6793.97.

MALDI-TOF; m/z found: 6794.76.

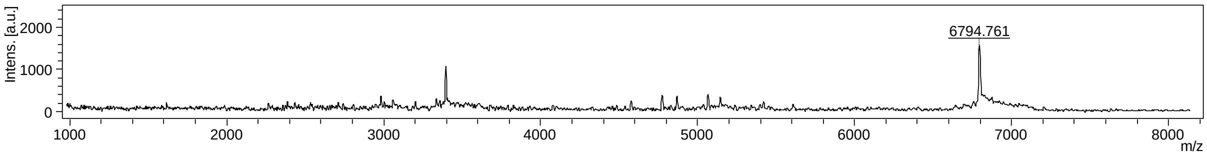

LCMS (ESI); RT= 1.90 min, $[M+5H]^{5+}$: 1359.83, $[M+6H]^{6+}$: 1133.42, $[M+7H]^{7+}$: 971.50, $[M+8H]^{8+}$: 850.33.

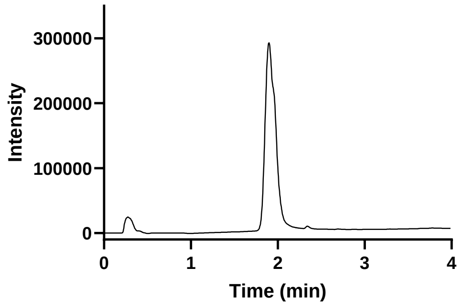

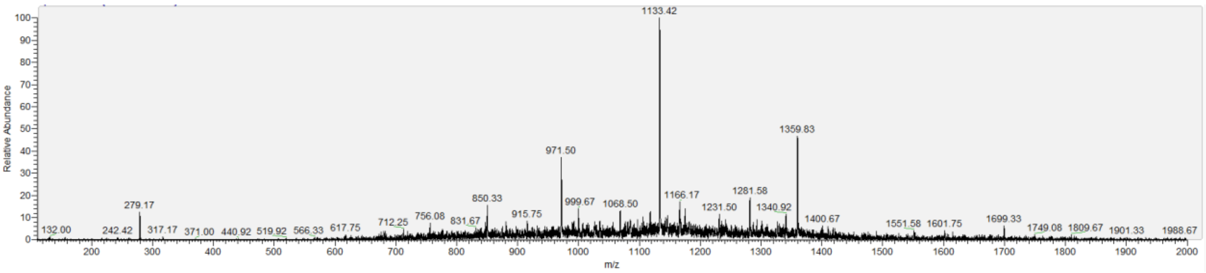

PNA-constrained Peptide 2.1 (Figure 6)

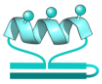 NH2-Arg-CGGC-Gly-Ser-Nle-Leu-Gln-Ala-Trp-Tyr-Gln-Nle-Phe-Thr-Leu-Gly-ATCC-Arg-PEG-Arg-GGATGCCG-Arg-NHAc

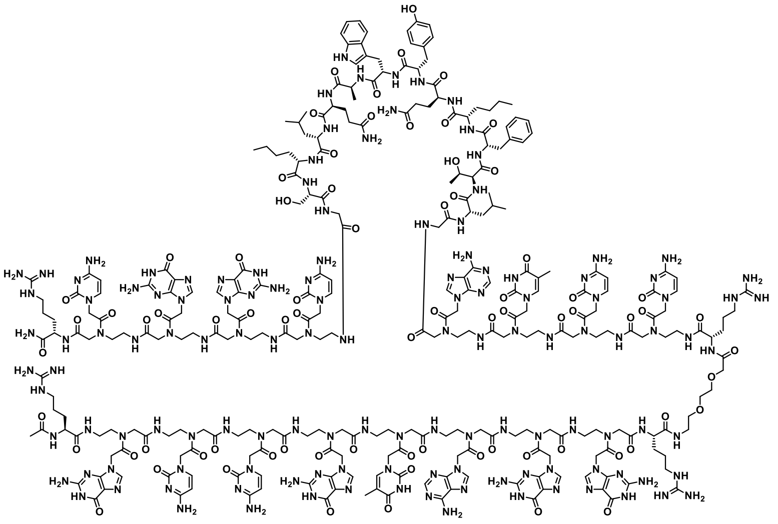

Chemical Formula: $C_{279}H_{385}N_{129}O_{75}$, **Exact Mass**: 6742.03, **Molecular Weight**: 6745.98.

MALDI-TOF; m/z found: 6746.58.

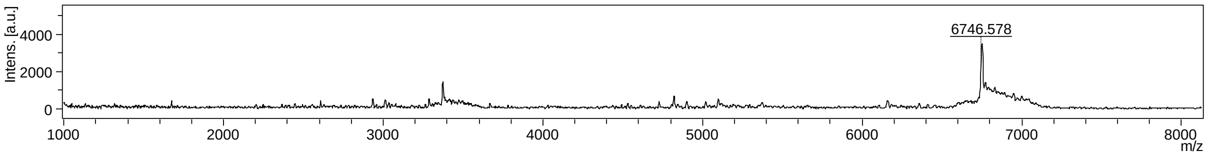

LCMS (ESI); RT= 1.88 min, $[M+5H]^{5+}$: 1350.25, $[M+6H]^{6+}$: 1125.33, $[M+7H]^{7+}$: 964.58, $[M+8H]^{8+}$: 844.25.

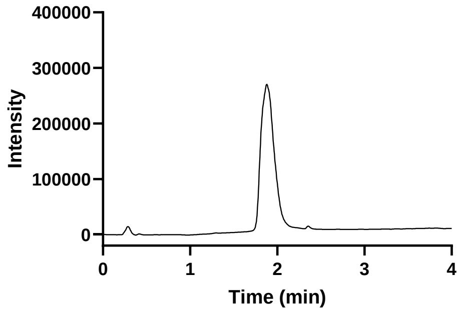

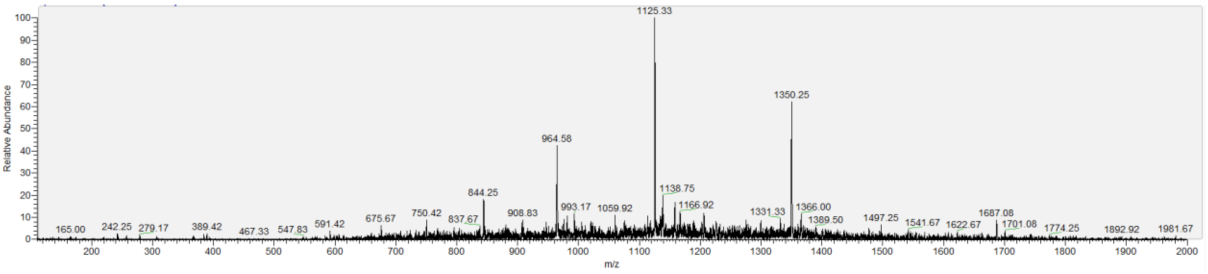

PNA-constrained Peptide 2.2 (Figure 6)

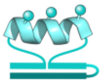
NH2-Arg-CGGC-Gly-Ser-Nle-Leu-Gln-Ala-Trp-Tyr-Gln-Nle-PheF_2-Thr-Leu-Gly-ATCC-Arg-PEG-Arg-GGATGCCG-Arg-NHAc

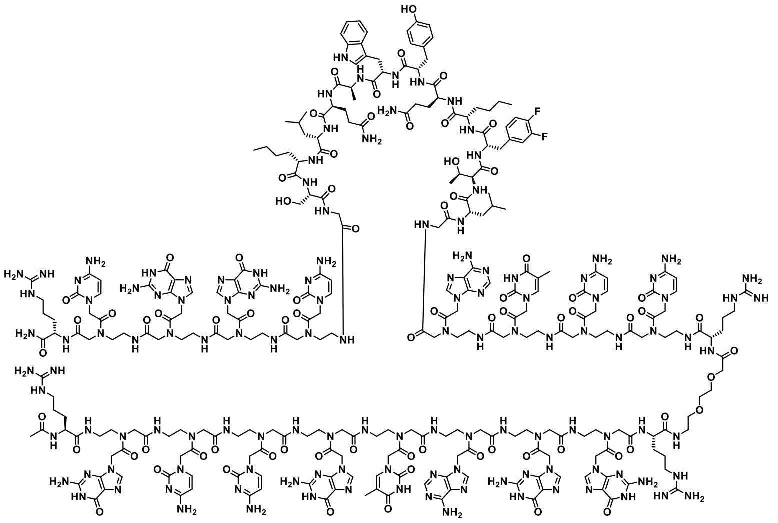

Chemical Formula: $C_{279}H_{383}F_2N_{129}O_{75}$, **Exact Mass**: 6778.01, **Molecular Weight**: 6781.96.

MALDI-TOF; m/z found: 6783.76.

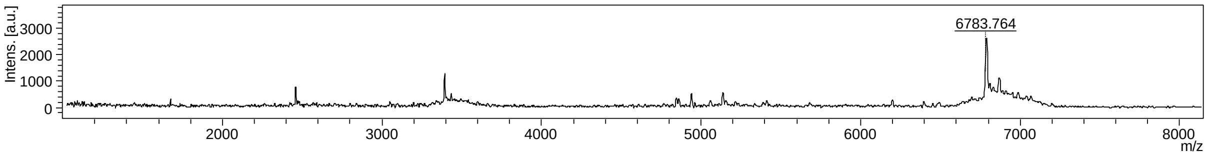

LCMS (ESI); RT= 1.93 min, $[M+5H]^{5+}$: 1357.50, $[M+6H]^{6+}$: 1131.42, $[M+7H]^{7+}$: 969.83, $[M+8H]^{8+}$: 848.75, $[M+8H]^{8+}$: 754.58.

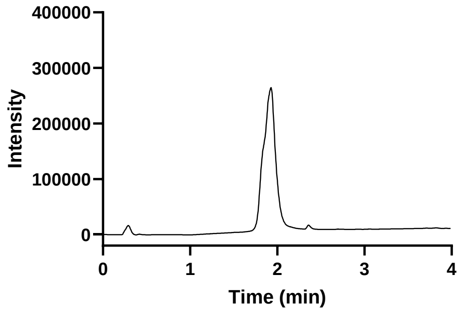

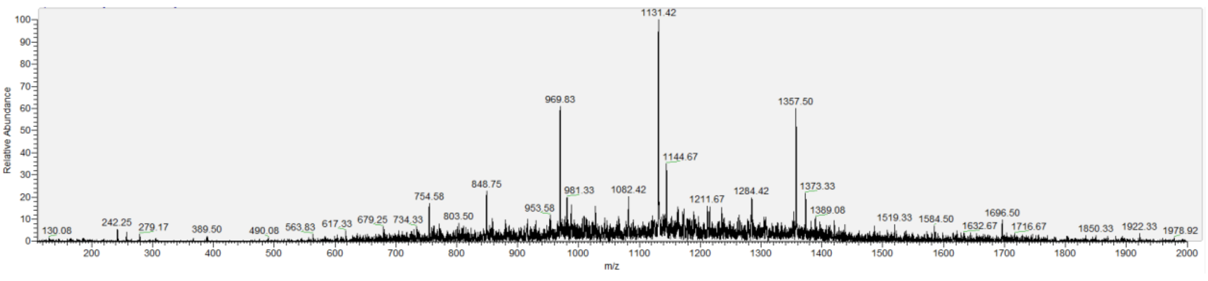

<u>**PNA-constrained Peptide 4.1**</u>

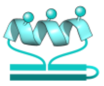 NH2-Arg-CGGC-Cba-Nle-Ser-Gln-Ala-Gly-Trp-Tyr-Gln-Nle-Phe-Thr-Leu-Gly-ATCC-Arg-PEG-Arg-GGATGCCG-Arg-NHAc

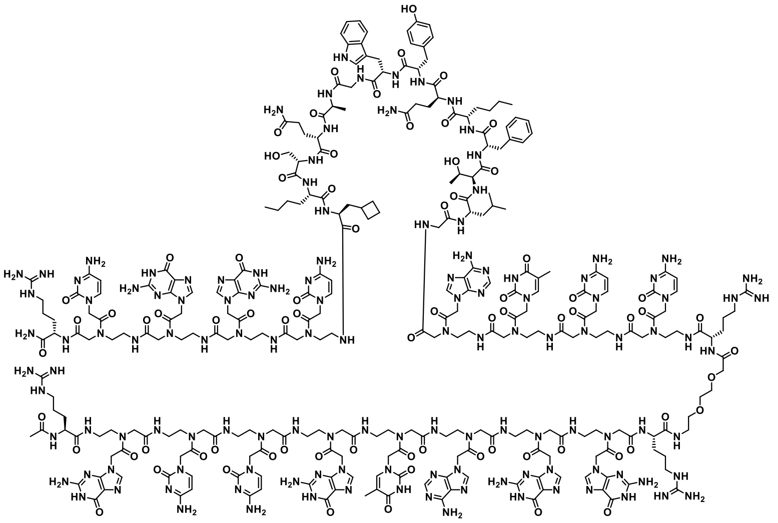

Chemical Formula: $C_{280}H_{385}N_{129}O_{75}$, **Exact Mass**: 6754.03, **Molecular Weight**: 6757.99.

MALDI-TOF; m/z found: 6758.39.

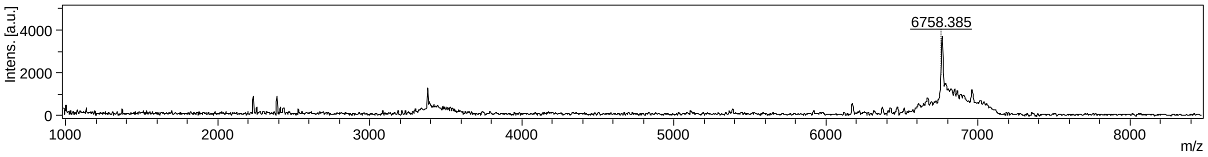

LCMS (ESI); RT= 1.74 min, $[M+5H]^{5+}$: 1352.50, $[M+6H]^{6+}$: 1127.42, $[M+7H]^{7+}$: 966.42, $[M+8H]^{8+}$: 845.67.

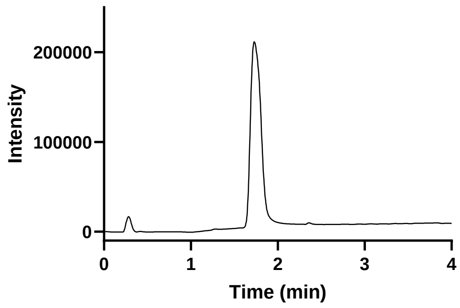

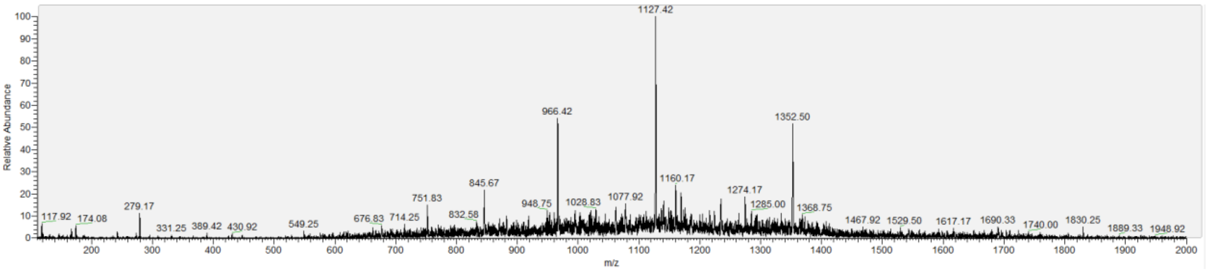

PNA-constrained Peptide 4.4

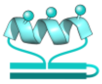 NH2-Arg-CGGC-Cba-Nle-Ser-Gln-Ala-Gly-Gly-Gln-Tyr-Trp-Nle-Thr-Leu-Gly-ATCC-Arg-PEG-Arg-GGATGCCG-Arg-NHAc

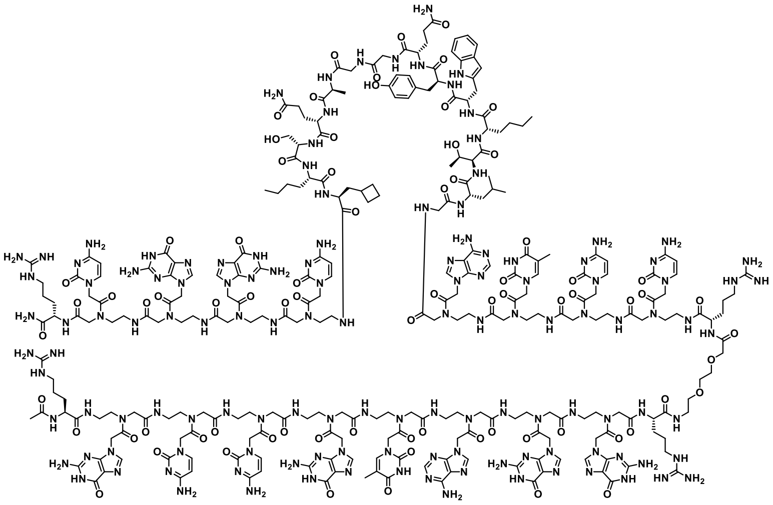

Chemical Formula: $C_{273}H_{379}N_{129}O_{75}$, **Exact Mass**: 6663.98, **Molecular Weight**: 6667.86.

MALDI-TOF; m/z found: 6667.79.

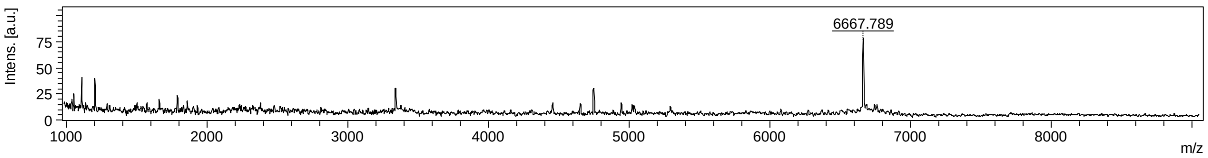

LCMS (ESI); RT= 1.61 min, $[M+5H]^{5+}$: 1334.41, $[M+6H]^{6+}$: 1112.42, $[M+7H]^{7+}$: 953.58, $[M+8H]^{8+}$: 834.42.

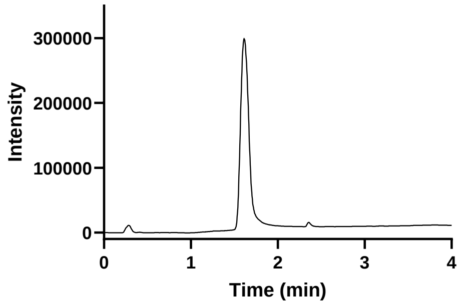

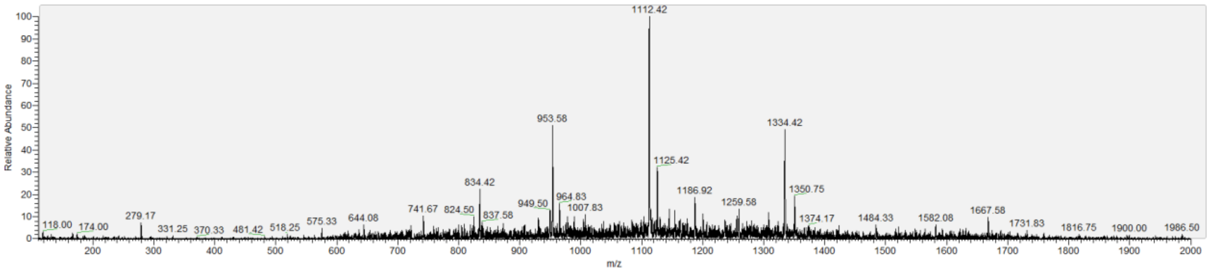

iii) Peptides

1.1 Linear (Figure 6)

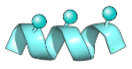

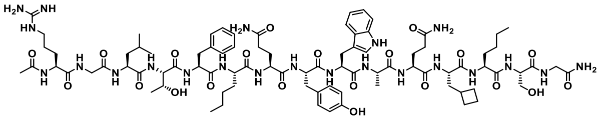

Chemical Formula: $C_{86}H_{128}N_{22}O_{21}$, **Exact Mass**: 1804.96, **Molecular Weight**: 1806.10.

MALDI-TOF; m/z found: 1807.06.

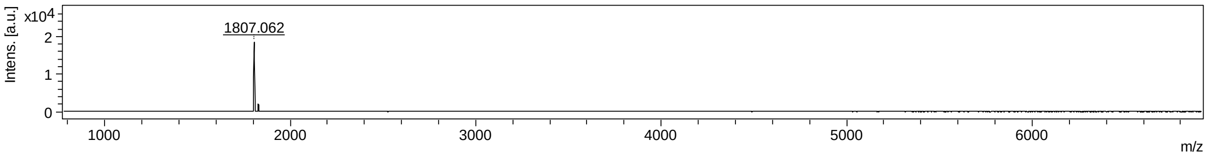

LCMS (ESI); RT= 2.58 min, $[M+1H]^{1+}$: 1086.83, $[M+2H]^{2+}$: 903.58.

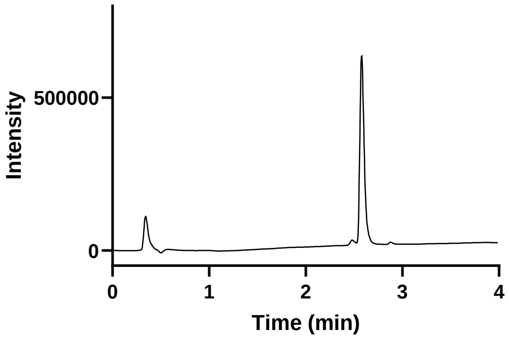

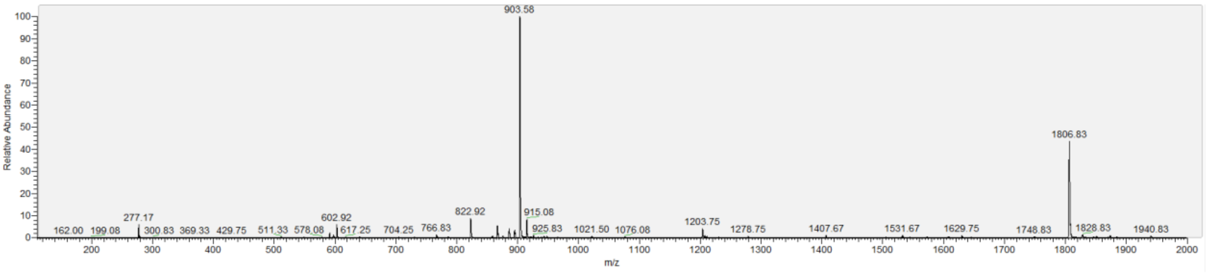

1.1 Cyclic (Figure 6)

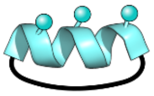

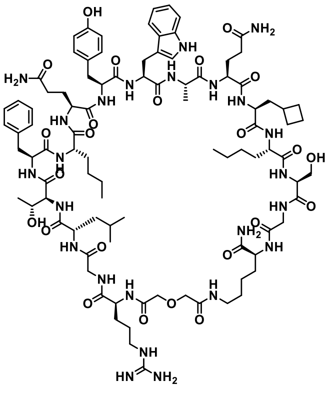

Chemical Formula: $C_{94}H_{140}N_{24}O_{24}$, **Exact Mass**: 1989.05, **Molecular Weight**: 1990.30.

MALDI-TOF; m/z found: 1991.43.

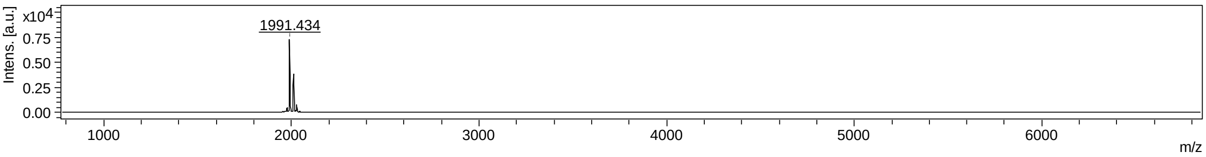

LCMS (ESI); RT= 1.54 min, $[M+1H]^{1+}$: 1991.00, $[M+2H]^{2+}$: 996.00.

Acquired with a modified method: Increasing gradient (30-100%) of acetonitrile 0.01% TFA in water 0.01%.

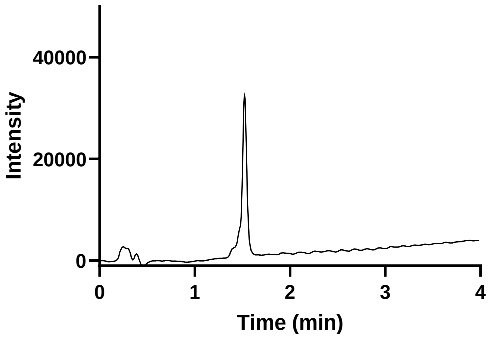

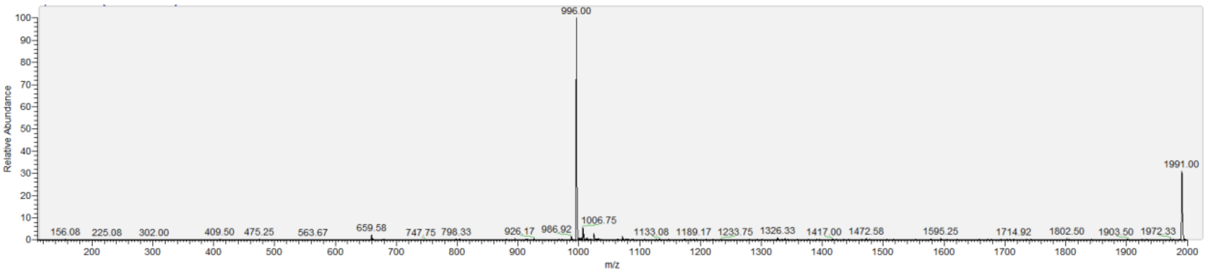

10. Full gels from figures

Gels in Figure 2

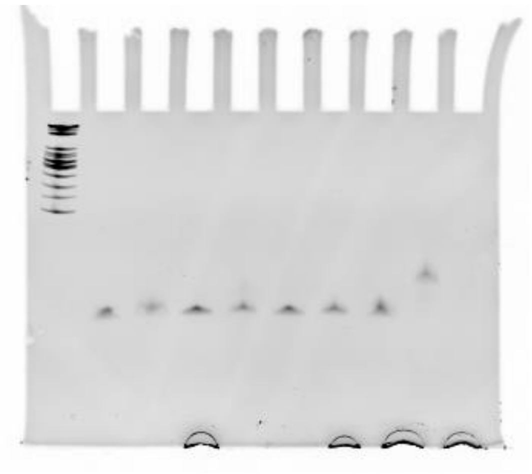

1: Ladder
2: DNA1
3: DNA1+EDC
4: DNA1+PNA2+PNA3
5: DNA1+EDC+PNA2+PNA3
6: DNA1
7: DNA1+EDC
8: DNA1+PNA1+PNA4
9: DNA1+EDC+PNA1+PNA4

10 fmol of DNA (10 µL of 1 µM reaction) was loaded in each lane. 1 µL 100 bp ladder was loaded in lane 1.

Denaturing (8M UREA) 15% PAGE, Ethidium bromide staining.

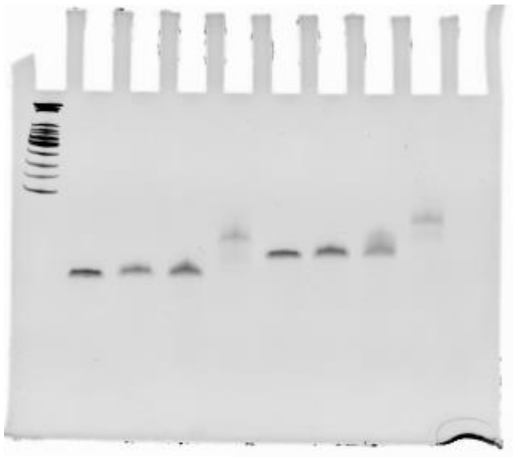

1:Ladder
2: DNA2
3: DNA2+EDC
4: DNA2+PNA1+PNA2+PNA4
5: DNA2+EDC+PNA1+PNA2+PNA4
6: DNA3
7: DNA3+EDC
8: DNA3+PNA1+PNA2+PNA3+PNA4
9: DNA3+EDC+ PNA1+PNA2+PNA3+PNA4

10 fmol of DNA (10 µL of 1 µM reaction) was loaded in each lane. 1 µL 100 bp ladder was loaded in lane 1.

Denaturing (8M UREA) 15% PAGE, Ethidium bromide staining.

Gels in Figure 3

Ethidium bromide staining

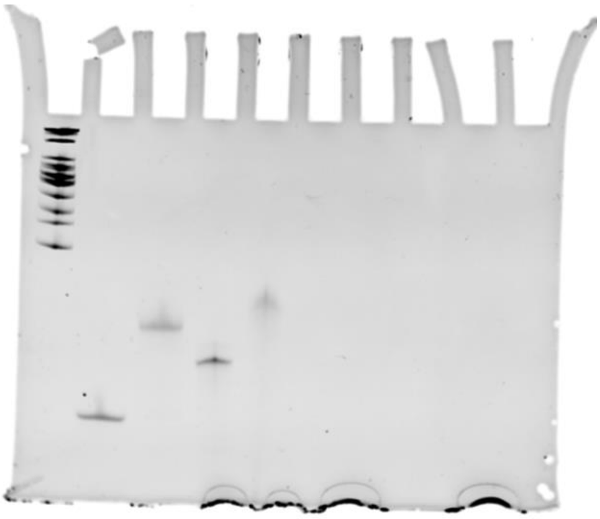

1:Ladder
2: DNA1
3: DNA1+BinderLHS+BinderRHS
4: DNA4
5: DNA4+Non-BinderLHS+Non-BinderRHS

10 fmol of DNA (10 µL of 1 µM reaction) was loaded in each lane. 1 µL 100 bp ladder was loaded in lane 1.

Denaturing (8M UREA) 15% PAGE,

Cy3 Fluorescence

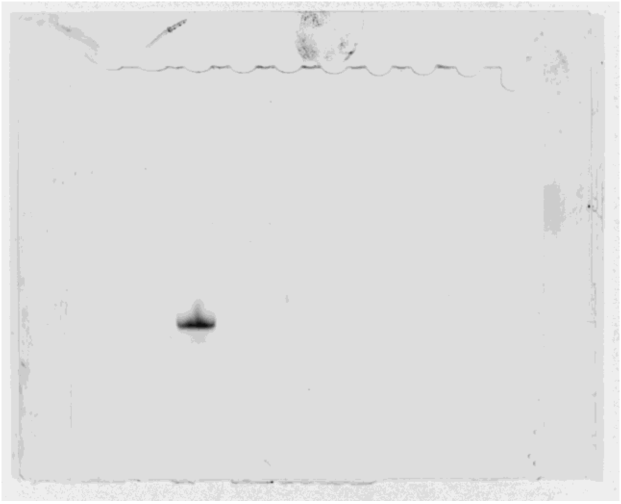

Atto647N Fluorescence

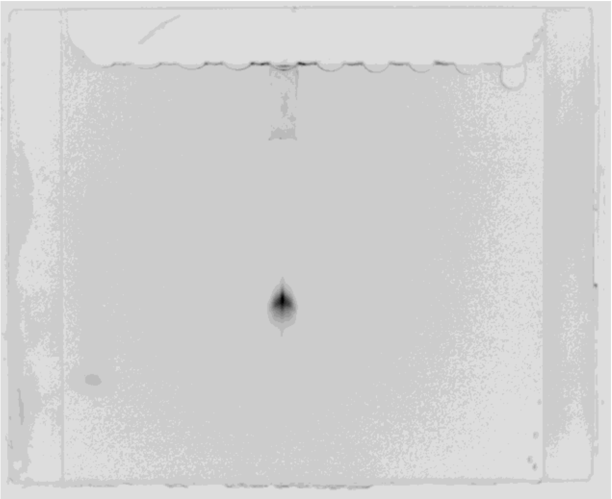

Gels in Figure 4

Ethidium bromide staining

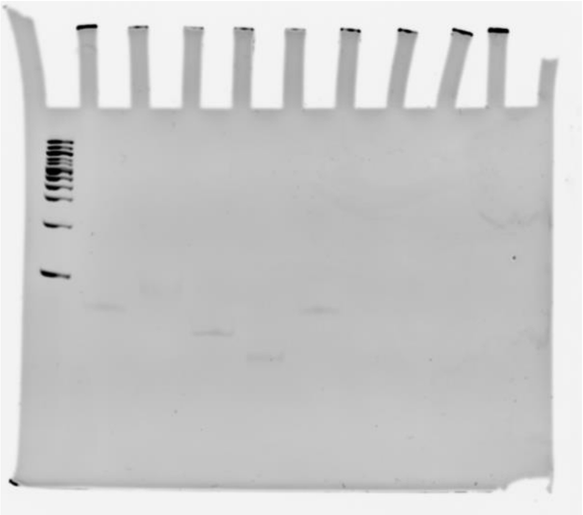

1:Ladder
2: Hairpin DNA Library (0.45 fmol)
3: DNA-PNA Library (0.45 fmol)
4: Double stranded DNA (0.2 fmol)
5: Single stranded DNA (0.6 fmol)
6: Hairpin DNA Library (0.45 fmol)

0.2-0.6 fmol of constructs were loaded in each lane – amount variation is due to EtBr staining variation (double strand DNA stains more efficiently that single strand DNA). 1 µL 100 bp ladder was loaded in lane 1.

Native 15% PAGE

Atto647N Fluorescence

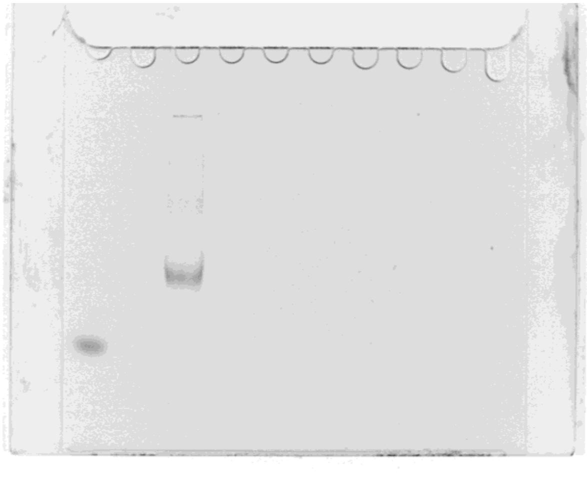

Gel in Supplementary Figure 2

Ethidium bromide staining

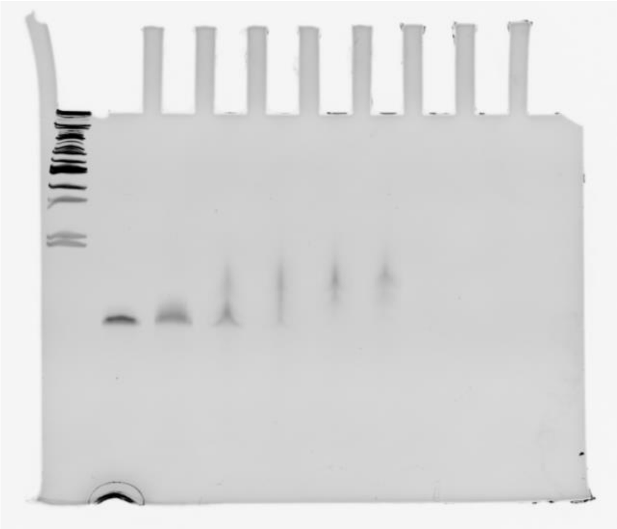

1:Ladder
2: DNA3
3: DNA3+PNA1+PNA2+PNA3+PNA4
4: DNA3+PNA1+PNA2+PNA3+PNA4+EDC(1mM)
5: DNA3+PNA1+PNA2+PNA3+PNA4+EDC(5mM)
6: DNA3+PNA1+PNA2+PNA3+PNA4+EDC(50mM)
7: DNA3+PNA1+PNA2+PNA3+PNA4+EDC(100mM)

10 fmol of DNA (10 µL of 1 µM reaction) was loaded in each lane. 1 µL 100 bp ladder was loaded in lane 1.

Denaturing (8M UREA) 15% PAGE, Ethidium bromide staining.

11. References

(1) Lau, Y. H.; Wu, Y.; Rossmann, M.; Tan, B. X.; de Andrade, P.; Tan, Y. S.; Verma, C.; McKenzie, G. J.; Venkitaraman, A. R.; Hyvönen, M.; et al. Double Strain-Promoted Macrocyclization for the Rapid Selection of Cell-Active Stapled Peptides. *Angewandte Chemie International Edition* **2015**, *54* (51), 15410-15413. DOI: https://doi.org/10.1002/anie.201508416.
